# Advancing the psychology of social class with large-scale replications in four countries

**DOI:** 10.1038/s41562-025-02234-1

**Published:** 2025-07-15

**Authors:** Anatolia Batruch, Nicolas Sommet, Frédérique Autin

**Affiliations:** 1 LIVES Centre, University of Lausanne, Lausanne, Switzerland; 2 Centre de Recherches sur la Cognition et l’Apprentissage (UMR 7295), Université de Poitiers, Université de Tours, CNRS, Poitiers, France

**Keywords:** Social policy, Sociology, Psychology, Human behaviour, Decision making

## Abstract

Theoretical models have been developed to understand how social class influences individual thoughts, feelings and behaviours. However, the validity of these models is threatened by the prevailing use of small, non-diverse samples and flexible measurement practices. We preregistered replications of 35 key hypotheses from 17 correlational and 5 experimental studies, and collected large, quota-based or probability samples from the USA, France, Switzerland and India (*N*_total_ = 33,536). Our analysis yielded three central findings: (1) ~50% of the effects were successfully replicated; (2) conclusions were consistent across different operationalizations of social class, although objective indicators yielded smaller estimates (for example, income and education); and (3) half of the effects were moderated—mostly strengthened—by social class identification, system-justification beliefs or local income inequality. Overall, hypotheses based on differences between social class contexts in terms of constraints, uncertainty and status were well supported. However, hypotheses based on models positing social class differences in psychological orientations towards ‘the self’ versus ‘others and the environment’ received less support. We conclude that these models need to be reassessed as individuals from higher social classes seem more oriented towards both themselves and others. The Stage 1 protocol for this Registered Report was accepted in principle on 29 October 2021. The protocol, as accepted by the journal, can be found at 10.17605/OSF.IO/B6Y8R.

## Abstract (web summary)

Social class is assumed to be associated with many different individual outcomes and behaviours. This Registered Report aimed to replicate 35 key hypotheses from 17 correlational and 5 experimental studies using quota-based or probability samples.

## Main

As the topic of socio-economic inequality has gained considerable political momentum in the past decade, psychologists and behavioural scientists have finally turned their attention to the study of social class, developing various theoretical frameworks to account for the role of social class in shaping the way individuals think, feel and behave^1–5^. These theoretical frameworks describe social class as a relative social position defined by unequal access to economic, cultural, social and/or symbolic resources (for example, income, diplomas and self-perceived rank). Lacking or possessing such resources defines the type of social context to which individuals are exposed: lower social class contexts are often characterized by higher levels of constraints and uncertainty (for example, employment insecurity, scarce resources and social class prejudice), whereas higher social class contexts are characterized by more freedom and volition (for example, employment security, abundant resources and social class privilege). Repeated experiences in these different social contexts shape individuals’ psychological tendencies^6–9^. Because individuals from lower social classes live in more unstable and high-constraint contexts, they tend to be more oriented towards others and their environment (manifested in being vigilant to external influences and potential threats, seeing oneself as connected to others, and using community as a resource). By contrast, because individuals from higher social classes live in more stable and low-constraint contexts, they tend to be more oriented towards the self (manifested in being focused on internal states and potential rewards, seeing oneself as unique, and pursuing independence and self-sufficiency).

This central premise has generated a large number of studies documenting the effects of social class^10^. A literature review led us to identify 35 hypotheses that illustrate the pervasiveness of the psychological impact of social class on (1) the self, (2) relationships, (3) cognition, (4) emotion, (5) social behaviour and (6) decision-making (Table 1). First, regarding the self, the recurring hardships experienced by individuals from lower social classes manifest in a lower sense of control, perceived agency, and self-esteem than those observed in individuals from higher social classes^11,12^ (see also refs. ^13–15^). Because of the greater constraints and instability of their life contexts, individuals from lower social classes tend to construe their self as interdependent (emphasis on connection to others), whereas individuals from higher social classes construe their self as independent (emphasis on distinctiveness from others)^16,17^. Second, regarding relationships, individuals from lower social classes tend to develop small, close-knit and homogeneous social networks, which they rely on when facing adversity; by contrast, individuals from higher social classes have more dispersed and diverse networks and prioritize the use of personal economic resources when facing adversity^16,18^ (for work on social class and intergroup relations, see ref. ^19^). Third, regarding cognition, differences in terms of other/environment-oriented versus self-oriented psychological tendencies entail different cognitive styles and patterns of sense-making: individuals from lower social classes tend to process information as a global whole, anticipate more change in life trajectories (for example, a higher likelihood of going from being a millionaire to being bankrupt) and produce more contextual explanations of events (for example, failure and success are seen as beyond an individual’s control), whereas individuals from higher social classes process information in parts, anticipate more stability in life trajectories and produce more dispositional explanations of events^11,16^. Fourth, with respect to emotion, other/environment-oriented versus self-oriented psychological tendencies further suggest that individuals from lower social classes are more prone to other-oriented emotions such as compassion and love, whereas individuals from higher social classes are more prone to self-oriented emotions such as contentment and pride^20^ (for additional research, see refs. ^11,21^). Fifth, when it comes to social behaviour, these tendencies ultimately affect unethical and prosocial behaviours. For instance, individuals from lower social classes are more likely to lie when it benefits others (lying for a friend), whereas individuals from higher social classes are more likely to lie when it benefits themselves (lying in one’s own interest)^22^. Individuals from lower social classes also act more prosocially in private settings, whereas individuals from higher social classes act more prosocially in public settings (when their behaviour could reflect positively on the self)^23^ (see also ref. ^24^). Finally, concerning decision-making, owing to cumulative exposure to deprivation (sometimes since childhood), individuals from lower social classes tend to show greater risk aversion, increased preference for immediate rewards (rather than delayed rewards) and higher cognitive load when facing difficult financial decisions than individuals from higher social classes^25,26^ (for work on social class and moral decision-making, see ref. ^27^).

**Table 1:** List of the 35 hypothesized effects of social class derived from the 22 replicated studies

	Study ID	Journal, year and study number	Hypothesized effects	Sample		Quota or random	Main measure	*P*
				*N*	Power			
The self (9 hypotheses)	S1/2 (ref. ^11^)	*JPSP*, 2009, #1–2	H1. Social class $\stackrel{\!\!\!+}{\rightarrow}$ sense of control (×2 effects)	103/86	0.15, 0.53	No (USA)	Subjective	<0.05
	S3 (ref. ^12^)	*JPSP*, 2018, #1	H2–4. Social class $\stackrel{\!\!\!+}{\rightarrow}$ agency/persistence in goal striving/self-esteem/omnibus self-orientation measure	2,832	1.0, 1.0	✓ (USA/Japan)	Subjective Education	<0.001 <0.001
	S4 (ref. ^13^)	*PLoS ONE*, 2019	H5. Social class $\stackrel{\!\!\!+}{\rightarrow}$ narcissism	400	0.52, 0.98	No (China)	Subjective	<0.001
	S5/6 (ref. ^14^)	*PSPB*, 2014, #1a/b	H6. Social class $\stackrel{\!\!\!+}{\rightarrow}$ entitlement (×2, one “marginal”)	195/105	0.17, 0.80	No (USA)	Subjective	0.021
	S7 (ref. ^15^)	*JSP*, 2020, #1	H7. Social class × high/low system justification $\stackrel{\!\!\!+}{\rightarrow}$ entitlement	669	0.74, 0.96	No (China)	Occupation	<0.001
	S8a (ref. ^16^)	*PNAS*, 2010	H8. Social class $\stackrel{\!\!\!-}{\rightarrow}$ interdependent self-construal	235	0.33, 0.87	✓ (USA)	Education	NS
	S9 (ref. ^17^)	*JPSP*, 2007, #4b	H9. Social class $\stackrel{\!\!\!+}{\rightarrow}$ negative reactions to reduced individuation (×3, one NS)	801	0.81, 1.0	No (USA)	Education	<0.05
Relationships (7)	S8b (ref. ^16^)	*PNAS*, 2010	H10. Social class $\stackrel{\!\!\!-}{\rightarrow}$ individuals in one’s inner circle	235	0.33, 0.87	✓ (USA)	Education	<0.05
			H11. Social class $\stackrel{\!\!\!-}{\rightarrow}$ social support received					NS
	S10 (ref. ^18^)	*JPSP*, 2012, #1	H12. Social class × chaos/stability $\stackrel{\!\!\!-}{\rightarrow}$ communal orientation	76	0.14, 0.23	No (USA)	Income	0.01
	S11 (ref. ^18^)	*JPSP*, 2012, #4	H13. Social class × chaos/stability $\stackrel{\!\!\!+}{\rightarrow}$ obsession with money	134	0.21, 0.38	No (USA)	Subjective	<0.02
	S12 (ref. ^19^)	*JESP*, 2018, #2	H14. Education bias (that is, highly educated people rated higher)	448	0.56, 0.99	No (USA)	Education	<0.001
			H15. Social class $\stackrel{\!\!\!+}{\rightarrow}$ education bias					<0.001
			H16. Education bias = ethnic/national bias					NA
Cognition (4)	S8c (ref. ^16^)	*PNAS*, 2010	H17. Social class $\stackrel{\!\!\!-}{\rightarrow}$ thematic/holistic thinking style	235	0.33, 0.87	✓ (USA)	Education	0.08
	S8d (ref. ^16^)	*PNAS*, 2010	H18. Social class $\stackrel{\!\!\!-}{\rightarrow}$ anticipation of change	235	0.33, 0.87	✓ (USA)	Education	<0.01
	S13 (ref. ^11^)	*JPSP*, 2009, #3	H19. Social class $\stackrel{\!\!\!-}{\rightarrow}$ contextual explanations (×2)	444	0.56, 0.99	No (USA)	Subjective	<0.05
			H20. Social class $\stackrel{\!\!\!+}{\rightarrow}$ sense of control $\stackrel{\!\!\!-}{\rightarrow}$ contextual explanations (×3)					<0.05
Emotion (6)	S14 (ref. ^20^)	*Emotion*, 2018	H21–22. Social class $\stackrel{\!\!\!-}{\rightarrow}$ other-oriented positive emotions (×2)	1,519	0.97, 1.0	✓ (USA)	Income	≤0.002
			H23–24. Social class $\stackrel{\!\!\!+}{\rightarrow}$ self-oriented positive emotions (×2)					≤0.033
	E1 (ref. ^21^)	*Frontiers*, 2014, #2	H25. Social class × sharing × time $\stackrel{\!\!\!-}{\rightarrow}$ self-conscious negative emotions	103	0.17, 0.30	No (USA)	Composite	<0.05
	S15 (ref. ^11^)	*JPSP*, 2009, #4	H26. Social class $\stackrel{\!\!\!-}{\rightarrow}$ influence of contextual emotional information	125	0.20, 0.61	No (USA)	Subjective	<0.05
Behaviour (3)	E2 (ref. ^22^)	*JPSP*, 2015, #2	H27. Social class × self/other benefits $\stackrel{\!\!\!+}{\rightarrow}$ unethical behaviours	81	0.15, 0.25	No (Europe)	Income	<0.001
	E3 (ref. ^23^)	*SPPS*, 2016, #3	H28. Social class × private/public context $\stackrel{\!\!\!-}{\rightarrow}$ prosocial behaviour	363	0.48, 0.77	No (USA)	Other	0.001
	S16 (ref. ^24^)	*PNAS*, 2012, #5	H29. Social class $\stackrel{\!\!\!+}{\rightarrow}$ unethical behaviours	108	0.18, 0.55	No (USA)	Subjective	<0.02
Decision-making (6)	E4 (ref. ^25^)	*JESP*, 2018, #4	H30. Social class $\stackrel{\!\!\!+}{\rightarrow}$ preference for delayed reward	1,293	0.95, 1.0	No (USA)	Other	0.009
			H31. Social class $\stackrel{\!\!\!-}{\rightarrow}$ risk aversion					<0.001
			H32. Social class × mortality/control $\stackrel{\!\!\!{\varnothing}}{\rightarrow}$ delayed reward					NS
			H33. Social class × mortality/control $\stackrel{\!\!\!{\varnothing}}{\rightarrow}$ risk aversion					NS
	E5 (ref. ^26^)	*Science*, 2013, #1	H34. Social class × hard/ easy financial problems $\stackrel{\!\!\!+}{\rightarrow}$ performance	101	0.17, 0.30	No (USA)	Income	0.03
	S17 (ref. ^27^)	*JPSP*, 2013, #1	H35. Social class $\stackrel{\!\!\!+}{\rightarrow}$ utilitarian moral decision-making	277	0.38, 0.92	No (USA)	Other	<0.001

For each study, we report (1) sample size, (2) statistical power estimates to detect small (*r* = 0.10) and medium (*r* = 0.20) effects (because interaction effects are usually smaller than main effects^146^, our small/medium effect thresholds were halved for interactions; for the script, see the Open Science Framework (OSF) page for the project), (3) whether the sample was a quota-based or random sample, (4) the main measure of social class and (5) the critical *P* value(s).

In the study IDs, ‘S’ indicates a correlational study, whereas ‘E’ indicates an experimental study. In the hypotheses, $\stackrel{\!\!\!+}{\rightarrow}$ means ‘has a positive effect’; $\stackrel{\!\!\!-}{\rightarrow}$ means ‘has a negative effect’, $\stackrel{\!\!\!{\varnothing}}{\rightarrow}$ means ‘has a null effect’, = means ‘has an equivalence effect’ and NA means ‘not applicable’ (the effect was not tested in the original study). *JPSP*, *Journal of Personality and Social Psychology*; *PSPB*, *Personality and Social Psychology Bulletin*; *JSP*, *Journal of Social Psychology*; *PNAS*, *Proceedings of the National Academy of Sciences of the USA*; *Frontiers*, *Frontiers in Psychology*; *SPPS*, *Social Psychological and Personality Science*; NS, non-significant.

Together, the 35 hypotheses derived from the 22 studies referenced in the above paragraph embody a new line of research seeking to understand systematic social class differences that had, until recently, been mostly interpreted as random interindividual differences^28^. This body of research is highly influential. Although the articles testing these hypotheses have been published over the past decade, they have already garnered large numbers of citations (the median field-weighted citation impact was 3.4, meaning that the articles received 240% more citations than expected on the basis of the average number of citations of similar papers in the discipline over a three-year window)^29^. Moreover, most of these findings appear in studies published in prestigious, highly visible general or specialized journals such as *Science*, *Proceedings of the National Academy of Sciences* or the *Journal of Personality and Social Psychology*, and some of the findings have been used to inform policymakers (for example, see ref. ^30^).

However, this body of research is not immune to some of the core challenges faced by the social and behavioural sciences that have led to the “replication crisis”^31–33^: (1) small sample sizes, (2) non-diverse convenience samples and (3) measurement flexibility (either in the choice or in the reporting of the measure). First, the median sample size of the studies is relatively small (215 participants), with a mean statistical power between 0.42 and 0.70 (to detect small (corresponding to *r* = 0.10) and medium (*r* = 0.20) psychological effects, respectively^34^). Second, the vast majority of the studies (86%) use samples that are not distributed similarly to the underlying population in terms of key demographics, one-third use undergraduates and most participants (89%) are from the USA. Third, and finally, the measure of social class varies greatly^35^ between studies, without—in most cases—any justification provided: the measure is based on participants’ level of education (approximately one-quarter of the studies), income (around one-quarter of the studies) and/or subjective social class (~1/2 of the studies). Each of these three challenges increases the probability of type I errors and threatens generalizability^36–38^. In fact, about half of the critical *P* values from the 22 studies are above 0.01, when the majority of the *P* values should be below 0.01 if all effects are true (*P* values between 0.01 and 0.05 are indicative of lower odds of replication, as the distribution of *P* values is right-skewed for both adequately and insufficiently powered studies)^39,40^.

To address these three challenges, we carried out a theoretically comprehensive replication of the 22 studies^11–27^ described above using (1) samples that yield high power, (2) samples that are either distributed similarly to the national population in terms of key demographics (USA, France and India) or representative of the national population (Switzerland), and (3) a preregistered method and analytical strategy. Specifically, we replicated 17 correlational and 5 experimental studies selected on the basis of criteria related to theoretical relevance, domain coverage and feasibility (for detailed information about the study selection process, see ‘Design’ in Methods). Table 1 lists the hypothesized effects derived from the studies to be replicated, and Supplementary Table 1 lists all the deviations from the original studies (including lab versus online setting, even if—on the basis of the results from Many Labs 2 (ref. ^41^)—we see no reason to expect this deviation to influence the findings). A detailed description of the preregistered hypotheses, materials and analytical strategies is provided in the Design Table of the Stage 1 protocol and in the preregistration (https://osf.io/3tjzs/).

We recruited a total of *N* = 33,536 participants from four countries—namely, three samples that are matched to the underlying population in terms of key demographic features (later referred to as quota-based samples) of *N* = 9,019 US residents, *N* = 9,160 French residents and *N* = 9,556 Indian residents and one random representative sample of *N* = 5,801 Swiss residents. The countries were selected on the basis of both practical and cultural reasons (for detailed information about the selection of countries, see ‘Sampling plan’ in Methods). Importantly, four national samples provided more cultural diversity than is usually found in extant research but are not sufficient to estimate the worldwide generalizability of the findings.

The participants completed a 20-min online questionnaire that included a random subset of 2/3 of the outcome variables used in the original correlational studies and—at most—one experiment. The questionnaire also included nine commonly used measures of social class: education, income, occupation, subjective socio-economic status (SES), childhood subjective SES, childhood SES, social class self-categorization, financial scarcity and sense of power (for detailed information, see ‘Design’ in Methods). Supplementary Tables 4 and 5 present the sample size, reliability and descriptive statistics by country for each social class predictor and each study variable, respectively. For each country, the statistical power to detect a small-sized effect of social class (*η*_p_^2^ = 0.01) on a given outcome variable was above 0.95 in most cases, although it was lower for the experiments conducted in Switzerland and/or when excluding rushers (for detailed information, see ‘Sampling plan’ in Methods).

The primary purpose of our work was to test the 35 hypotheses derived from the 22 studies using the same analytical strategy used in the original studies (confirmatory preregistered analysis). To ensure the quality and fairness of the replication project, the hypotheses, method and analytical strategy were extracted from the original publications and compiled in a preregistration document; this document was then submitted to the original authors for review and amended according to their feedback. A replication was considered successful if we observed an effect in the same direction as in the original study with an *α* of 0.05 (for detailed information, see ‘Analysis plan’ in Methods). With samples as large as ours, even practically insignificant effects can be statistically significant^42^. However, large sample sizes allow one to obtain reliable estimates of effect size, and we used these estimates (along with their confidence intervals) to further describe each replicated effect.

The secondary purpose of our work was to systematically compare the predictive strength of the nine measures of social class when testing the hypotheses (exploratory preregistered analysis). Although social class measures are often used in a flexible way in extant research, theory suggests that these measures should not always be considered interchangeable. Social class contexts involve complex combinations of different types of objective and self-perceived economic, cultural and symbolic resources. These dimensions are related but can be independent (for example, income and education are positively correlated, though one can have a high income level and a low education level). They are associated with different experiences (for example, economic abundance or familiarity with higher social class practices) and could shape different psychological patterns^4^. Investigating whether and how the different dimensions of social class contexts foster different psychological and behavioural tendencies could facilitate the refinement of theoretical predictions and the development of new insights.

The tertiary purpose of our work was to test three relevant potential moderators of the main effects to be replicated (confirmatory preregistered analysis). First, we tested a moderator located at the intra-individual level: social class identification. Because social class identification is defined as the degree to which an individual assigns subjective value to their social class^43^, it should logically increase the strength of the effects of social class. Second, we tested a moderator located at the ideological level: system-justification beliefs. However, our prediction was tentative. On the one hand, because believing that society is fair implies that individuals from lower social classes deserve their social status (threatening their ego), system-justification beliefs could increase the strength of most effects of social class (on the self, relationships, emotions and behaviours)^15^. On the other hand, system-justification beliefs can serve a palliative function for disadvantaged groups (preserving their ego), which could make this prediction inaccurate^44,45^. Third, we tested a moderator located at the structural level: local income inequality. Because a higher level of income inequality increases the salience of social class stratification^46,47^, it should increase the strength of the effects of social class. Investigating these moderators should help refine the theoretical framework of the psychology of social class by specifying the boundary conditions of these effects.

## Results

Deviations from the preregistered study protocol are reported in detail in the Supplementary Information (‘List of deviations’, pp. 42–44) and summarized in a dedicated section in the Methods.

### Primary analysis: replications

**Fig. 1:**
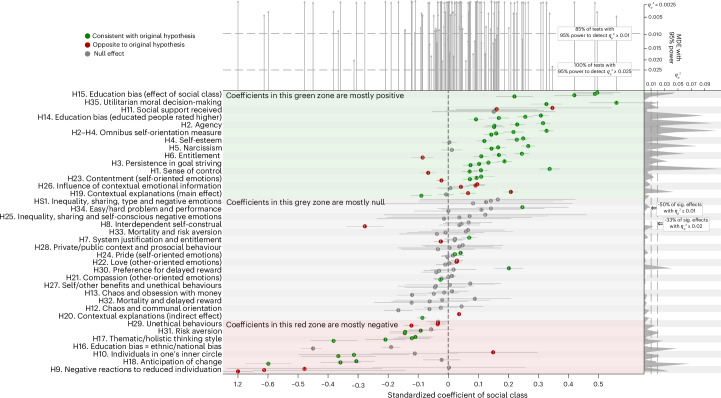
Primary analysis. Focal estimates and conclusions for each hypothesis and each country. The central panel shows the *β* (circles) and 95% CI (error bars) for each hypothesis and country (U, USA; F, France; S, Switzerland; I, India). The upper panel shows the minimum detectable effect size (MDE) with 95% power, vertically aligned with each estimate, with longer arrows indicating greater statistical power (85% of the tests had 95% power detect an effect with *η*_p_^2^ ≥ 0.01). The right panel shows the *η*_p_^2^, horizontally aligned with each estimate, with taller shaded areas indicating a greater percentage of variance explained (50% of the effects have *η*_p_^2^ ≥ 0.01). This figure presents the same focal test per hypothesis as reported in Table 1; the note for Table 1 also applies here. Supplementary Table 9 provides the exact values for *η*_p_^2^ and MDE used in the upper and right panels. Supplementary Table 5 provides the analytical sample size (*N*) for each hypothesis and country. The formulas for calculating the MDEs are provided in the Supplementary Information (‘Calculation of the MDE’, p. 33).

In the US sample, where most of the original studies were conducted, the overall replication rate was 56%. In the French, Swiss and Indian samples, the replication rates were 50%, 45% and 41%, respectively. Supplementary Table 6 presents detailed information regarding the calculation of replication rates. These numbers are comparable to those from replication initiatives in psychology, where approximately half of the effects tend to be successfully replicated^41,48,49^, and they exceed the rate observed in a recent replication initiative focused on the psychology of scarcity (that is, 22%)^50^. Below, we provide a narrative summary, organized by outcome type, that outlines the conclusions from the tests of the 35 hypotheses in the four samples. Table 2 presents one key statistical result and the overarching conclusion for each country and hypothesis, while Fig. 1 provides a graphical overview of these analyses. The Supplementary Information (‘Detailed report’, pp. 20–32) presents the details of all replication analyses undertaken.

**Table 2:** Primary analysis: focal results and conclusion for each hypothesis

	ID	Hypothesis	No.	USA		France		Switzerland		India	
				*β*	95% CI	*β*	95% CI	*β*	95% CI	*β*	95% CI
The self (9 hypotheses)	H1	Social class $\stackrel{\!\!\!+}{\rightarrow}$ sense of control	3/4	0.07***	(0.05, 0.10)^C^	0.11***	(0.08, 0.13)^C^	0.34***	(0.30, 0.37)^C^	−0.07***	(−0.09, −0.04)^R^
	H2	Social class $\stackrel{\!\!\!+}{\rightarrow}$ agency	4/4	0.32***	(0.29, 0.34)^C^	0.15***	(0.13, 0.18)^C^	0.15***	(0.12, 0.19)^C^	0.23***	(0.21, 0.25)^C^
	H3	Social class $\stackrel{\!\!\!+}{\rightarrow}$ persistence in goal striving	4/4	0.19***	(0.16, 0.21)^C^	0.10***	(0.08, 0.13)^C^	0.13***	(0.10, 0.17)^C^	0.07***	(0.05, 0.10)^C^
	H4	Social class $\stackrel{\!\!\!+}{\rightarrow}$ self-esteem	3/4	0.25***	(0.23, 0.27)^C^	0.12***	(0.10, 0.15)^C^	0.23***	(0.19, 0.26)^C^	0.00	(−0.02, 0.03)^N¶^
	H2–4	Social class $\stackrel{\!\!\!+}{\rightarrow}$ omnibus self-orientation measure	4/4	0.33***	(0.30, 0.35)^C^	0.16***	(0.14, 0.18)^C^	0.22***	(0.18, 0.25)^C^	0.14***	(0.12, 0.17)^C^
	H5	Social class $\stackrel{\!\!\!+}{\rightarrow}$ narcissism	3/4	0.27***	(0.24, 0.29)^C^	0.17***	(0.14, 0.19)^C^	0.01	(−0.02, 0.05)^N^	0.14***	(0.12, 0.17)^C^
	H6	Social class $\stackrel{\!\!\!+}{\rightarrow}$ entitlement	3/4	0.25***	(0.22, 0.27)^C^	0.11***	(0.08, 0.14)^C^	−0.09***	(−0.12, −0.05)^R^	0.17***	(0.14, 0.19)^C^
	H7	Social class × system-justification $\stackrel{\!\!\!+}{\rightarrow}$ entitlement	1/4	0.07***	(0.05, 0.09)^C^	0.02^†^	(0.00, 0.05)^N^	0.02	(−0.01, 0.06)^N^	−0.02*	(−0.05, 0.00)^R^
	H8	Social class $\stackrel{\!\!\!-}{\rightarrow}$ interdependent self-construal^A^	1/4	0.04	(−0.02, 0.11)^N^	0.04	(−0.03, 0.10)^N^	0.07^†^	(−0.01, 0.15)^N¶^	−0.28***	(−0.34, −0.22)^C^
	H9	Social class $\stackrel{\!\!\!+}{\rightarrow}$ negative reactions to reduced individuation^B^	0/4	−0.48***	(−0.74, −0.22)^R^	0.00	(−0.25, 0.26)^N^	−0.61*	(−1.1, −0.14)^R^	−1.2***	(−1.4, −1.0)^R^
Relationships (7)	H10	Social class $\stackrel{\!\!\!-}{\rightarrow}$ individuals in one’s inner circle	2/4	−0.37***	(−0.49, −0.24)^C^	−0.11	(−0.26, 0.03)^N^	−0.31***	(−0.50, −0.13)^C^	0.15*	(0.00, 0.30)^R^
	H11	Social class $\stackrel{\!\!\!-}{\rightarrow}$ social support received^A^	0/4	0.16*	(0.03, 0.30)^R^	0.16^†^	(−0.01, 0.32)^N^	0.15	(−0.05, 0.36)^N^	0.35***	(0.15, 0.55)^R^
	H12	Social class × chaos/stability $\stackrel{\!\!\!-}{\rightarrow}$ communal orientation	0/4	−0.02	(−0.08, 0.03)^N^	−0.06*	(−0.11, −0.01)^N^	0.04	(−0.03, 0.12)^N^	−0.17***	(−0.21, −0.12)^N^
	H13	Social class × chaos/stability $\stackrel{\!\!\!+}{\rightarrow}$ obsession with money	0/4	−0.01	(−0.06, 0.04)^N^	0.01	(−0.04, 0.06)^N^	−0.05	(−0.13, 0.03)^N^	−0.12***	(−0.17, −0.07)^N^
	H14	Education bias (highly educated people rated higher)	4/4	0.26***	(0.23, 0.28)^C^	0.09***	(0.07, 0.12)^C^	0.17***	(0.13, 0.20)^C^	0.31***	(0.28, 0.33)^C^
	H15	Social class $\stackrel{\!\!\!+}{\rightarrow}$ education bias	4/4	0.49***	(0.43, 0.55)^C^	0.42***	(0.35, 0.49)^C^	0.50***	(0.42, 0.57)^C^	0.22***	(0.16, 0.28)^C^
	H16	Education bias = ethnic/national bias^C^	0/4	−0.19***	(−0.22, −0.16)^N^	NA	Missing	NA	Missing	−0.45***	(−0.49, −0.42)^N^
Cognition (4)	H17	Social class $\stackrel{\!\!\!-}{\rightarrow}$ thematic/holistic thinking style	3/4	−0.12***	(−0.18, −0.06)^C^	−0.21***	(−0.28, −0.14)^C^	−0.38***	(−0.46, −0.30)^C^	−0.11***	(−0.17, −0.05)^C^
	H18	Social class $\stackrel{\!\!\!-}{\rightarrow}$ anticipation of change	3/4	−0.02	(−0.08, 0.04)^N^	−0.36***	(−0.43, −0.29)^C^	−0.60***	(−0.68, −0.52)^C^	−0.31***	(−0.37, −0.24)^C^
	H19	Social class $\stackrel{\!\!\!-}{\rightarrow}$ contextual explanations	1/4	0.07***	(0.04, 0.09)^R^	−0.01	(−0.03, 0.02)^N^	−0.09***	(−0.12, −0.05)^C^	0.21***	(0.18, 0.23)^R^
	H20	Social class $\stackrel{\!\!\!+}{\rightarrow}$ sense of control $\stackrel{\!\!\!-}{\rightarrow}$ contextual explanations	1/1	−0.02***	(−0.03, −0.01)^×^	−0.02***	(−0.03, −0.02)^×^	−0.09***	(−0.10, −0.07)^C^	0.04***	(0.03, 0.04)^×^
Emotion (7)	H21	Social class $\stackrel{\!\!\!-}{\rightarrow}$ compassion (other-oriented emotion)	1/4	−0.03**	(−0.05, −0.01)^C^	0.00	(−0.02, 0.02)^N^	−0.02	(−0.05, 0.00)^N^	0.01^†^	(0.00, 0.03)^N^
	H22	Social class $\stackrel{\!\!\!-}{\rightarrow}$ love (other-oriented emotion)	0/4	0.03**	(0.01, 0.04)^R^	0.03**	(0.01, 0.05)^R^	−0.01	(−0.04, 0.02)^N^	0.00	(−0.01, 0.02)^N^
	H23	Social class $\stackrel{\!\!\!+}{\rightarrow}$ contentment (self-oriented emotion)	3/4	0.09***	(0.07, 0.12)^C^	0.07***	(0.05, 0.10)^C^	0.11***	(0.06, 0.15)^C^	−0.02*	(−0.04, −0.01)^R^
	H24	Social class $\stackrel{\!\!\!+}{\rightarrow}$ pride (self-oriented emotion)	1/4	0.02*	(0.00, 0.04)^C^	0.00	(−0.02, 0.02)^N^	0.02	(−0.02, 0.05)^N¶^	0.04***	(0.03, 0.05)^C^
	H25	Social class × sharing × time $\stackrel{\!\!\!-}{\rightarrow}$ self-conscious emotions	0/4	0.04	(−0.20, 0.28)^N^	0.07	(−0.19, 0.33)^N^	0.12	(−0.21, 0.46)^N^	−0.01	(−0.24, 0.21)^N^
	H25′	Social class × sharing × time × type $\stackrel{\!\!\!-}{\rightarrow}$ negative emotions^A^	0/4	0.14	(−0.10, 0.38)^N^	0.17	(−0.09, 0.42)^N^	0.08	(−0.25, 0.41)^N^	0.13	(−0.10, 0.35)^N^
	H26	Social class $\stackrel{\!\!\!-}{\rightarrow}$ influence of contextual information	0/4	0.09***	(0.06, 0.12)^R^	0.04**	(0.01, 0.07)^R^	0.01	(−0.03, 0.05)^N^	0.10***	(0.07, 0.12)^R^
Behaviour (3)	H27	Social class × self/other benefits $\stackrel{\!\!\!+}{\rightarrow}$ unethical behaviours	0/4	−0.04	(−0.15, 0.06)^N^	0.07	(−0.03, 0.18)^N^	0.01	(−0.14, 0.15)^N^	−0.04	(−0.17, 0.08)^N^
	H28	Social class × private/public $\stackrel{\!\!\!-}{\rightarrow}$ prosocial behaviour	0/4	−0.03	(−0.16, 0.09)^N^	0.05	(−0.09, 0.18)^N^	0.00	(−0.17, 0.18)^N^	0.04	(−0.08, 0.15)^N^
	H29	Social class $\stackrel{\!\!\!+}{\rightarrow}$ unethical behaviours	0/4	−0.03**	(−0.06, −0.01)^R^	−0.03**	(−0.06, −0.01)^R^	−0.03^†^	(−0.07, 0.00)^N^	−0.12***	(−0.15, −0.10)^R^
Decision-making (6)	H30	Social class $\stackrel{\!\!\!+}{\rightarrow}$ preference for delayed reward	1/4	−0.03	(−0.08, 0.02)^N^	−0.04	(−0.09, 0.01)^N^	0.02	(−0.06, 0.09)^N^	0.20***	(0.15, 0.25)^C^
	H31	Social class $\stackrel{\!\!\!-}{\rightarrow}$ risk aversion	3/4	−0.14***	(−0.19, −0.09)^C^	−0.14***	(−0.20, −0.09)^C^	−0.06	(−0.13, 0.02)^N^	−0.09***	(−0.14, −0.04)^C^
	H32	Social class × mortality/control $\stackrel{\!\!\!{\varnothing}}{\rightarrow}$ delayed reward^D^	0/4	−0.12	(−0.25, 0.00)^N^	−0.06	(−0.19, 0.07)^N^	−0.02	(−0.18, 0.14)^N^	0.09	(−0.03, 0.21)^N^
	H33	Social class × mortality/control $\stackrel{\!\!\!{\varnothing}}{\rightarrow}$ risk aversion^D^	0/4	−0.04	(−0.16, 0.09)^N^	−0.01	(−0.14, 0.12)^C^	0.06	(−0.10, 0.22)^N^	0.07	(−0.06, 0.19)^N^
	H34	Social class × hard/easy problems $\stackrel{\!\!\!+}{\rightarrow}$ performance^D^	1/4	0.11^†^	(−0.02, 0.24)^N^	0.25**	(0.09, 0.40)^C^	0.14	(−0.04, 0.32)^N^	0.00	(−0.11, 0.11)^N^
	H35	Social class $\stackrel{\!\!\!+}{\rightarrow}$ utilitarian moral decision-making	2/2	0.56***	(0.47, 0.65)^C^	NA	Missing	NA	Missing	0.33***	(0.27, 0.38)^C^
	Percentage of fully replicated effects			46.9%		36.7%		41.9%		31.3%	
	Percentage of partially replicated effects			9.4%		13.3%		3.2%		9.4%	
	Percentage of total replication			56.3%		50.0%		45.1%		40.7%	

C, consistent; R, reversed; N, non-consistent.

For clarity, we report one focal test per hypothesis. We used the same control variables used in the original study. When the original main analysis reported multiple tests for a hypothesis, we prioritized the test that excluded covariates (to maximize power) and used the predictor from the primary analysis (that is, subjective SES for H2–4, income for H27, or education for H25 and H28). This choice did not alter the conclusions drawn from the analyses. Supplementary Table 7 presents the focal statistical results for all model specifications used in the original main analysis. Supplementary Table 8 shows whether non-significant estimates are equivalent to zero, showing that non-significant estimates for shorter tasks or scales (where the sample size was larger) are generally equivalent to zero, while the tests for longer tasks or experiments (where the sample size was smaller) are generally inconclusive. All predictors and outcomes were standardized, which means that coefficients can roughly be interpreted as correlations, with three exceptions: (1) when social class is operationalized using education—in this case, the coefficient captures the difference between the lowest and highest education group; (2) when an interaction is tested—in this case, the coding for dichotomous variables is aligned with the direction of the hypothesized effect (for example, for H12–13, chaos/stability means that ‘chaos’ was coded as +0.5 and ‘stability’ as −0.5); and (3) when the outcome is binary—in this case, logistic rather than linear regression was used, and log-odds are reported (that is, for H9 and H35).

In the hypotheses, $\stackrel{\!\!\!+}{\rightarrow}$ means ‘has a positive effect’, $\stackrel{\!\!\!-}{\rightarrow}$ means ‘ has a negative effect’, $\stackrel{\!\!\!{\varnothing}}{\rightarrow}$ means ‘has a null effect’ and = means ‘has an equivalent effect’. The ‘No.’ column indicates the number of countries where the results were consistent with the original hypothesis. ‘Percentage of fully replicated effects’ refers to the proportion of hypothesized effects that were consistently replicated in most of the original specifications, whereas ‘Percentage of partially replicated effects’ refers to the proportion of hypothesized effects that were not always replicated across the original specifications. ‘Missing’ indicates a shortcoming in the questionnaire where a focal variable was not assessed, and ‘NA’ means ‘not applicable’ (as the data was missing). The prime symbol (′) indicates that the test was not performed in the original study, but we considered it a more appropriate test of the hypothesis. The cross symbol (×) indicates that the indirect effect is reported for transparency but is not interpreted, as the total effect is either inconsistent with the hypothesis or reversed. The pilcrow (¶) indicates tests where the outcome variable has a Cronbach’s *α* of less than 0.60, meaning that the results should be interpreted with caution.

^A^H8, H11 and H25′ were excluded from the calculation of replication rates because the original authors did not report significant results (for the first two) or did not carry out the tests performed (for the last one).

^B^For H9, the original study tested the hypothesis by running three different regressions on three different items measuring the same construct. Here, for the sake of brevity, we report the mean effect across these analyses. The conclusions from the individual analyses are identical.

^C^For H16, the focal test is an equivalence test, meaning that *β* represents the standardized difference between education and ethnic/national bias, and the confidence interval (CI) does not represent a 95% but a 90% CI.

^D^For H32, H33 and H34, since the experiments used lengthy inductions, we chose to report the focal test excluding rushers (that is, participants who spent less than half the median time on the induction); we urge caution in interpreting these findings, as the sample size provided 95% power only to detect an effect size *η*_p_^2^ ≥ 0.025 rather than the 0.01 initially planned.

***P < 0.001; **P < 0.01; *P < 0.05; ^†^*P* < 0.10. (Exact *P* values are reported in Supplementary Table 7.)

#### The self

The results pertaining to the effects of social class on the self were consistent with seven of the nine original hypotheses in at least two countries. First, in line with the core theoretical tenet that individuals from higher social classes are more inclined to believe that their lives are determined by their own actions, a higher social class was associated with a higher sense of control (H1), increased perceived agency (H2) and more persistent goal striving (H3). The only exception was India, where a higher social class unexpectedly predicted a lower sense of control. Second, illustrating the fact that a higher social class fosters not only a positive self-image but also a self-aggrandizing view of the self, we observed that social class was generally positively associated with self-esteem (H4), narcissism (H5) and entitlement (H6). An exception was Switzerland, where a higher social class unexpectedly predicted a lower level of entitlement. Additionally, outside of the USA, system-justification beliefs did not strengthen the relationship between social class and entitlement (H7). Third, and finally, our data did not support the central idea that individuals from higher social classes endorse and value independence and uniqueness. Specifically, we did not find that individuals from higher social classes hold a more independent self-construal than individuals from lower social classes, except in India (H8). Surprisingly, we found that they reacted more positively, rather than more negatively, when experiencing reduced individuation (H9) in the US, Swiss and Indian samples.

##### Summary

Consistent with earlier findings, higher social class is generally associated with a greater sense of control, agency, persistence, self-esteem, narcissism and entitlement. Inconsistent with earlier findings, higher social class is generally associated with a more positive (rather than negative) reaction to reduced individuation, while there is little evidence for social class differences in self-construal or for an interaction effect between social class and system-justification beliefs on entitlement.

#### Relationships

The results pertaining to the effects of social class on relationships were consistent with three of the seven original hypotheses in at least two countries. First, our results provided mixed support for the central premise that individuals from lower social classes enjoy more tight-knit, intimate and supportive relationships than individuals from higher social classes. On the one hand, we did observe that individuals from lower social classes generally included a higher proportion of their network members in their inner circle (H10), although the effect was reversed in India. On the other hand, we did not observe that individuals from lower social classes received more social support from their network (H11); in fact, they reported experiencing more social annoyance in both the USA and India. Second, our results did not support the hypothesis that individuals from lower social classes prioritize relationships to cope with future financial instability (H12) or that those from higher social classes prioritize money (H13). Instead, in France and India, it was individuals from higher social classes who demonstrated a more communal orientation when expecting financial stability. Third, regarding intergroup relations, participants from all four countries consistently showed a bias against lower-educated individuals (H14), particularly when they themselves belonged to a higher social class (H15). However, in the two countries where the comparison was possible, our analysis indicated that the size of the education bias was not equivalent to, but smaller than, that of the ethnicity bias (H16).

##### Summary

Consistent with earlier findings, higher social class is generally associated with a looser social network and greater bias against individuals with lower education. Inconsistent with earlier findings, higher social class is generally associated with greater (rather than lesser) social support and with a greater reliance on friendship in times of financial stability (rather than on money in times of financial instability). Finally, education bias is weaker, rather than similar to, ethnic bias.

#### Cognition

The results pertaining to the effects of social class on cognition were consistent with two of the four original hypotheses in at least two countries. First, our findings were mostly congruent with the theoretical claim that individuals from higher social classes exhibit a more analytical cognitive style, while individuals from lower social classes exhibit a more holistic cognitive style. We observed that individuals from higher social classes systematically favoured taxonomic (indicative of analytical thinking) rather than thematic (indicative of holistic thinking) categorization (H17). Additionally, individuals from higher social classes saw life trajectories as more linear and less susceptible to change (also indicative of analytical thinking) (H18), except in the USA. Second, our findings were mostly incongruent with the claim that social class predicts different attributional patterns. Only in Switzerland did we observe that individuals from lower social classes were more likely to explain life events (for example, being laid off) using contextual rather than dispositional causes (H19), a relationship that—as in the original study—was mediated by a lower level of sense of control (H20). In France, we observed no such relationship, while in the USA and India, individuals from lower social classes were in fact more likely to offer dispositional explanations of life events.

##### Summary

Consistent with earlier findings, higher social class is generally associated with more taxonomic categorization and less anticipation of change in life trajectories. Inconsistent with earlier findings, the association between social class and attribution patterns varies across countries.

#### Emotions

The results pertaining to the effects of social class on emotions were consistent with two of the six original hypotheses in at least two countries. First, our data offered mixed support for the key proposition that social class differences in terms of self- and other-orientation manifest in different experiences of positive emotions. On the one hand, there was some evidence that individuals from higher social classes experience more self-oriented positive emotions: they reported higher levels of both contentment (H23) and pride (H24). The sole exception was India, where they reported feeling less, not more, contentment. On the other hand, there was very little evidence that individuals from lower social classes experience more other-oriented positive emotions: only in the USA did they report experiencing greater compassion (H21), and generally, they reported experiencing less, not more, love (H22). Second, regarding negative emotions, our data did not substantiate the hypothesis that individuals from lower social classes experience more negative self-conscious emotions when a stranger shares fewer resources with them in a dictator game (H25). Third, contrary to the hypothesis that individuals from lower social classes are more attentive to contextual information (H26), we observed that individuals from higher social classes were more influenced by contextual information when rating emotional facial expressions.

##### Summary

Consistent with earlier findings, higher social class is generally associated with more contentment and more pride. Inconsistent with earlier findings, higher social class is generally associated with more (rather than less) love and more (rather than less) sensitivity to contextual emotional cues, while there is little evidence for social class differences in compassion or for an interaction effect between social class and negative sharing interactions on self-conscious emotions.

#### Behaviour

The results pertaining to the effects of social class on behaviour were not consistent with the three original hypotheses. Generally, our data contradicted the idea that individuals from higher social classes are more self-oriented and have fewer concerns for others, and therefore engage in more unethical behaviour. We found no evidence that individuals from higher social classes were more likely to use deception in a hypothetical negotiation task (H29). In most samples, they were in fact less, not more, likely to act unethically in this task. Additionally, our findings indicated that the relationship between social class and unethical behaviour is not moderated by the context of behaviour (that is, cheating for oneself versus for others or being generous in public versus private). We found no evidence that individuals from higher social classes engage in more unethical behaviour when it benefits themselves rather than others (H27). Similarly, we found no evidence that individuals from higher social classes donate fewer tickets in a dictator game in private contexts (where generosity is driven by intrinsic concerns for others) than in public contexts (where generosity is driven by strategic concerns of positive self-image) (H28).

##### Summary

None of the effects are consistent with earlier findings. Inconsistent with earlier findings, higher social class is generally associated with less (rather than more) unethical behaviours, while there is little evidence for an interaction effect between social class and other- versus self-beneficial behaviour on unethical behaviour, or for an interaction effect between social class and private versus public context on generosity.

#### Decision-making

The results pertaining to the effects of social class on decision-making were consistent with two of the six original hypotheses in at least two countries. First, our results offered partial support for the theoretical proposition that past experiences of hardship lead individuals from lower social classes to avoid financial risks and prefer smaller immediate gains to larger future gains. On the one hand, consistent with the original hypothesis, individuals from lower social classes were generally more risk-averse than individuals from higher social classes (H30). On the other hand, except in India, individuals from lower social classes did not demonstrate a stronger preference for immediate pay-offs over delayed ones (H31). However, the results of the equivalence tests were inconclusive, preventing us from determining whether there was indeed no interaction effect between social class and the salience of a dangerous world on financial risk aversion (H32) or preference for delayed reward, as hypothesized (H33). Second, only in France did our findings provide support for the idea that asking individuals from lower social classes to think about hard rather than easy financial decisions results in an increase in cognitive load negatively affecting their performance on Raven’s Matrices (H34). Third, and finally, as expected, individuals from higher social classes favoured utilitarian over deontological decisions when faced with the trolley dilemma (H35).

##### Summary

Consistent with earlier findings, higher social class is generally associated with less risk aversion and more utilitarian moral decisions. Inconsistent with earlier findings, there is little evidence for social class differences in preference for delayed reward or for an interaction effect between social class and exposure to easy versus difficult financial problems on cognitive load.

### Secondary analysis: comparison of social class measures

#### Direction of the estimates

**Fig. 2:**
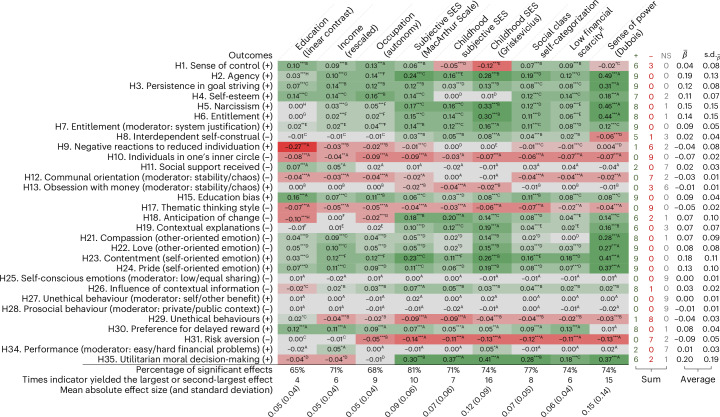
Secondary analysis. Comparison of the nine social class indicators for each relevant hypothesis in the pooled sample. This figure presents the standardized coefficient estimates from fixed-effects regressions (with country fixed effects) testing the link between each social class indicator and outcome (excluding the original control variables for comparability). The sign of the hypothesized effect of social class is indicated in parentheses after the outcome in the first column. Five hypotheses were excluded from the analysis because social class was not the predictor (H14 and H16), they pertained to an indirect effect (H20) or they predicted a null moderation (H32–H33). Darker green cases indicate more positive estimates, darker red cases indicate more negative estimates and grey cases indicate non-significant estimates (after correction for multiple comparisons). Education is the planned contrast (−0.5, lowest group; 0, middle; 0.5, highest). Income was log-transformed and rescaled within countries to account for differences in monetary units (that is, 1 unit = 1 s.d.(log(income))_within_). Financial scarcity was reverse-coded, with a higher score indicating higher social class. Objective indicators are education, income, occupation and scarcity. $\overline{\beta }$ indicates the average standardized coefficient across social class indicators. ***P < 0.001; **P < 0.01; *P < 0.05 (significance levels were adjusted for multiple testing for each hypothesis using a sequential Bonferroni procedure; the exact *P* values can be found in Supplementary Table 11).

We re-examined each hypothesis using the nine social class indicators in the data pooled from the four samples. A comparison of the indicators suggests that the operationalization of social class had minimal influence on the direction and significance of the standardized estimates. Figure 2 illustrates this by showing when the estimate is positive (green), negative (red) or non-significant (grey), along with a tally of these estimates by hypothesis on the right-hand side. For each hypothesis, at least five of the nine social class indicators produced similar results in terms of both the sign of estimates and statistical significance. For 3/4 of the hypotheses, seven or more of the nine indicators yielded similar conclusions. This suggests that researchers studying the influence of social class on the self, relationships, cognition, emotions, decision-making or behaviours are likely to reach similar conclusions, whether they use objective or subjective measures of current or childhood social class. Despite the overall uniformity in the findings, two cases stand out as exceptions: both measures of childhood SES were negatively, rather than positively, associated with sense of control, and both income and education were negatively, rather than positively, associated with utilitarian moral decision-making.

#### Magnitude of the estimates

However, the operationalization of social class did influence the magnitude of the standardized estimates. Figure 2 illustrates this by assigning different superscript letters to adjacent estimates that were significantly different and by showing the standard deviation of the estimates on the right-hand side. For over 70% of the hypotheses, at least one of the social class indicators yielded a larger estimate than the others. The variation between indicators was especially pronounced for hypotheses concerning the self or self-reported emotions, and less so for those related to relationships or decision-making. For the 21 hypotheses where social class indicators yielded different estimate sizes, childhood SES was most often the strongest or second-strongest indicator (16 times), followed by sense of power (15 times) and subjective SES (10 times). This result is perhaps not surprising given that these indicators capture subjective perceptions and experiences of social class, making them more proximal to psychological tendencies. The last row of Fig. 2 depicts the mean absolute effect size for each indicator. It shows slightly larger estimates (0.07 < *β* < 0.15) for indicators focused on perceived or experienced social standing than for those focused on objective life circumstances (0.05 < *β* < 0.06).

#### Connection with the replications

We generally found that the conclusions from the replication analyses were unaffected by how social class was operationalized. Broadly speaking, hypothesized effects that replicated or failed to replicate in the primary analysis exhibited similar patterns across most indicators in the secondary analysis. However, there were exceptions. First, three hypotheses that were not supported in the primary analysis received support in the pooled sample. Social class did predict a preference for immediate pay-offs over delayed ones for 8 out of 9 of the indicators (H30), system-justification beliefs did strengthen the link between social class and entitlement for all indicators (H7) and thinking about hard (versus easy) problems did cause individuals with lower income and lower childhood SES to perform worse on Raven’s Matrices (H34). Second, three hypothesized effects that were mostly null in the primary analysis turned out to be in the opposite direction in the pooled sample. A higher social class predicted a more interdependent self-construal, rather than a more independent self-construal, for 5 out of 9 of the indicators (H8), as well as higher levels of compassion, rather than lower levels, for 8 out of 9 of the indicators (H21). Moreover, social class interacted with financial stability in predicting communal orientation for 7 out of 9 of the indicators (H12). However, simple slope analyses revealed patterns inconsistent with the hypothesis, as instability did not increase communality among individuals from lower social classes but instead lowered it among those from higher social classes. Third, while the effect of social class, as measured by education, on anticipation of change was mostly negative in the primary analysis, it was positive (that is, reversed) for 5 out of 9 of the indicators (H18).

#### Summary

While the direction of the estimates and the conclusions of the replication do not depend much on which social class indicator is used (with some exceptions), the magnitude of the estimates is smaller when using objective indicators.

### Tertiary analysis: moderation

**Fig. 3:**
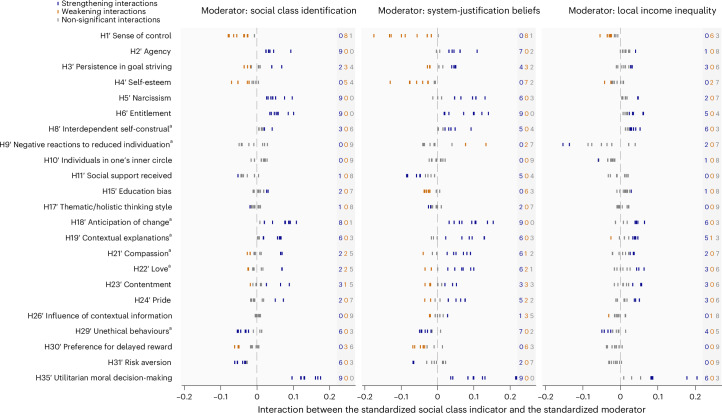
Tertiary analysis. Interaction between each social class indicator and moderator in the pooled sample. Each panel of this figure displays coefficient estimates from fixed-effects regression models (with country fixed effects) testing interaction effects between a specific social class indicator and social class identification (left), system-justification beliefs (middle) and local income inequality (right) on a given outcome (excluding the original control variables for comparability). Counts of significant strengthening interactions, significant weakening interactions, and non-significant interactions appear on the right of each panel, with the largest count in bold. H12′–H14′, H25′, H16′, H27′–H28′ and H32′–H33′ were not tested because these hypotheses do not involve main effects of social class. Supplementary Table 12 provides the estimates for the 23 (hypotheses) × 9 (indicators) × 3 (moderators) = 621 interactions, along with the simple slopes. For the hypotheses marked with a superscript ‘a’, the statistical effect of social class observed in the secondary analysis was in the opposite direction to the original hypothesis for the majority of indicators; thus, the interactions refer to a strengthening or weakening effect of the observed social class effect in the secondary analysis rather than to the original hypothesized effect.

Our general hypothesis is that the effects of social class are strengthened by social class identification, system-justification beliefs and local inequality. In most cases, we considered the hypothesis supported when the effect of social class as hypothesized in Table 1 was strengthened. In the few cases where the effect in the secondary analyses was mostly in the opposite direction to the prediction, we considered our hypothesis supported when the observed (rather than the hypothesized) effect was strengthened (see the caption for Fig. 3 and ‘Summary of the deviations from preregistration’ in Methods). Generally, estimates highlighted in blue in Fig. 3 and Supplementary Table 12 indicate support for the hypothesis.

#### Social class identification

Our hypothesis was that social class differences are larger among individuals for whom social class is a more important part of their identity. The left panel of Fig. 3 shows whether, supporting our hypotheses, social class differences widen with increasing identification (strengthening interactions in blue) or, on the contrary, narrow (weakening interactions in orange). For this and subsequent analyses, Supplementary Table 12 provides estimates for all interactions, as well as simple slopes. For 35% of the hypotheses, in line with our predictions, social class identification strengthened the statistical effects of most social class indicators. Specifically, identification reinforced the tendency of individuals from higher social classes to see themselves as more agentic (H2′), to adopt a more self-aggrandizing view of the self (H5′–H6′), to perceive life trajectories as more susceptible to change (H18′), to attribute life events to more external causes (H19′), to display more ethical behaviour (H29′), to take more financial risks (H31′) and to engage in more utilitarian decision-making (H35′). For 9% of the hypotheses, contrary to our predictions, social class identification weakened the statistical effects of most social class indicators. Specifically, identification attenuated the tendency of individuals from higher social classes to report a higher sense of control (H1′) and self-esteem (H4′). For 52% of the hypotheses, social class identification did not significantly interact with the majority of indicators. For the remaining hypothesis (H3′), the results were mixed.

#### System-justification beliefs

Our hypothesis was that social class differences are larger among individuals who believe that society is fair. The middle panel of Fig. 3 shows whether, supporting our hypotheses, social class differences widen with increasing system justification (strengthening interactions in blue) or, on the contrary, narrow (weakening interactions in orange). For 52% of the hypotheses, in line with our predictions, system-justification beliefs strengthened the statistical effects of most social class indicators. Specifically, system justification reinforces the tendency of individuals from higher social classes to see themselves as more agentic (H2′); to adopt a more self-aggrandizing view of the self (H5′–H6′); to hold a more interdependent self-construal (H8′); to receive more social support from their network (H11′); to perceive life trajectories as more susceptible to change (H18′); to attribute life events to more external causes (H19′); to experience more compassion (H21′), love (H22′) and pride (H24′); to display more ethical behaviour (H29′); and to engage in more utilitarian decision-making (H35′). For 17% of the hypotheses, contrary to our predictions, system-justification beliefs weakened the statistical effects of most social class indicators. Specifically, system justification attenuates the tendency of individuals from higher social classes to report higher sense of control (H1′) and self-esteem (H4′), bias against lower-educated people (H15′), and preference for larger future gains over smaller immediate gains (H30′). For 22% of the hypotheses, system-justification beliefs did not significantly interact with the majority of indicators. For the remaining two hypotheses (H3′ and H23′), the results were mixed.

#### Local income inequality

Our hypothesis was that social class differences are larger among individuals who live in locales with higher income inequality. The right panel of Fig. 3 shows whether, supporting our hypotheses, social class differences widen with increasing inequality (strengthening interactions in blue) or, on the contrary, narrow (weakening interactions in orange). For 22% of the hypotheses, in line with our predictions, local income inequality strengthened the statistical effects of most social class indicators. Specifically, inequality reinforces the tendency of individuals from higher social classes to feel more entitled (H6′), to hold a more interdependent self-construal (H8′), to perceive life trajectories as more susceptible to change (H18′), to attribute life events to more external causes (H19′) and to engage in more utilitarian decision-making (H35′). For one of the hypotheses, local income inequality weakened the statistical effects of social class indicators on outcomes. Specifically, local income inequality attenuates the tendency of individuals from higher social classes to report a higher sense of control (H1′). For the remaining 74% of the hypotheses, local income inequality did not significantly interact with the majority of indicators.

#### Connection with the replications

We wish to highlight three instances where the original statistical effect of social class was not consistently replicated in either the primary or secondary analysis but was observed at a conditional value of the moderator in the tertiary analysis. First, for most indicators, we found that a higher social class was associated with perceiving life trajectories as less susceptible to change among individuals with low social class identification or weak system-justification beliefs. Second, for some indicators, we found that a higher social class was associated with a less interdependent self-construal among individuals with low social class identification, with weak system-justification beliefs or living in more economically unequal places. Third, and finally, for some indicators, we found that a higher social class was associated with fewer other-oriented emotions (that is, compassion and love) among individuals with weak system-justification beliefs. These results suggest that the replication of social class tendencies might sometimes depend on individual, ideological or structural factors.

#### Summary

Identification, system-justification and income inequality increased the predictive strength of social class for 1/3, 1/2 and 1/5 of outcomes, respectively. Interestingly, the hypothesized effects of social class on anticipation of change, interdependent self-construal and other-oriented emotions—which were not replicated in the previous parts—were observed at certain values of the moderators.

## Discussion

This article had three objectives. The first was to assess the overall replicability of the psychological effects of social class in four countries. We successfully replicated half of the selected hypothesized effects, with higher replication rates in the domain of the self and lower rates in the domain of behaviour. About 50% of the significant effects of social class were of a very small to small size (*η*_p_^2^ ≤ 0.01). The second objective was to compare the predictive strength of nine commonly used indicators of social class. Since these indicators capture different aspects of social class, we aimed to clarify how they differentially relate to psychological outcomes^51^. Despite their conceptual differences, our results suggest that these indicators generally lead to similar conclusions in terms of direction and significance. However, there were variations in effect sizes. Specifically, subjective indicators showed stronger associations with psychological outcomes than objective indicators. This may not be surprising, as subjective indicators inherently capture the psychological dimension of social class experience^52^. Importantly, it should be noted that our conclusions about indicators is contingent on both the outcomes and the specific measures examined. Regarding outcomes, variables beyond those studied here—such as political attitudes—are known to be strongly predicted by education^53–55^. Regarding the indicators, we selected those commonly used in the replicated studies (for example, a subjective SES ladder using the community as a comparison point), but we cannot rule out the possibility that alternative measures might have produced different effects (for example, a subjective SES ladder using society as a comparison point). The third objective was to test whether social class identification, system-justification beliefs and local income inequality strengthened the effect of social class. System justification was the moderator with the most significant interactions, followed by identification and then inequality. Across moderators, when the interactions were significant, social class differences tended to widen (rather than narrow) in the majority of cases (~80%) at high levels of the moderator. While our study focuses on these three specific moderators, our dataset includes numerous other variables that could be used to explore intersectionality between social class and other factors such as gender and ethnicity^56,57^.

### Re-evaluating key claims in the psychology of social class

**Fig. 4:**
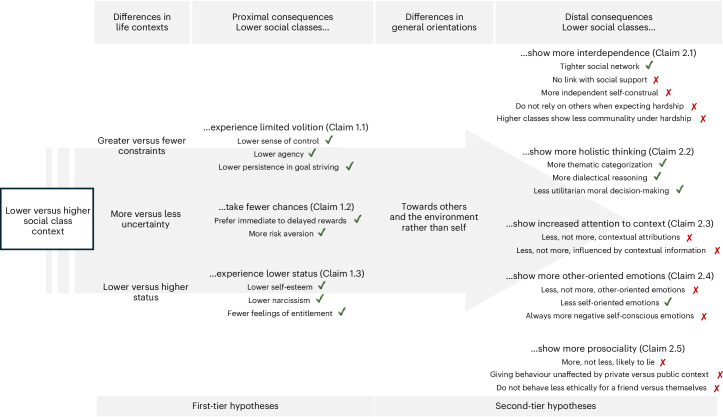
Summary of key hypotheses supported or unsupported by the data. Summary of the key hypotheses supported (green ticks) or unsupported (red crosses) by the data, categorized into two tiers and eight claims.

In light of these findings, we re-evaluate central claims in the psychology of social class to provide an updated overview of the state of the field. We have tried in good faith to give an account of the dominant trends (that is, effects observed in the majority of tests in the first two empirical sections), while recognizing that this account may not fully capture the nuances of the thousands of analyses performed. As seen in Fig. 4, we propose that most of the hypotheses examined can be categorized into two tiers.

The first tier includes hypotheses on how social class contexts differ in terms of constraint, uncertainty and status and how they shape proximal individual outcomes. This idea is not specific to the psychology of social class and can be found in other fields relevant to social stratification (for example, evolutionary-developmental approaches, welfare economics and social epidemiology)^25,58–61^. We identified three claims in this tier. Claim 1.1 is that lower social class contexts are high-constraint environments where individuals experience limited volition and freedom. Consistent with this claim, we found that individuals from lower social classes report a lower sense of control, agency and persistence. Claim 1.2 is that lower social class contexts are highly uncertain environments where taking chances carries potential costs. Supporting this claim, we found that individuals from lower social classes demonstrate greater financial risk aversion and a preference for immediate rewards over future benefits. Claim 1.3 is that individuals in lower social class contexts lack status. Consistent with this claim, we observed a pervasive education bias across countries, especially among individuals from higher social classes. Also consistent, we found that individuals from higher social classes benefit from higher self-esteem, which can veer into narcissistic tendencies and feelings of entitlement. Overall, these three claims are well supported by our data.

The second tier includes hypotheses on how the aforementioned differences in social class context (vis-à-vis constraint, uncertainty and status) translate into distinct general psychological orientations (towards the self, others and the environment) and shape more distal individual outcomes. These hypotheses are derived from the models of the psychology of social class^2,9,10^. We identified five claims in this tier. Claim 2.1 is that individuals from lower social classes have a more interdependent self-construal. Consistent with this claim, we found that individuals from lower social classes maintain tighter social networks; however, this did not translate into receiving more social support. Inconsistent with this claim, we mostly found that individuals from higher, not lower, social classes report a more interdependent self-construal and show more positive reactions to similarity to others. Moreover, a central aspect of this claim is that individuals from lower social classes rely on others to cope with hardship, whereas individuals from higher social classes rely on their financial resources. Contrary to this idea, we found that individuals from higher social classes expecting hardship do not increase their reliance on money but instead display a reduced communal orientation. Claim 2.2 is that individuals from lower social classes adopt a holistic thinking style whereas individuals from higher social classes adopt an analytical thinking style. Consistent with this claim, individuals from lower social classes consistently favour categorizations based on thematic rather than taxonomic relations and—at least for those in lower educational groups—use more dialectical reasoning (anticipating change). Moreover, they are less likely to sacrifice one person to save many in the trolley dilemma, which can also be interpreted as a more intuitive or less deliberative response, which is consistent with less analytical thinking^62^. Claim 2.3 is that individuals from lower social classes pay more attention to context. Inconsistent with this claim, we found that individuals from higher, not lower, social classes use more contextual explanations for life events and are more influenced by contextual information in an emotion appraisal task. Claim 2.4 is that individuals from lower social classes experience fewer self-oriented emotions and more other-oriented emotions. Inconsistent with this claim, we found that it is individuals from higher rather than lower social classes who report more other-oriented positive self-emotions, although they do report more self-oriented positive emotions (that is, contentment and pride). Moreover, they experience fewer negative self-conscious emotions. Together, these results indicate that individuals from higher social classes experience more pleasing emotions, regardless of whether these emotions are self- or other-oriented (lending further support to Claim 1.3 in terms of status). Claim 2.5 is that individuals from lower social classes are more prosocial. Inconsistent with this claim, we found that individuals from higher social classes are less likely to lie for career benefits. The idea that this prosociality among individuals from higher social classes is driven by self-interest is also unsupported, as their giving behaviour is not influenced by whether the context is private or public, and their unethical behaviour does not depend on whether the beneficiary is themselves or a friend. Upon exploring the main effect of social class on prosociality, we found that it varied on the basis of the specific indicator used and how prosociality was operationalized (lying, donating tickets or engaging in unethical behaviours). Similar inconsistencies are observed in the corpus of studies attempting to replicate the link between social class and prosociality^63–84^. In summary, among the five claims derived from the distinction between self-orientation and other and environment orientation, only the third claim about differences in cognitive style is supported by our data.

This conclusion raises questions about the validity of models in the psychology of social class that contrast psychological orientations towards ‘the self’ with those towards ‘others and the environment’. Our data suggest that such an opposition is not warranted: individuals from higher social classes seem to be more focused on both themselves and others than individuals from lower social classes. They exhibit both a more empowered self (for example, increased agency, self-esteem and risk-taking) and greater connection with others (for example, greater interdependence, greater appreciation of similarities, more supportive relationships, more prosocial behaviour, and greater love and compassion). We believe that these trends could be explained in a parsimonious way by differences in social class contexts with regard to constraints, uncertainty and status. Affluence, stability and high status may create a safer environment that encourages self-empowerment, challenge appraisals and eagerness, while still supporting open engagement with others. Conversely, scarcity, uncertainty and low status may give rise to a risk-laden environment that encourages self-preservation and threat appraisals, which potentially leads to distrust. Regarding the trends observed among individuals from higher social classes, other theoretical models support the idea that specific features of their environments may promote a focus on both the self and others. For instance, institutions such as higher education and the globalized marketplace promote individual autonomy and achievement, alongside a commitment to treating others as equal partners^85^. Regardless of the countries they reside in, individuals from higher social classes are more likely to spend time being socialized in these institutions and to adopt their norms^6^. As another example, individuals from higher social classes often find themselves in high relational mobility contexts, where they develop wider, looser and more far-reaching social networks (through travel, hobbies, occupations and studies)^86^. In such contexts, individuals grow accustomed to forming and breaking social bonds, making them experienced and motivated to attract others. This leads them to cultivate their individuality while remaining mindful of others^87^.

### Cross-cultural differences

Our samples were drawn from four countries, which constrains our ability to fully explore cross-cultural differences. That said, it is important to note that while the replication analyses yielded relatively consistent results across countries, there were some notable exceptions in India. For example, social class was positively associated with sense of control in France, the USA and Switzerland, but negatively in India. Furthermore, social class was associated with interdependence only in the Indian sample. These variations may reflect genuine differences in how social class shapes control beliefs^88^ or self- and other-orientation^89^ between WEIRD and non-WEIRD contexts. Alternatively, these variations may be due to suboptimal data quality in India, where participants demonstrated markedly shorter median response times for inductions and low reliability for scales with reversed items (note that this latter issue may also be a cultural specificity in India, as it has been observed in other studies^90,91^). To better investigate the role of culture in social class, future research could use cross-national survey data with careful attention to data quality to systematically investigate how the effects of social class vary as a function of factors such as power distance^92^, cultural tightness–looseness^93^, WEIRDness^41^ and religiosity^94^.

### Limitations

Several limitations must be acknowledged. First, the hypotheses examined were not selected from a random sample of papers but based on theoretical and feasibility reasons. Thus, our findings, including reproduction rates, cannot be generalized to the entire field. Second, we encountered several issues in the data collection process. In the Swiss, French and Indian samples, individuals in the lowest education group were underrepresented (by 6% to 16%). In Switzerland, the lower-than-expected response rate resulted in underpowered analyses for the longer tasks and experiments (see ‘Sampling plan’ in Methods). In India, difficulties in sample recruitment and issues with data quality necessitated ad hoc adjustments to the inclusion criteria (refer to ‘Sampling plan’ in Methods). Third, sense of power was included as an indicator of social class because it reflects the experience associated with high status. However, it is usually viewed as a distinct construct or potential mediator^4,22,95^. While we acknowledge these conceptual differences, our results indicate that sense of power produced similar effects to other indicators on most of the studied outcomes (though these effects were stronger).

## Conclusion

In summary, a replication rate of 50% suggests that some aspects of the field are robust, whereas others are less so. Surprisingly, most results did not vary on the basis of the social class operationalization, suggesting that the flexible use of indicators may not be the main reason for such a suboptimal rate. Furthermore, most results were relatively consistent across national contexts, suggesting that cultural differences—while present—are not as impactful as might have been thought. Finally, half of the hypothesized effects were moderated by at least one of our three moderators, showing how identifying with one’s social class, feeling that society is fair or living in a place where class segmentation is more pronounced contributes to widening psychological differences between social classes.

From a theoretical perspective, hypotheses directly related to differences in social class contexts (in terms of constraints, uncertainty and status) tended to replicate well. These first-tier hypotheses form the basis of many theories of social class, and future research can continue to build on this foundation with relative confidence. However, hypotheses derived from models that contrast individuals from lower and higher social classes in terms of a general orientation (towards the self versus the other/the environment) have not replicated as successfully. Rather, our results suggest that individuals from higher social classes, compared with individuals from lower social classes, tend to be more focused on both themselves and others. This conclusion has the potential to reshape our understanding of the psychology of social class and merits further exploration.

## Methods

### Ethics information

The project was approved by the Research Ethics Board of the University of Lausanne (C_SSP_032020_00004). Participants gave their informed consent at the beginning of the study. Participants recruited by panel providers (US, French and Indian samples) received various types of compensation (for example, gift cards), whereas participants directly recruited by a local team (Swiss sample) participated in a lottery to receive gift cards.

### Design

#### Selection of the studies

The studies were selected on the basis of a review conducted by the three authors. In June 2019, we used the reference lists of two recent reviews about the psychology of social class^10,96^ and further searched for empirical studies on a scientific publication search engine with three guiding criteria: the studies had to be theoretically relevant (testing a central claim of the various frameworks of the psychology of social class), cover a wide variety of domains (that is, self, cognition, emotion, relationships, decision-making and behaviour) and be feasible online. This process led to an initial selection of 21 studies. In December 2019, after securing funding for the project, we updated the list by searching for all new empirical studies that cited these 21 studies. To further ensure that we had not missed any important studies, when asking the authors of the original studies to review the protocol, we asked them to list findings they deemed important to replicate. This led us to remove one study and add two others.

#### Questionnaire

We invited the participants to complete an online questionnaire. Drawing on former large-scale replication initiatives^41,97^, we randomized the blocks of our questionnaire so that (1) the total duration would not exceed 20 min and (2) participants would not take part in more than one experiment. In addition to completing the nine measures of social class (~2 min), participants were randomly assigned to complete 2/3 of the short tasks or scales (~12–13 min) and 1/6 of the long tasks or experiments (~3–4 min). Two translation service companies translated the questionnaire from English into French (for the French and Swiss samples), Hindi (for the Indian sample), German and Italian (for the Swiss sample). The French, German and Italian versions of the questionnaire were double-checked by native speakers of each language.

##### Predictor variables

The participants first completed the nine measures of social class (for the country-based sample sizes, *α* values, means and standard deviations, see Supplementary Table 4; for the pooled within-country correlations, see Supplementary Table 10). The measures that were not used to build the quotas were counterbalanced (placed at the beginning of the questionnaire for half of the participants and at the end for the other half). As preregistered, for each hypothesis, we determined whether it was necessary to control for the order variable in a preliminary analysis (Supplementary Information, ‘General notes’, p. 20).

Education was measured using various categories for the highest level of education and combined into three groups representing the national population-based tertiles: (1) high school graduate or less versus two-year college degree versus four-year college degree or higher (US sample)^98^, (2) less than high school versus high-school graduate or two-year college degree versus three-year college degree or higher (French sample)^99^, (3) less than high school versus high school graduate versus some college or higher (Indian sample)^100^, and (4) less than high school versus high school and non-university diploma versus bachelor’s degree or higher (Swiss sample)^101^. For students, we used the highest level of education completed by either guardian. For all relevant analyses, education was contrast-coded. As preregistered, the planned contrast compared the lowest educational group to the highest educational group, but we used the codes −0.5 and +0.5 instead of −1 and +1 to facilitate interpretation of the estimates (that is, they represent the difference between the bottom and top groups).

Income was measured using bands corresponding to the national population-based household income deciles^102–105^. An open-ended question then asked participants to specify the exact amount of their household income. In the analysis, we used the response to the open-ended question or—if this response was missing or incoherent—the midpoint of the household income bands (see ‘Preregistration’). To adjust for household size, we used the Organisation for Economic Co-operation and Development (OECD) square-root scale (that is, equivalizing income by dividing household income by the square root of household size)^106^.

Occupation was measured using Hoffmeyer-Zlotnik’s classification scheme^107^. After reporting their current employment status, self-employed participants indicated the size of their farm or business, whereas employed participants indicated the level of autonomy/complexity of their job. Participants were assigned to one of five occupational class categories (from 1 (unskilled, semiskilled, manual workers) to 5 (far-reaching leadership tasks and decision-making powers)). For retirees and students, we determined their former occupational class category or the category of the main earner in the family, respectively. Deviating from the preregistration, unemployed participants were assigned a score of zero to avoid excluding a full category of persons from the samples.

Subjective SES was measured using the MacArthur Scale^18^. The participants were presented with a ten-rung ladder representing “where people stand in (their) local community”. They were asked to indicate where they thought they stand on the ladder (from 1 (bottom) to 10 (top)).

Childhood subjective SES was measured using an adapted version of the MacArthur Scale^108^. This time, the participants were asked to indicate where they and their families stood on the ladder when they were five to ten years old (from 1 (bottom) to 10 (top)).

Childhood SES was measured using a three-item scale (for example, “I grew up in a relatively wealthy neighborhood”; from 1 (strongly disagree) to 7 (strongly agree))^3^.

Social class self-categorization was measured by asking the participants to report their social class (from 1 (lower class) to 5 (upper class))^109^.

Financial scarcity was measured by asking the participants about the balance of their income and expenses (from 1 (saves a lot of money) to 5 (gets into debt); for evidence of the convergent validity of the instrument, see refs. ^110,111^).

Sense of power was measured using a three-item scale (for example, “I think I have a great deal of power”; from 1 (strongly disagree) to 7 (strongly agree))^22^.

##### Outcome variables: self-reported scales or short tasks

The participants then completed 2/3 of the short tasks or scales (for the country-based sample sizes, *α* values, means and standard deviations, see Supplementary Table 5). Unless otherwise specified, the response scales ranged from 1 (strongly disagree (or not at all)) to 7 (strongly agree (or completely)). Items belonging to the same scale were presented in a random order.

**The self**. Sense of control (H1/H20) was measured using the 12-item Personal Mastery and Perceived Constraints Scale (for example, “What happens in my life is often beyond my control”)^112^. Agency (H2) was measured using the Agency Scale, which asks participants the extent to which five traits describe them (for example, “self-confident”)^113^. Persistence in goal striving (H3) was measured using the five-item Persistence in Goal Striving Scale (for example, “When I encounter problems, I don’t give up until I solve them”)^114^. Self-esteem (H4) was measured using the four-item Rosenberg Self-Esteem Short Scale (for example, “I take a positive attitude toward myself”)^115^. Narcissism (H5) was measured using the six-item Narcissistic Admiration and Rivalry Questionnaire Short Scale (for example, “Being a very special person gives me a lot of strength”)^116^. Entitlement (H6–7) was measured using the nine-item Psychological Entitlement Scale (for example, “I honestly feel I’m just more deserving than others”)^117^. Inter/independent self-construal (H8) was measured using an adaptation of Singelis’ Self-Construal Scale, which asks participants the extent to which ten independent (for example, “I always express my opinions clearly”) and ten interdependent (for example, “I am concerned about what people think of me”) statements describe them (from 1 (does not describe me at all) to 5 (describes me very much))^16,118^. Negative reactions to reduced individuation (H9) were measured using three questions that participants respond to by expressing whether they feel good or bad after imagining a friend purchasing the same car as them^17^.

**Relationships**. Chaos (versus stability) (H12–13), communal orientation (H12) and obsession with money (H13) were measured as follows: first, the participants chose between a chaotic (with ups and downs) and a stable (steadily increasing) graph to represent the expected trajectory of their future economic well-being; second, they completed the 13-item Communal Orientation Scale (for example, “I often go out of my way to help another person”; from 1 (extremely uncharacteristic of me) to 7 (extremely characteristic of me))^119^ and the five-item Obsession subscale of the Money Beliefs and Behavior Scale (for example, “I feel that money is the only thing that I can really count on”)^120^. The scales were presented in a random order. Education and ethnic/national biases (H14–16) were measured with thermometer ratings (from 0 (very cold) to 100 (very warm)) of seven groups (that is, more-educated versus less-educated people as well as five ethnic groups—four outgroups and one ingroup, adapted to the cultural context)^19^. In our preregistered study protocol, we planned to test the equivalence between education and ethnic/national biases (H16). However, owing to an unforeseen shortcoming in the translation of the questionnaire in both the French and Swiss versions, the measure of national ingroup attitude was mistakenly replaced with a measure of national outgroup attitude, meaning that H16 could not be tested in these two samples.

**Cognition**. Thematic/holistic and taxonomic/analytical thinking styles (H17) were measured using the triad task, which includes 14 lists of three objects (for example, cow, grass and chicken), each involving one thematic pair (cow/grass) and one taxonomic pair (cow/chicken); the participants had to indicate which two objects were more closely related^16^. Contextual explanations (H19–20) were measured using ratings of eight events (for example, “being laid off at work”) on a seven-point bipolar scale ranging from 1 (individual primarily responsible) to 7 (outside forces primarily responsible)^11^. Anticipation of change (H18) was measured with the estimated likelihood (in per cent) that contradictory events would happen in eight different situations (for example, “Two kids are fighting at kindergarten. How likely is it that they will become lovers some day?”)^16^.

**Emotion**. Other- and self-oriented positive emotions (H21–24) were measured using the 12-item Dispositional Positive Emotions Scale assessing feelings of contentment (for example, “I am generally a contented person”), pride (for example, “I take great pride in my achievements”), compassion (for example, “I am a very compassionate person”) and love (for example, “I grow to love people who are kind to me”)^20^. Influence of contextual emotional information (H26) was measured using 12 pictures with a central character showing an emotion (anger, happiness or sadness) and four background characters showing either the same emotion (three pictures) or a different emotion (nine pictures). For each picture, the participants rated the anger, happiness and sadness of the central character using scales ranging from 1 (not at all) to 10 (very much)^11^.

**Social behaviour**. Unethical behaviours (H27) were measured as follows. The participants were asked to take the role of an employer negotiating a salary with a job candidate seeking a long-term position. The participants were told that the job would be eliminated after six months, but they had strong incentives to fill the position. Afterwards, they reported as a percentage how likely they would be to hide the truth about the position from the candidate^24^.

**Decision-making**. Utilitarian moral decision-making (H35) was measured using the so-called footbridge dilemma. After reading a scenario about a trolley heading down the tracks towards five workmen, the participants had to indicate whether it was appropriate or not to push a stranger onto the tracks to stop the trolley from killing the five workmen^27^. The preregistered replication procedure of the original study involved measuring social class using perceived resource availability and examining how this predicted utilitarian moral decision-making (H35). However, owing to an unforeseen shortcoming in the questionnaire construction process in both the French and Swiss versions, the measure was mistakenly omitted, meaning that H35 could not be tested in the primary analysis for these two samples.

##### Outcome variables: experiments or longer tasks

After completing the self-report scales or short tasks, the participants were randomly assigned to one of the five experiments or the social network task (for the country-based sample sizes, *α* values, means and standard deviations, see Supplementary Table 5).

**Relationships: social network task (H10–11)**. The participants were presented with three concentric circles centred on a small circle labelled “YOU”. First, we assessed the proportion of individuals in one’s inner circle. The participants were asked to report the initials of (1) the people to whom they are very close in the inner circle, (2) the people who are not so close but still important in the middle circle and (3) the people who are least important in the outer circle. Second, we assessed the social support received: the participants reported whether each of their network members showed more social support or annoyance^16^.

**Emotion: self-conscious negative emotions (H25)**. First, the participants self-rated nine baseline emotions (that is, self-conscious negative emotions (embarrassment, fear, guilt and worry) and five other negative emotions; from 1 (not at all) to 8 (very much))^21^. Second, the participants played the recipient in the dictator game, with a bogus other allocating ten raffle tickets between themselves and the participant. They were randomly assigned to one of two between-participants conditions: (1) in the near-equal sharing condition, the bogus other shared four of the ten tickets with the participant, and (2) in the low sharing condition, the bogus other shared one ticket. Third, the participants again completed the emotion measures.

**Social behaviour: unethical behaviours (H27)**. The participants were randomly assigned to one of two between-participants conditions: (1) in the self-benefit condition, they were asked the extent to which they would engage in eight unethical behaviours for their own benefit (from 1 (very unlikely) to 7 (very likely)), and (2) in the other-benefit condition, they were asked the extent to which they would engage in the same eight unethical behaviours for the benefit of a friend (using the same scale)^22^. ‘Self-beneficial’ was coded as +0.5, and ‘other-beneficial’ was coded as −0.5, contrary to the preregistration, which had the coding reversed.

**Social behaviour: prosocial behaviour (H28)**. The participants were asked to play the dictator in the dictator game twice and allocate ten raffle tickets between themselves and a bogus other. Participants were assigned to the two within-participants conditions in a counterbalanced order: (1) in the private condition, they were instructed that their gift would be anonymous, and (2) in the public condition, they were instructed that their gift would be accompanied by identifying information (that is, name and city of residence). Prosocial behaviour was measured using the number of raffle tickets allocated to the bogus other^23^.

**Decision-making: delayed reward and risk aversion (H30–33)**. The participants were randomly assigned to one of two between-participants conditions: (1) in the mortality condition, they read a text presenting the world as dangerous, and (2) in the control condition, they read a text about how to choose a rain jacket^25^. Preference for delayed reward was measured using seven hypothetical choices involving an increasing monetary reward for delayed options (for example, “Do you want to get $100 tomorrow or $110 90 days from now?” (with $10 increments)), and risk aversion was measured using seven hypothetical choices involving an increasing monetary reward for a guaranteed option compared to a 50% chance of getting a larger amount (for example, “Do you want a 50% chance of getting $800 OR [to] get $100 for sure?” (with $100 increments)). The order of presentation of the measures was counterbalanced.

**Decision-making: cognitive performance (H34)**. The participants were randomly assigned to one of two between-participants conditions: (1) in the hard financial problems condition, they had to think about how to solve four financial problems involving large amounts of money, and (2) in the easy financial problems condition, they had to think about how to solve similar problems involving smaller amounts of money (the amounts of money were adapted to the standard of living in the national population)^26^. The participants were then instructed to keep thinking about the financial problems while performing three Raven’s Standard Progressive Matrices presented in a random order. Cognitive performance was measured using the number of matrices correctly solved. For each country, we pretested Raven’s Matrices (focusing on matrices of average difficulty according to the manual^121^) in a pilot study and selected three matrices that were correctly solved by approximately 50% of individuals. The details of these pilot studies can be found in the Supplementary Information (Table S-E5a, p. 31).

##### Moderators

At the beginning of the questionnaire, the participants filled out two scales used for the moderation analyses (that is, social class identification and system-justification beliefs) and provided information about their place of residence (which enabled us to link their responses to local data on income inequality). Social class identification was measured using a single-item scale asking the participants to rate the importance of their social class in describing them^109^. System-justification beliefs were measured using the four-item System Justification Scale (for example, “In general, I find society to be fair”)^122^. Local income inequality indicators were extracted from public economic data and merged with the survey data. We used the most recent local Gini coefficient estimates available at the lowest level of geographic aggregation. Specifically, we used the 2022 ACS-based zip-code-level Gini coefficients for the US sample^123^, the 2020 INSEE-based municipality-level Gini coefficients for the French sample^124^, the 2020 AFC-based municipality-level Gini coefficients for the Swiss sample^125^ and the 2012 NSSO-based district-level Gini coefficient for the Indian sample^126^.

##### Control variables

Control variables similar to those used in the original studies were included at the end of the questionnaire. In the USA, India and Switzerland (in the German and Italian questionnaires), political orientation was measured using a scale ranging from 1 (very liberal/progressive) to 7 (very conservative). In France and Switzerland (in the French questionnaire), the labels were adapted to range from 1 (completely on the left) to 7 (completely on the right). In all samples, religiosity was measured using a scale ranging from 1 (not at all religious) to 7 (very religious). In the US sample, ethnicity was measured using the US Census Bureau’s categories (for example, White and African American) plus Latinx. In the French and Swiss samples, the ethnicity question was not displayed because (1) in France, ethnicity-based statistics are prohibited except under very specific circumstances, and (2) in Switzerland, it is also a very sensitive question. Instead, the participants were asked about their nationality to differentiate citizens from non-citizen residents. In the Indian sample, after consulting with the translation company, we decided to use a question about religious affiliation (for example, Hinduism or Islam).

##### Attention checks

In line with the current recommendations about quality checks^127^, we used two easy attention checks, one at the beginning of the questionnaire and another at the end (for example, “This is an attention check; please select ‘somewhat agree’”). Qualtrics mistakenly did not solely retain the US and French participants who had passed both attention checks as originally preregistered. Instead, Qualtrics used the more common criterion of retaining participants who had passed at least one attention check (for a similar approach, see ref. ^128^). For the studies involving a manipulation, we did not include a manipulation check because (1) the original studies had already demonstrated the effectiveness of the induction using a manipulation check, and (2) manipulation checks do not provide information relevant to construct validity except in pilot studies^129^.

### Sampling plan

#### Selection of the countries

Our total sample comprised participants residing in four countries: the USA, France, India and Switzerland. The selection of the countries was based on both practical and cultural reasons. Practical reasons involved—among other things—cost, feasibility and the authors’ familiarity with the national context. For instance, including the USA allowed for replications with the same population used in 89% of the selected studies, whereas including Switzerland allowed us to draw a random probability sample at a low cost. Cultural reasons involved ensuring a degree of diversity in terms of cultural attitudes towards power inequality and hierarchy (for example, the power distance index^130^) or WEIRDness (Western educated industrialized rich democracies^85^). For instance, France is one of the western European countries that is the most culturally distant from the USA, with a long tradition of class protests and the endorsement of an overall socialist agenda^131^, whereas India is a LMIC (lower/middle-income country^132^) and the only LMIC for which we could recruit a large quota-based sample.

#### Data collection

Our total sample comprised four samples: (1) three quota-based samples (the US, French and Indian samples) recruited by Qualtrics (a market research company) and (2) one random representative sample (the Swiss sample) recruited by our team.

##### Power analysis

We aimed to reach a power of at least 0.95 for each individual hypothesis. For each country, 9,000 × 2/3 = 6,000 participants per self-reported scale or short task was estimated to yield a power of 0.99999999712 to detect one small individual effect (*η*_p_^2^ = 0.01). For each country, 9,000 × 1/6 = 1,500 participants per experiment or long task was estimated to yield a power of 0.97311815015 to detect one small effect (*η*_p_^2^ = 0.01).

##### Exclusion criteria

For the US, French and Indian samples, Qualtrics removed and replaced participants who provided low-quality responses (for example, participants failing both (USA/France) or at least one (India) of the two attention checks, rushers (participants who completed the questionnaire in less than half the median time) and straight-liners (participants always providing the same responses to the Likert scales)). During data collection for the Indian sample, we observed problems with the distribution of response times to questions and a low median survey duration. We thus asked Qualtrics to apply a stricter criterion for the attention checks in the Indian sample, specifically by retaining only those participants who had passed both checks. Additionally, we asked Qualtrics to implement a new, non-preregistered criterion across all samples: retaining only participants who completed the survey in nine minutes or more (deemed the minimum possible time to complete the survey correctly). Finally, during the data cleaning phase, we identified duplicates on the basis of IP addresses and demographics and asked Qualtrics to remove and replace them. For the Swiss sample, we applied the same exclusion criteria. Supplementary Table 2 provides the complete list of inclusion criteria used in each sample. For the experiments using lengthy experimental inductions (that is, E4 (mortality) and E5 (financial problems)), we tested the hypotheses with and without participants who read the induction in less than half the median time (we urge readers to exercise caution when interpreting the results without rushers, as excluding them can reduce power below 0.95).

##### US, French and Indian samples

**Original plan**. Qualtrics agreed to deliver three quota-based samples of 9,000 each for US, French and Indian adult residents (above 18). To achieve sample distributions that match those of the underlying populations, Qualtrics used quota sampling with five quotas: (1) income (deciles in the USA and France, quintiles in India), (2) education, (3) gender, (4) age and (5) region. In the case of India, Qualtrics could guarantee only three quotas—namely, income, gender and age. Specifically, each sample was built gradually so that the sample distribution would match the official national distribution for each quota (for example, the US sample income deciles would be similar to the official national income deciles^103^). The main advantage of quota sampling is that it ensures that each stratum of the population is equally represented in the sample, but its main limitation is that the sample is non-probabilistic (that is, participants self-select into the panel and are therefore not necessarily representative of the underlying population).

**Final samples**. The final samples met or exceeded the targeted sample sizes: *N*_US_ = 9,019, *N*_FR_ = 9,160 and *N*_IN_ = 9,556. As can be seen in Supplementary Table 3, most of the differences between the sample and population estimates are negligible, indicating that the majority of quotas were effectively fulfilled. However, the lowest education groups were underrepresented in both France (32.2% in the sample versus 40.6% in the population) and India (52.3% in the sample versus 68.4% in the population). This issue was particularly pronounced in India, presumably due to the high prevalence of people with no formal education in the population, many of whom are illiterate and de facto excluded from the study. Given this context, Qualtrics had to relax the education quota to be able to complete the study.

##### Swiss sample

**Original plan.** We aimed to build a representative sample of 9,000 Swiss residents. To achieve representativeness, we used random sampling: (1) the Swiss Federal Statistical Office drew a random sample (stratified by canton) of ~50,000 Swiss addresses; (2) a public institution (the DAL) printed, folded and assembled 50,000 letters of invitation to participate in our study; and (3) the University of Lausanne sent these 50,000 letters. We expected a response rate between 1/5 and 1/6 (the response rate for a Swiss probability-based web survey without monetary incentive^133^). The main advantage of random sampling is that each individual in the population has the same probability of being invited to participate in the study, but its main limitation is that individuals from specific subgroups (for example, people with lower incomes) may have a lower response rate (non-response bias). In the context of our research, we believe that the advantage of random sampling offsets the limitation of quota sampling and vice versa.

**Final sample.** The final samples did not meet the targeted sample size, *N*_CH_ = 5,801, owing to a lower-than-expected response rate of approximately 12%. Originally, our plan was that if the final sample size was below 7,734 (that is, statistical power below 0.95), we would ask Qualtrics to complete the sample using the same quota sampling approach used in the US and French samples. However, after discussions with the chief editor and senior editors, and consultation with an anonymous external expert, we decided to proceed with the current sample. The reasons were that the sample characteristics were reasonably close to those of the population (Supplementary Table 3) and that statistical power remained above 0.95 for the correlational studies, although it fell to 0.83 for the longer tasks and experiments (before the exclusion of the rushers). It was therefore thought that combining a probability sample with an opt-in sample could entail more costs in terms of representativeness and sample quality than benefits in terms of power.

#### Missing data

For each particular model, we used listwise deletion to handle missing data (thus, the sample size varies from one analysis to another).

### Analysis plan

#### Reliability criteria for item inclusion in the scales

We calculated Cronbach’s *α* to test the reliability of the multi-item scales. As preregistered, when Cronbach’s *α* was below 0.60, we attempted to identify and remove problematic items. There were three such cases: interdependent self-construal in the Swiss sample (H8, *α* = 0.58), pride in the Swiss sample (H24, *α* = 0.56) and self-esteem in the Indian sample (H4, *α* = 0.34). No item removals improved reliability, and, as preregistered, we proceeded with the analysis. However, since low reliability can increase the rate of false negatives^134^, we added a note in Table 2 to indicate when a scale’s reliability was unsatisfactory.

#### Confirmatory analysis: primary analysis (replications)

For each country and each hypothesis, we used the same analytical approach used in the original study (when possible). The full list of hypotheses, power estimates and analyses performed can be found in the Design Table of the Stage 1 protocol and the preregistration on OSF. All predictors and outcomes were standardized. All deviations from the original studies are listed in Supplementary Table 1, and the list of deviations from the preregistered analyses can be found in the Supplementary Information (‘List of deviations’, pp. 42–44).

#### Confirmatory analysis: secondary analysis (indicators)

For each hypothesis, we repeated the planned analysis (described in the Design Table of the Stage 1 protocol on OSF) without control variables (for reasons of comparability) and while pooling country-specific samples (for reasons of brevity and intelligibility). We did not test hypotheses where social class was not the predictor (H14 and H16), where the focus was on an indirect effect (H20) or where a null moderation was predicted (H32–H33). We ran nine regression models with each of the nine measures of social class (see ‘Design’) and country-based dummies (that is, country fixed effects^135^). This analytical approach enabled us to discard all between-cluster variations, thereby eliminating all potential between-country confounders and producing unbiased estimates of the pooled within-country effects of each social class indicator^136^. All variables were standardized. Income was rescaled within countries to account for differences in monetary units, rather than broken into quintiles as preregistered. The reason was that rescaling provided much more precise estimates, while using quintiles created discrepancies between the primary analysis (using log-income) and the others. When the analyses involved interactions, we employed double-demeaning to ensure unbiased within-country estimations^137^. We then performed a series of postestimation *Z*-tests comparing the standardized coefficient estimates associated with the nine measures of social class. We did not formulate hypotheses regarding specific differences between the nine measures in terms of predictive strength.

#### Confirmatory analysis: tertiary analysis (moderators)

For each hypothesis and each measure of social class, we again repeated the primary analysis without control variables, while pooling country-specific samples and using fixed-effects modelling to produce the average within-country estimates. This time, we additionally included one of the three potential moderators: social class identification, system-justification beliefs and local income inequality (in 3 (moderators) × 9 (measures of social class) separate models). Specifically, the fixed-effects regression equation was as follows:

1 $$\begin{array}{rcl}\displaystyle{\stackrel{\rightharpoonup}{Y}}_{ij}&=&{\beta}_{1}\times {\stackrel{ \rightharpoonup }{\rm{Indicator}}}_{ij}+{\beta}_{2}\times {\stackrel{\rightharpoonup}{{\rm{Moderator}}}}_{ij}\\ && \displaystyle +{\beta}_{3}\times\stackrel{\rightharpoonup}{{\stackrel{\rightharpoonup}{\rm{Indicator}}_{ij}\times{\stackrel{ \rightharpoonup }{\rm{Moderator}}}_{ij}}}+{u}_{ij}\end{array}$$

where *Y*_*ij*_ is the outcome for participant *i* in country *j*, *u*_*ij*_ is the error term and the diacritical lines symbolize demeaning (double-demeaning in the case of interaction). To maintain a high level of statistical power, we focused on potential moderations of the original main effects (that is, we avoided testing second-order interactions). All variables were standardized.

We expected the interaction term *β*_3_ to reveal a larger social class effect when social class identification, system justification or local income inequality was high (the prediction regarding system-justification beliefs was more tentative). For local income inequality, we first estimated the design effect (which quantifies the degree to which a multilevel sample differs from a simple random sample); as it was always below 1.5, we refrained from using multilevel modelling^138^. The full list of hypotheses, power estimates and planned analyses can be found in the Design Table of the Stage 1 protocol on OSF.

#### Correction for multiple tests

For the primary analysis (replications), the different confirmatory tests were not considered as belonging to the same family of tests^139^. We thus used the conventional *α* level—that is, *α* = 0.05 (for example, as in ref. ^49^). For the secondary and tertiary analyses, exploratory tests pertaining to a similar outcome but using different measures of social class were considered as belonging to the same family of tests. We thus used the sequential Bonferroni procedure as a correction for multiple tests. For each outcome, we used an adjusted *α* level of *α*_adj_ = 0.05/9 = 0.0056 for the measure of social class with the smallest *P* value, *α*_adj_ = 0.05/8 = 0.0063 for the measure with the second-smallest *P* value, *α*_adj_ = 0.05/7 = 0.0071 for the measure with the third-smallest *P* value and so on^139^.

#### Null results

In the primary analysis, when observing a null hypothesized effect, we determined whether the effect is absent by using equivalence testing^140^. Equivalence testing enables one to reverse the null and alternative hypotheses, so that the burden of proof rests in proving equivalence^141^. Specifically, we used *f* = |0.05| as the smallest effect size of interest (which corresponds to *r* = |0.05| or *d* = |0.10| and arguably pertains to a trivial effect^34,142,143^). For each occurrence, we compared the hypothesized effect size to *f* = −0.05 (our lower equivalence bound) and *f* = +0.05 (our upper equivalence bound) using one-sided postestimation Wald tests^141^. If both tests are significant, the effect is interpreted as equivalent to zero; otherwise, the result is deemed to be inconclusive. The results are presented in Supplementary Table 8. In the secondary and tertiary analyses, we interpreted a null effect as inconclusive.

#### Data transformation

We did not anticipate data transformation. However, owing to the severe skewness of income distribution in each country, and because robust estimation of standard errors was insufficient to address the problem, we log-transformed income in all analyses (for example, as in ref. ^144^).

### Summary of the deviations from preregistration

A comprehensive list of deviations from the registered report and preregistration document is provided in the Supplementary Information (‘List of deviations’, pp. 42–44). While these deviations are noted throughout the paper, we summarize the most important ones here. First, issues in questionnaire design led to the omission of national ingroup attitudes and the perceived resource availability scale in both the French and Swiss samples. As a result, we were unable to appropriately test H16 (ethnic bias) and H35 (moral decision-making) in these samples. Second, three deviations involved ad hoc changes in our exclusion criteria: (1) after realizing that the samples contained duplicate responses, we decided to systematically exclude them on the basis of IP addresses and demographic information; (2) miscommunication with the panel provider during data collection in the USA and France led to the exclusion and replacement of participants who failed both attention checks rather than just one; and (3) in two experiments using reading materials (E4–E5), we prioritized response quality over sample size by excluding participants identified as rushers. Third, before conducting inferential tests, we made three data-processing adjustments: we recoded occupation to retain unemployed participants; we log-transformed income to correct for skewness; and, when pooling samples, we not only log-transformed income but also rescaled it within each country to account for differences in monetary units. Fourth, in the tertiary analysis, we applied a sequential Bonferroni correction for multiple tests to maintain consistency with the secondary analyses. Finally, in the tertiary analysis, when the observed effect of social class was mostly inconsistent with the hypotheses across indicators in the secondary analyses, we interpreted the moderating effect as strengthening versus weakening on the basis of the observed rather than the predicted effect in the few cases where most effects in the secondary analyses were in the opposite direction to the prediction. This adjustment was necessary to ensure interpretability, as our general hypothesis posited that any effect of social class would be strengthened by the moderators.

### Protocol registration

The Stage 1 protocol, as accepted by the journal on 29 October 2021, can be found at ref. ^145^.

### Reporting summary

Further information on research design is available in the Nature Portfolio Reporting Summary linked to this article.

## Supplementary information

Supplementary Information Supplementary Tables 1–12, detailed report of all replication analyses undertaken, calculation of the MDE with 95% power in Fig. 1, and list of deviations from the original registered report and preregistration.

Reporting Summary

## Data Availability

The data are available on the OSF page for the project (https://osf.io/3tjzs/). The pooled dataset and a comprehensive codebook can be found in ‘/Pooled Dataset (With Codebook)’, whereas the four country-specific datasets, along with the full materials (including all questionnaires in the local languages), are available in ‘/Individual Datasets (with Materials)’.

## Code availability

The data were analysed using either Stata version 18.5 or R version 4.4.1. The scripts are available on the OSF page for the project (https://osf.io/3tjzs/). The scripts to reproduce the results from the primary, secondary and tertiary analyses can be found in ‘/Scripts/Primary Analysis (Replications)’, ‘/Scripts/Secondary Analysis (Indicators)’ and ‘/Scripts/Tertiary Analysis (Moderators)’, respectively.
