# Author Correction: Advancing the psychology of social class with large-scale replications in four countries

**DOI:** 10.1038/s41562-026-02419-2

**Published:** 2026-02-02

**Authors:** Anatolia Batruch, Nicolas Sommet, Frédérique Autin

**Affiliations:** 1 LIVES Centre, University of Lausanne, Lausanne, Switzerland; 2 Centre de Recherches sur la Cognition et l’Apprentissage (UMR 7295), Université de Poitiers, Université de Tours, CNRS, Poitiers, France

**Keywords:** Social policy, Sociology, Psychology, Human behaviour, Decision making

Correction to: *Nature Human Behaviour* 10.1038/s41562-025-02234-1, published online 15 July 2025.

In the version of this article initially published, there was an error in the orthogonal contrast used to test the effect of education in India. Specifically, the contrast was coded as 1/3 and 2/3 rather than –1/3 and 2/3, affecting the value or the placement of some estimates in Figs. 2, 3 and the Supplementary Information. In Fig. 2, row H9, “Negative reaction to reduced individuation (+)”, first column, “Education (linear contrast)”, the original value “–0.26^AAA*^” has been amended to read “–0.27^AAA*^.” In Fig. 3, row H21, “Compassion,” last column, “Moderator: local income inequality”, the original values and bars indicating “306” have been updated to “207.” In the Supplementary Information, Supplementary Tables 7 (rightmost column), 11 (leftmost column of results) and 12 (leftmost most column of results) have been updated. These changes do not affect the substantive interpretation of the results, the conclusions of the analyses or the main text. The figures and Supplementary Information have been amended in the HTML and PDF versions of the article. The OSF files have also been updated to correct the error.

