# Supplementary material for: Advancing the psychology of social class with large-scale replications in four countries
Source: Nat Hum Behav. 2025 Jul 15;9(11):2382–403. doi: 10.1038/s41562-025-02234-1 (PMC12634430; doi:10.1038/s41562-025-02234-1)
Supplement: Supplementary file 1 — Supplementary Tables 1–12, detailed report of all replication analyses undertaken, calculation of the MDE with 95% power in Fig. 1, and list of deviations from the original registered report and preregistration. [file 41562_2025_2234_MOESM1_ESM.pdf]

nature human behaviour

Supplementary information https://doi.org/10.1038/s41562-025-02234-1

Advancing the psychology of social class with large-scale replications in four countries

In the format provided by the authors and unedited

Supplementary Information for

ADVANCING THE PSYCHOLOGY OF SOCIAL CLASS WITH LARGE-SCALE REPLICATIONS IN FOUR COUNTRIES

Table of content

Table S1. Deviation Table 2
Table S2. Inclusion Criteria Used in Each Sample 5
Table S3. Sample Characteristics Along With National Population Characteristics 6
Table S4. Sample Size, Reliability, and Descriptive Statistics for Predictors/Moderators 7
Table S5. Sample Size, Reliability, and Descriptive Statistics for Replication Variables 8
Table S6. Ratio of Consistent Results to Total Test Specifications (for Replication Rates) 11
Table S7 - Primary Analysis. Coefficient Estimates, Confidence Intervals, and P-Values 13
Table S8 - Primary Analysis. Equivalence Tests 18
Detailed Report of All Replication Analyses Undertaken 20
General Notes 20
The Self (Nine Hypotheses) 20
Relationships (Seven Hypotheses) 23
Cognition (Four Hypotheses) 25
Emotions (Six Hypotheses) 26
Behavior (Three Hypotheses) 28
Decision-Making (Six Hypotheses) 29
Calculation of the MDE with 95% Power In Figure 1 33
Table S9 - Primary Analysis. Minimum Detectable Effect Size and Partial Eta Squared 34
Table S10 - Secondary Analysis. Pooled Within-Country Correlations Between Indicators 36
Table S11 - Secondary Analysis. *P*-values for Each Relevant Hypothesis and Indicators 37
Table S12 - Tertiary Analysis. Coefficient Estimates for the Interactions and Simple Slopes 38
List of Deviations From the Original Registered Report and Preregistration 42

Table S1. *Deviation Table (Differences Between Authors' Original Studies and Our Preregistered Studies)*

	Type	Deviation
**S1/2**	Study design	• Participants were shown graphs displaying increasing or decreasing inequality in the US and asked to provide explanations of economic inequality, before answering questions about their sense of control and their social class.<br>• Original study settings: Laboratory setting.
	Measurement	• In the original study, parental education was coded into three categories: 1 = less than high school graduate, 2 = high school graduate, general education diploma, or some college, and 3 = college graduation or higher. Participants provided classifications of parental education for both parents. Parental education ratings for both parents were assigned a code number from 1 to 3.<br>• Family household income was coded into seven categories: (a) under $15,000, (b) $15,001–$25,000, (c) $25,001–$35,000, (d) $35,001–$50,000, (e) $50,001–$75,000, (f) $75,001–$100,000, and (g) over $100,000.
	Analysis	Ø
**S3**	Study design	• Original study settings: Unspecified.
	Measurement	• A short four-item validated scale was used in lieu of the original Rosenberg Self-Esteem Scale (1965) (Tambs & Røysamb, 2014).<br>• In the original study, education was coded: 1 = eighth grade/junior high or less; 2 = some high school; 3 = high school graduate/GED; 4 = 1+ years of college, no degree; 5 = 2-year college/vocational school; 6 = 4/5 years college, bachelor's degree; 7 = at least some graduate school.
	Analysis	• We also tested the effect of social class on each individual scale of the self-orientation composite index (i.e., self-esteem, agency and persistence in goal striving).
**S4**	Study design	Ø
	Measurement	• A short six-item validated version was used in lieu of the Narcissistic Admiration and Rivalry Questionnaire (Leckelt and al., 2018).
	Analysis	Ø
**S5/6**	Study design	• Original study settings: Online/Laboratory setting.
	Measurement	Ø
	Analysis	Ø
**S7**	Study design	Ø
	Measurement	• A short four-item scale was used for the System-justification beliefs (Vargas‑Salfate et al., 2018).<br>• In the original study, occupation was coded: 1 = unemployed or semi-unemployed; 2= of unskilled or manual workers, 3 = of skilled workers; 4 = of small or self-employed proprietors; 5 = of lower middle-class occupations; 6 = of upper-middle-class occupations; 7 = of upper-class occupation.
	Analysis	Ø
**S8a/d**	Study design	• Original study settings: Unspecified.
	Measurement	• In the original study, education was coded: Working-class = some college or less; Middle-class = completed college or more.
	Analysis	Ø
**S9**	Study design	• Original study settings: Unspecified.
	Measurement	• In the original study, parental education was coded: Working-class = No parent with a 4-year college degree; Middle-class = One parent at least with a 4-year college degree.
	Analysis	Ø

Table S1. *Continued*

	Type	Deviation
**S10**	Study design	Ø
	Measurement	• In the original study, total household income was coded in eight categories: (a) <$15,000, (b) $15,001–$25,000, (c) $25,001–$35,000, (d) $35,001–$50,000, (e) $50,001–$75,000, (f) $75,001–$100,000, (g) $100,001–$150,000, or (h) >$150,000.
	Analysis	Ø
**S11**	Study design	• In the original study, participants were primed with chaos-related or negatively valenced words. In the replication study, we measured the expectation of chaos.<br>• Original study settings: Laboratory setting.
	Measurement	Ø
	Analysis	Ø
**S12**	Study design	• The groups were adapted to the cultural context of the country of study.
	Measurement	• In the original study, education was coded: 1 = high school diploma or less; 2 = some college but no degree; 3 = 2-year college degree; 4 = 4-year college degree; 5 = post-graduate degree.
	Analysis	• To test a similar hypothesis in another study, the authors performed a Bayesian repeated measures ANOVA. In the replication study, an average of the four ethnic-outgroup ratings was computed. The smallest effect size of interest was defined as corresponding to Cohen's $d = 0.2$. An equivalence test was performed on the difference between the education and ethnic/national bias.
**S13**	Study design	Ø
	Measurement	Ø
	Analysis	• The authors measured the indirect effect with a Sobel test. We performed a mediation analysis with bootstrap percentile confidence intervals.
**S14**	Study design	Ø
	Measurement	• In the original study, total household income was coded in 19 categories: (a) <$5,000) to 19 (s) >$175,000.
	Analysis	• Three covariates used in the original study were not available: Enthusiasm, Awe, and Amusement.
**E1**	Study design	• In the original study, a research assistant explained the roles in the game, and the distributor was supposedly doing the task in the lab at the same time. For our part, we told participants that the roles were allocated randomly, and participants waited until they received their role.<br>• Original study settings: Laboratory setting.
	Measurement	• In the original study, the composite social class index used total household income coded in six categories: (a) <$15,000, (b) $15,001–$45,000, (c) $45,001–$60,000, (d) $60,001–$75,000, (e) $75,001–$100,000, (f) >$100,000 and education coded: 1 = high school graduation; 2 = college graduation, 3=advanced degree completion.
	Analysis	• A covariate used in the original study (Neuroticism) was not available.<br>• The authors of the original studies observed that the social class x (un)equal sharing interaction was not significant for other types of negative emotions and concluded that "effects on emotion ratings following the Dictator game appear to be localized to negative self-conscious affect" (p. 6). To test this more formally, we ran a mixed-design 2 (Time: baseline vs. post-manipulation) x 2 Type of emotion (negative self-conscious emotion vs. non-self-conscious negative emotion) x 2 Condition (equal sharing vs. low sharing) x 3 Education (low vs. middle vs. high) with the last two factors as between-participant factors. We tested the same higher-order interaction with education and income in separate models, using a regression-based approach.

Table S1. *Continued*

	Type	Deviation
**S15**	Study design	• In the original study, participants were administered a personal sense of control manipulation. They were asked to type out their thoughts regarding a situation in which they either experienced little or no control or a great deal of control before performing the emotion task. We only replicated the main effect of social class on the emotion task.<br>• Original study settings: Laboratory setting.
	Measurement	Ø
	Analysis	Ø
**E2**	Study design	• In the original study, the authors conducted a pretest to verify that the self-beneficial versus other-beneficial scenarios did not differ in how unethical or immoral they were viewed. In the replication study, we didn't pre-test the material used in the original study.<br>• Original study settings: Unspecified.
	Measurement	• In the original study, annual family household income was coded into eight categories: (a) <€10,999, (b) €11,000 to €20,999, (c) €21,000 to €30,999, (d) €31,000 to €40,999, (e) €41,000 to €50,999, (f) €51,000 to €60,999, (g) €61,000 to €70,999, & (h) >€71K.
	Analysis	Ø
**E3**	Study design	• In our material, we told participants that the roles in the game were allocated randomly, and participants waited until they received their role. Several other scales were added to test mediation, but we did not include them in the study.
	Measurement	• In the original study, the authors tested for social class differences (in income, education and parental education) between two samples: MTurkers and MBA samples. As social class differences were significant for the three social class indices, MTurkers were coded as lower social class whereas MBA samples were coded as higher social class.
	Analysis	Ø
**S16**	Study design	• In the original study, a morality of greed scale was added to test a mediation, but we did not include it in the replication study.
	Measurement	Ø
	Analysis	• A covariate used in the original study (Morality of greed scale) was not available.
**E4**	Study design	• In the original study, After the Dangerous world or control induction, and before answering the risk and time preferences questions, participants played either an Ultimatum Game (UG) or a Dictator Game (DG).
	Measurement	Ø
	Analysis	• A covariate used in the original study (Morality of greed scale) was not available.
**E5**	Study design	• In the original study, the Raven trials were selected from a pool of 12 Raven's matrices based on a pilot study to ensure that the trials were neither too difficult nor too easy. In the replication study, we ran a similar pilot with six Raven's matrices.<br>• Original study settings: Laboratory setting.
	Measurement	• In the original study, household income was divided by the square root of household size. Then the measure was median split at $70,000.
	Analysis	Ø
**S17**	Study design	Ø
	Measurement	Ø
	Analysis	• A covariate (Moral reasoning) used in the original study was not available.

Table S2. *Inclusion Criteria used in Each Sample*

	Recruitment	Sampling	Inclusion Criteria				
			1. Progress[a]	2. Completion time[b]	3. Attention checks[c]	4. Straigliners	5. Duplicates[e]
**USA**	Qualtrics	Quota	Survey completed in full	≥ 540 sec.	At least one correct check	Based on all survey scales (74)	Based on IPv4 + demographics (10)
**France**	Qualtrics	Quota	Survey completed in full	≥ 540 sec.	At least one correct check	Based on all survey scales (60)	Based on IPv4 + demographics (53)
**Switzerland**	Authors	Probability	No missing for focal variables (1,225)	≥ 540 sec. for full surveys (5)	At least one correct check (664)	Based on all survey scales (0)	Based on IPv4 + demographics (20)
**India**	Qualtrics	Quota	Survey completed in full	≥ 540 sec.	Both checks must be correct	Based on all survey scales (53)	Based on IPv4 + demographics (28)

Note. For Switzerland, the number of participants excluded after applying each inclusion criterion is given in parentheses. For other countries, these numbers are provided only for the inclusion criteria directly applied by us, and not by the recruiting company (i.e., Inclusion Criteria 4-5).

[a] To make the most of our more limited sample, we used a more liberal version of Inclusion Criteria #1 for Switzerland. Specifically, we retained any participants with at least one non-missing predictor and outcome variable (rather than focusing on full responses).

[b] We harmonized Inclusion Criteria #2 across countries. We asked Qualtrics to retain any participant who completed the survey in nine minutes or more (540 seconds) and did the same for Switzerland. This time corresponded to half the median time observed in the Indian soft launch, and was considered the minimum possible time to complete the survey correctly.

[c] Qualtrics mistakenly used a more liberal version of Inclusion Criterion #3 for the USA and France than originally described in the pre-registration. As is commonly done, they retained participants who had passed at least one attention check, rather than both (for a similar criterion, see Verschuere et al., 2023). We used the same method for Switzerland. However, to address data quality issues, Qualtrics retained participants who had passed both attention checks in the Indian sample.

[e] During the data cleaning phase, we identified duplicates based on IP addresses and demographic characteristics (i.e. gender, education level, employment status, income and household size). We did not rely solely on IP addresses to identify duplicates, as different participants may be connecting from the same router (e.g., the same public Wi-Fi hotspots). To maintain the integrity of our datasets, we asked Qualtrics to implement an additional, non-pre registered inclusion criterion for each sample. Specifically, we asked them to retain only the first instance of duplicate entries and replace all subsequent duplicates. We adopted the same approach for Switzerland.

Table S3. *Sample Characteristics Along with National Population Characteristics*

	**USA (*N* = 9,019)**	
	Sample	Population
Women	53.9%	50.8%
Mean age	46.4	47.5
Education[a]		
High school graduate or less	31.4%	36.5%
Two-year college degree or less	30.5%	27.9%
Four-year college degree or higher	38.1%	35.7%
Median annual household income	$ 67,500	$ 70,784

	**France (*N* = 9,160)**	
	Sample	Population
Women	51.6%	51.7%
Mean age	43.8	42.4
Education[a]		
Less than high-school	32.2%	40.6%
Two-year college degree or less	43.2%	32.7%
Three-year college degree or higher	24.6%	26.7%
Median annual household income	€ 30,000	€ 30,620

	**Switzerland (*N* = 5,801)**	
	Sample	Population
Women	52.5%	50.4%
Mean age	48.9	49.7
Education[a]		
Less than high-school	41.2%	47.5%
High school / non-university diploma	24.3%	22.9%
Three-year college degree or higher	34.5%	29.6%
Median annual household income	CHF 90,000	CHF 96,0000

	**India (*N* = 9,556)**	
	Sample	Population
Women	52.1%	51.5%
Mean age	37.9	38.7
Education[a]		
Less than high-school[b]	52.3%	68.4%
High school graduate	21.8%	18.4%
College degree	26.0%	13.2%
Median annual household income	₹ 225000	₹ 225000

Note. This table shows that most of the differences between the sample and population estimates are negligible (i.e. less than ± 5%, as per our agreement with Qualtrics). Figures in red indicate exceptions: The lowest education groups were underrepresented in Switzerland (-6.3%), France (-8.4%), and India (-16.1%). This trend was particularly pronounced in India due to the high prevalence of people with no formal education in the population, many of whom are illiterate and de facto excluded from the study. Given this context, Qualtrics had to relax the education quota to be able to complete the Indian sample. US, French, Swiss, and Indian population estimates were gathered/calculated from (i) the U.S. Census Bureau (2022a, 2022b, 2022c), (ii) the INSEE (2020a, 2021, 2022, 2023), (iii) the FSO (2022a, 2022b, 2023) and the SHP Group (2023), and (iv) the UN (2022a, 2022b) and the World Bank (2022), respectively.

[a] The education categories used in each sample correspond to the preregistered categories used for the planned contrast.

[b] Most of the participants in this group had only attended primary school or less.

Table S4. *Sample Size, Reliability, and Descriptive Statistics by Country for Each Social Class Predictor and Each Moderator*

Self-reported Variables	USA				France				Switzerland				India			
	n	α	*M*	*SD*	*n*	α	*M*	*SD*	*n*	α	*M*	*SD*	*n*	α	*M*	*SD*
Social class predictors																
Education (proportion in the top group)	9,019	n/a	0.38	.	9,160	n/a	0.25	.	5,787	n/a	0.35	.	9,556	n/a	0.27	.
Equivalized annual household income	9,019	n/a	51.4K	52.1K	9,160	n/a	21.0K	12.8K	5,701	n/a	68.7K	42.0K	9,556	n/a	227K	500K
Occupation (level of autonomy)[A]	8,337	n/a	2.71	1.63	8,457	n/a	2.64	1.31	4,740	n/a	3.35	1.12	7,649	n/a	3.04	1.45
Subjective SES (MacArthur Scale)[B]	9,011	n/a	5.92	2.09	9,130	n/a	5.95	1.63	4,957	n/a	6.68	1.52	9,310	n/a	6.63	2.43
Childhood subjective SES[B]	9,011	n/a	5.67	2.18	9,130	n/a	5.64	1.9	4,958	n/a	5.72	1.86	9,312	n/a	6.37	2.53
Childhood SES (Griskevicius's scale)[C]	9,012	0.82	3.8	1.53	9,130	0.78	3.43	1.35	4,966	0.75	3.76	1.31	9,319	0.86	4.34	1.5
Social class self-categorization[D]	8,992	n/a	3.11	1.01	9,125	n/a	3.09	0.85	4,948	n/a	3.43	0.72	9,278	n/a	3.28	1.13
Financial scarcity[E]	9,014	n/a	2.66	1.16	9,125	n/a	2.56	1.02	4,940	n/a	2.38	0.95	9,339	n/a	2.19	0.92
Sense of power[C]	9,013	0.83	4.1	1.38	9,127	0.76	3.77	1.13	4,949	0.75	3.88	1.04	9,339	0.84	4.68	1.34
Moderators																
Social class identification[F]	9,010	n/a	4.57	3.11	9,128	.n/a	5.06	2.33	4,950	n/a	4.36	2.59	9,314	n/a	6.86	2.77
System-justification beliefs[C]	9,013	0.88	3.7	1.53	9,126	0.87	3.33	1.32	4,948	0.8	4	1.12	9,339	0.87	4.68	1.35
Local income inequality[G]	5,679	n/a	0.44	0.05	2,433	n/a	0.36	0.05	1,500	n/a	0.41	0.06	461	n/a	0.35	0.06

Note. 'n/a' means 'not applicable'. This applies in cases where the variable is measured using a single-item scale, making the calculation of Cronbach's alpha irrelevant, or when the variable is categorical, making the calculation of standard deviation irrelevant.

[A] Occupations were classified into five groups ranging from 1 = "unskilled/semi-skilled workers" to 5 = "individuals with far-reaching leadership roles" using the method described in Hoffmeyer-Zlotnik & Warner (2011). In addition, unemployed participants were assigned a value of 0.

[B] The variables were assessed using a 10-point response scale ranging from 1 = "bottom of the ladder" to 10 = "top of the ladder"

[C] The variables were assessed using a 7-point response scale ranging from 1 = "strongly disagree" to 7 = "strongly agree"

[D] The variable was assessed using a 5-point response scale ranging from 1 = "lower class" to 5 = "upper class"

[E] The variable was assessed using a 5-point response scale ranging from 1 = "you have saved a lot of money" to 5 = "you have gotten into debt"

[F] This variable was assessed using a 11-point response scale ranging from 0 = "not at all important" to 10 = "extremely Important"

[G] We used the 2022 ZIP code-based Gini index for the US (U.S. Census Bureau, 2022d), the 2020 municipality-based Gini index for France (INSEE, 2020b) and for Switzerland (AFC, 2020), and the 2012 district-based Gini coefficient for India (Mohanty et al., 2016).

Table S5. *Sample Size, Reliability, and Descriptive Statistics by Country for Each Study Replication Variables*

ID	Self-reported Variables	USA				France				Switzerland				India			
		n	α	*M*	*SD*	*n*	α	*M*	*SD*	*n*	α	*M*	*SD*	*n*	α	*M*	*SD*
**S1/2**	Sense of control[a]	5,896	.86	4.65	0.99	5,998	.83	4.50	0.84	2,748	.86	5.17	0.82	5,748	.63	3.98	0.68
**S3**	Agency[a]	6,019	.84	3.88	1.38	5,996	.78	4.47	1.14	3,048	.77	4.74	0.98	6,235	.74	4.34	1.19
**S3**	Persistence in goal striving[a]	6,018	.88	5.15	1.23	5,996	.84	5.16	1.15	3,048	.82	5.46	0.95	6,235	.84	5.09	1.19
**S3**	Self-esteem[a]	6,018	.73	4.83	1.31	5,996	.66	4.60	1.15	3,048	.73	5.36	1.09	6,234	.34	4.56	0.96
**S3**	Omnibus self-orientation[b]	6,018	.86	0.00	0.77	5,996	.85	0.00	0.79	3,048	.85	0.00	0.79	6,232	.78	0.00	0.72
**S4**	Narcissism[a]	6,034	.81	3.52	1.25	6,137	.81	3.40	1.13	3,080	.71	2.89	0.93	6,546	.79	4.68	1.11
**S5/6**	Entitlement[a]	6,020	.88	3.83	1.22	5,977	.84	3.85	0.97	2,779	.81	3.21	0.92	6,238	.83	4.68	0.98
**S7**	System-justification beliefs[a]	5,598	.88	3.70	1.53	5,573	.87	3.33	1.32	2,903	.80	4.00	1.12	5,120	.87	4.68	1.35
**S8a**	Independent self-construal[c]	5,898	.78	3.91	0.59	6,078	.74	3.65	0.60	3,334	.64	3.39	0.55	5,825	.86	3.88	0.69
**S8a**	Interdependent self-construal[c]	5,898	.75	3.42	0.63	6,078	.69	3.42	0.57	3,334	.58	3.34	0.49	5,825	.83	3.71	0.69
**S9**	Reactions to deindividuation[d, Item 1]	6,063	n/a	0.91	n/a	6,003	n/a	0.88	n/a	2,749	n/a	0.95	n/a	6,309	n/a	0.85	n/a
**S9**	Reactions to deindividuation[d, Item 2]	6,058	n/a	0.86	n/a	6,003	n/a	0.82	n/a	2,751	n/a	0.91	n/a	6,312	n/a	0.83	n/a
**S9**	Reactions to deindividuation[d, Item 3]	6,058	n/a	0.91	n/a	6,006	n/a	0.88	n/a	2,750	n/a	0.95	n/a	6,309	n/a	0.85	n/a
**S8b**	Individuals in one's inner circle[e]	1,450	n/a	0.43	0.17	1,346	n/a	0.42	0.16	625	n/a	0.36	0.17	972	n/a	0.40	0.14
**S8b**	Social support received[e]	1,248	n/a	0.72	0.21	1,086	n/a	0.72	0.21	497	n/a	0.84	0.17	542	n/a	0.62	0.17
**S10**	Communal orientation[a]	5,873	.78	4.71	0.76	5,817	.75	4.56	0.71	2,709	.73	4.86	0.67	6,196	.88	4.42	0.66
**S10**	Chaos vs. stability[d]	5,873	n/a	0.45	n/a	5,817	n/a	0.47	n/a	2,709	n/a	0.32	n/a	6,196	n/a	0.42	n/a
**S11**	Obsession with money[a]	5,868	.85	3.28	1.44	5,807	.80	3.25	1.23	2,735	.68	2.20	0.88	6,159	.81	4.59	1.25
**S12**	Education bias[f,g]	5,882	n/a	6.41	24.96	6,128	n/a	2.28	24.59	3,277	n/a	4.07	24.11	5,798	n/a	8.61	27.92
**S12**	Education vs. ethnic bias[f,g]	5,882	n/a	4.31	31.68	n/a	n/a	n/a	n/a	n/a	n/a	n/a	n/a	5,798	n/a	15.10	37.75
**S8c**	Thematic/holistic thinking[e]	6,065	n/a	0.83	0.21	6,182	n/a	0.87	0.16	3,373	n/a	0.82	0.18	6,594	n/a	0.90	0.17
**S8d**	Anticipation of change[g]	5,893	.84	44.90	16.18	6,036	.76	45.00	13.46	3,309	.69	39.67	12.00	5,850	.89	60.55	19.59
**S13**	Contextual explanations[a]	5,896	.76	3.35	1.07	6,015	.66	3.54	0.90	2,745	.65	2.98	0.79	5,748	.86	3.80	1.36
**S14**	Compassion (other-oriented)[a]	6,019	.78	5.40	1.09	5,977	.80	5.25	1.10	2,733	.66	5.17	0.96	6,210	.77	5.29	1.14
**S14**	Love (other-oriented)[a]	6,019	.78	5.39	1.07	5,977	.72	5.11	1.04	2,733	.60	5.28	0.89	6,210	.80	5.38	1.14

Table S5. *Continued*

ID	Self-reported Variables	USA				France				Switzerland				India			
		n	α	*M*	*SD*	*n*	α	*M*	*SD*	*n*	α	*M*	*SD*	*n*	α	*M*	*SD*
**S14**	Contentment (self-oriented)[a]	6,019	.80	4.86	1.23	5,977	.77	4.45	1.14	2,733	.82	4.92	1.13	6,210	.81	5.18	1.18
**S14**	Pride (self-oriented)[a]	6,019	.76	5.40	1.04	5,977	.60	4.88	1.01	2,733	.56	4.92	0.93	6,210	.79	5.38	1.15
**E1**	Self-conscious emotions (T1)[h]	1,545	.87	2.80	1.71	1,615	.85	2.93	1.73	720	.77	2.10	1.12	1,697	.85	3.86	1.77
**E1**	Self-conscious emotions (T2)[h]	1,545	.89	2.16	1.59	1,615	.86	2.29	1.56	720	.68	1.47	0.78	1,697	.87	3.59	1.82
**E1**	Other negative emotions (T1)[h]	1,545	.85	2.60	1.57	1,615	.87	2.62	1.64	720	.79	1.71	0.97	1,694	.89	3.84	1.72
**E1**	Other negative emotions (T2)[h]	1,545	.86	2.40	1.53	1,615	.90	2.47	1.67	720	.80	1.62	1.20	1,694	.91	3.64	1.80
**S15**	Contextual influence on emotion[i]	6,062	n/a	0.86	0.92	6,135	n/a	0.92	0.92	2,819	n/a	0.98	0.86	6,547	n/a	0.97	1.15
**E2**	Self/other-based unethical conduct[a]	1,435	.81	3.77	1.26	1,507	.67	4.04	0.98	743	.60	3.59	0.93	1,584	.87	4.61	1.22
**E3**	Private/public prosocial behavior[f]	1,518	n/a	0.03	2.19	1,568	n/a	0.29	2.51	700	n/a	0.61	2.52	1,713	n/a	0.02	1.87
**S16**	Unethical behaviors[g]	6,025	n/a	35.22	27.33	6,090	n/a	41.39	26.74	2,794	n/a	23.61	26.20	6,495	n/a	31.32	23.98
**E4**	Delayed reward[e]	1,500	.90	0.37	0.43	1,471	.91	0.47	0.39	687	.92	0.66	0.37	1,618	.82	0.38	0.33
**E4**	Risk aversion[e]	1,500	.81	0.73	0.29	1,472	.82	0.75	0.27	689	.74	0.72	0.22	1,619	.77	0.56	0.31
**E5**	Performance (Raven's matrices)[e]	1,427	n/a	0.54	0.34	1,508	n/a	0.59	0.33	733	n/a	0.78	0.30	1,643	n/a	0.47	0.39
**S17**	Utilitarian decision-making[d]	2,614	n/a	0.30	n/a	6,043	n/a	0.28	n/a	3,286	n/a	0.12	n/a	5,759	n/a	0.58	n/a

Note. When several analyses were carried out for the same study, *n* denotes the analytical sample size of the first analysis. 'n/a' means' not applicable.' The n/a values for αs occur when the variable pertains to a task (e.g., **S8b**), a single-item scale (**S9**, **S10**, **S16**, **S17**), a difference score (**S12**, **E3**), an index (**S15**), or a performance measure (**S17**). The n/a values for *SD*s, occur when the mean is a proportion (**S9**, **S10**, **S17**). As preregistered, we attempted to remove problematic items from scales with α < .60, but no removals improved reliability. No statistical information is reported for the entitlement variable in **S7**, as this information is already provided in **S5/6**.

[a] The variables were assessed using a 7-point response scale, with higher scores reflecting higher levels on each construct.

[b] The variable was calculated by averaging the standardized scores of agency, persistence in goal striving, and self-esteem.

[c] The variables were assessed using a 5-point response scale, with higher scores reflecting higher levels on each construct.

[d] For these variables, *M*s represent proportions: (i) the proportion of participants in the sample who reacted positively rather than negatively to reduced individuation (**S9**); (ii) the proportion of participants who expected their future economic well-being to be chaotic rather than stable (**S10**); (iii) the proportion of participants who chose the utilitarian option rather than the deontological option in the trolley dilemma (**S17**).

[e] For these variables, *M*s represent *averaged* proportions: (i) the average proportion of individuals in one's inner circle relative to the total in all circles (inner, middle, outer) (**S8a**); (ii) the average proportion of social support received relative to the combined total of social support and annoyances experienced (**S8a**); (iii) the average proportion of holistic/thematic relative to taxonomic/analytic categorizations (**S8a)**; (iv) the average proportion of preferences for delayed rewards over immediate rewards (**E4**); (v) the average proportion of preferences for safe choices over risky ones (**E4**); (vi) the proportion of correct responses on Raven's Matrices (**E5**).

[f] For these variables, *M*s represents average difference scores: (i) the average difference in thermometer ratings of the higher-educated group minus the lower-educated group (**S12**); (ii) the average difference in bias for education groups minus ethnic/national groups (**S12**); (iii) the average difference in ethical behaviors (i.e., giving between 0 and 10 raffle tickets to a stranger) in private contexts minus public contexts (**E3**).

[g] The variables were assessed using a 100-point response scale, with higher scores reflecting higher levels on each construct.

[h] The variables were assessed using a 8-point response scale, with higher scores reflecting higher levels on each construct.

[i] The variable is an index representing the standard deviation for all the ratings of emotions corresponding to the emotion expressed by the focal person (while ignoring the filler emotion ratings).

Table S6. *Ratio of Consistent Results to Total Test Specifications by Hypothesis and Country*

ID	Hypothesis	US	FR	CH	IN
**H1**	Social class $^{+}$→ Sense of control	2/2	2/2	2/2	0/2
**H2**	Social class $^{+}$→ Agency	2/2	1/2	2/2	1/2
**H3**	Social class $^{+}$→ Persistence in goal striving	2/2	2/2	2/2	1/2
**H4**	Social class $^{+}$→ Self-esteem	2/2	2/2	2/2	1/2
**H2/4**	Social class $^{+}$→ Omnibus self-orientation measure[A]	n/a	n/a	n/a	n/a
**H5**	Social class $^{+}$→ Narcissism	1/1	1/1	0/1	1/1
**H6**	Social class $^{+}$→ Entitlement	1/1	1/1	0/1	1/1
**H7**	Social class × System-justification $^{+}$→ Entitlement	1/1	0/1	0/1	0/1
**H8**	Social class $^{-}$→ Interdependent self-construal[A]	n/a	n/a	n/a	n/a
**H9**	Social class $^{+}$→ Negative reactions to deindividuation	0/6	0/6	0/6	0/6
**H10**	Social class $^{-}$→ Individuals in one's inner circle	1/1	0/1	1/1	0/1
**H11**	Social class $^{-}$→ Social support received	n/a	n/a	n/a	n/a
**H12**	Social class × Stability/Chaos $^{-}$→ Communal orientation	0/1	0/1	0/1	0/1
**H13**	Social class × Stability/Chaos $^{+}$→ Obsession with money	0/1	0/1	0/1	0/1
**H14**	Education bias	1/1	1/1	1/1	1/1
**H15**	Social class $^{+}$→ Education bias	1/1	1/1	1/1	1/1
**H16**	Education bias = Ethnic/national bias[B]	0/1	n/a	n/a	0/1
**H17**	Social class $^{-}$→ Thematic/holistic thinking style	1/1	1/1	1/1	1/1
**H18**	Social class $^{-}$→ Anticipation of change	0/1	1/1	1/1	1/1
**H19**	Social class $^{-}$→ Contextual explanations	0/2	0/2	2/2	0/2
**H20**	Social class $^{+}$→ Sense of control $^{-}$→ contextual explanation[B]	n/a	n/a	1/1	n/a
**H21**	Social class $^{-}$→ Compassion (other-oriented emotion)	1/2	0/2	0/2	0/2
**H22**	Social class $^{-}$→ Love (other-oriented emotion)	0/2	0/2	0/2	0/2
**H23**	Social class $^{+}$→ Contentment (self-oriented emotion)	2/2	2/2	2/2	0/2
**H24**	Social class $^{+}$→ Pride (self-oriented emotion)	2/2	1/2	1/2	2/2
**H25**	Social class × Sharing × Time $^{-}$→ Self-conscious emotions	0/2	0/2	0/2	0/2
**H25′**	Social class × Sharing × Time × Type $^{-}$→ Negat. emotions[A]	n/a	n/a	n/a	n/a
**H26**	Social class $^{-}$→ Influence of contextual information	0/1	0/1	0/1	0/1
**H27**	Social class × Self/Other benefits $^{+}$→ Unethical behavior	0/4	0/4	0/4	0/4
**H28**	Social class × Private/Public $^{-}$→ Prosocial behavior	0/2	0/2	0/2	0/2
**H29**	Social class $^{+}$→ Unethical behaviors	0/1	0/1	0/1	0/1
**H30**	Social class $^{+}$→ Preference for delayed reward	0/2	0/2	0/2	2/2
**H31**	Social class $^{-}$→ Risk aversion	2/2	0/2	2/2	2/2
**H32**	Social class × Mortality/control $^{\emptyset}$→ Delayed reward	2/4	2/4	0/4	0/4
**H33**	Social class × Mortality (vs. control) $^{\emptyset}$→ Risk aversion	1/4	4/4	0/4	0/4
**H34**	Social class × Hard/Easy problems $^{+}$→ Performance	0/2	1/2	0/2	0/2
**H35**	Social class $^{+}$→ Utilitarian moral decision-making[B]	2/2	na	na	2/2
	Percentage of fully replicated effects	46.9	36.7	41.9	31.3
	Percentage of partially replicated effects	9.4	13.3	3.2	9.4
	Percentage of failure to replicate	43.7	50	54.9	59.3
	Total number of hypotheses considered	32	30	31	32

Note. 'n/a' means 'not applicable.' To be considered replicated, the effects had to have significant estimates for at least half of the model specifications used in the original main analyses.

[A] Four effects were not included in the calculation of the replication rates. First,the effect of social class on interdependent self-construals (**H8**) and on social support received (**H11**) were not considered because the original authors observed a null effect. Second, the

higher-order interaction between social class, sharing condition, time, and type of emotions (**H25′**) was not considered because the original authors did not conduct this test. Third, the effect of social class on the omnibus measure of self-orientation (**H2/4**) was not considered because it is redundant with the former test (the omnibus measure is the combination of agency, persistence in goal striving, and self-esteem).

[B] Three effects could not be tested in some samples and were not included in the calculation of the replication rates for those samples. Regarding the comparison between the education bias and the ethnic/national bias (**H16**), the effect of social class on utilitarian moral decision-making (**H35**), and errors in the configuration of the questionnaire rendered the analysis of the French and Swiss samples impossible. Regarding the mediation analysis (**H20**), we refrained from testing the indirect effect when the total effect of social class on contextual explanations was not significant, meaning that this test was not applicable to the US and French samples.

Table S7 - Primary Analysis. *Coefficients Estimates, Confidence Intervals, and P-Values by Country in All Study Replications*

ID	Predictor	Outcome	USA		France		Switzerland		India	
			B [95% CI]	*p*	*B* [95% CI]	*p*	*B* [95% CI]	*p*	*B* [95% CI]	*p*
**H1**	Subjective SES[w]	Sense of control	.06 [.03, .09][C]	***	.09 [.06, .11][C]	***	.33 [.29, .37][C]	***	-.12 [-.15, -.10][R]	***
**H1**	Subjective SES[w/o]	Sense of control	.07 [.05, .10][C]	***	.11 [.08, .13][C]	***	.34 [.30, .37][C]	***	-.07 [-.09, -.04][R]	***
**H2**	Subjective SES[w]	Agency	.32 [.29, .34][C]	***	.15 [.13, .18][C]	***	.15 [.12, .19][C]	***	.23 [.21, .25][C]	***
**H2**	Education (planned)[w]	Agency	.30 [.24, .36][Cc]	***	.04 [-.03, .11][N]	.244	.16 [.08, .24][C]	***	-.09 [-.16, -.03][R]	.003
**H2**	Education (orthogonal)[w]	Agency	-.07 [-.12, -.02]	.009	-.02 [-.07, .03]	.445	.05 [-.02, .13]	.166	-.27 [-.32, -.21]	***
**H3**	Subjective SES[w]	Persistence	.19 [.16, .21][C]	***	.10 [.08, .13][C]	***	.13 [.10, .17][C]	***	.07 [.05, .10][C]	***
**H3**	Education (planned)[w]	Persistence	.27 [.21, .33][C]	***	.12 [.05, .19][C]	***	.14 [.06, .22][C]	.001	.15 [.09, .21][Nc]	***
**H3**	Education (orthogonal)[w]	Persistence	.01 [-.04, .07]	.676	.04 [-.02, .09]	.169	.07 [.00, .15]	.063	-.39 [-.45, -.34]	***
**H4**	Subjective SES[w]	Self-esteem	.25 [.23, .27][C]	***	.12 [.10, .15][C]	***	.23 [.19, .26][C]	***	.00 [-.02, .03][N¶]	.760
**H4**	Education (planned)[w]	Self-esteem	.29 [.23, .35][Cc]	***	.23 [.17, .30][C]	***	.12 [.05, .20][C]	.002	.59 [.52, .65][Cc¶]	***
**H4**	Education (orthogonal)[w]	Self-esteem	-.06 [-.11, -.01]	.024	.03 [-.02, .08]	.209	.02 [-.06, .10]	.581	-.13 [-.18, -.07]	***
**H2/4**	Subjective SES[w]	Omnibus score	.33 [.30, .35][C]	***	.16 [.14, .18][C]	***	.22 [.18, .25][C]	***	.14 [.12, .17][C]	***
**H2/4**	Education (planned)[w]	Omnibus score	.38 [.32, .44][Cc]	***	.17 [.10, .23][C]	***	.18 [.10, .25][C]	***	.30 [.24, .36][Nc]	***
**H2/4**	Education (orthogonal)[w]	Omnibus score	-.05 [-.10, .00]	.055	.02 [-.03, .07]	.434	.06 [-.01, .14]	.109	-.37 [-.42, -.31]	***
**H5**	Subjective SES[w]	Narcissism	.27 [.24, .29][C]	***	.17 [.14, .19][C]	***	.01 [-.02, .05][N]	.492	.14 [.12, .17][C]	***
**H6**	Subjective SES[w]	Entitlement	.25 [.22, .27][C]	***	.11 [.08, .14][C]	***	-.09 [-.12, -.05][R]	***	.17 [.14, .19][C]	***
**H7**	Occupation × SJ beliefs[w/o]	Entitlement	.07 [.05, .09][C]	***	.02 [.00, .05][Cm]	.096	.02 [-.01, .06][N]	.203	-.02 [-.05, .00][R]	.031
**H8**	Education (planned)[w/o]	Interdependence	.04 [-.02, .11][N]	.157	.04 [-.03, .10][N]	.283	.07 [-.01, .15][Rm¶]	.090	-.28 [-.34,- .22][Cc]	***
**H8**	Education (orthogonal)[w/o]	Interdependence	-.02 [-.21,- .10]	***	.03 [-.02, .09]	.187	-.10 [-.18, -.02]	.013	.09 [.04, .16]	.002
**H9**	Education (planned)[w]	Deindividuation[Item1]	-.39 [-.60, -.17][R]	***	-.05 [-.26, .15][N]	.604	-.45 [-.84, -.07][R]	.022	-1.0 [-1.19, -.81][R]	***
**H9**	Education (orthogonal)[w]	Deindividuation[Item1]	-.01 [-.20, .18]	.951	-.08 [-.24, .08]	.35	.07 [-.30, .44]	.702	.11 [-.06, .27]	.223
**H9**	Education (planned)[w/o]	Deindividuation[Item1]	-.44 [-.66, -.23][R]	***	-.06 [-.26, .15][N]	.592	-.35 [-.72, .01][Rm]	.057	-1.0 [-1.19, -.81][R]	***
**H9**	Education (orthogonal)[w/o]	Deindividuation[Item1]	.01 [-.18, .20]	.894	-.08 [-.24, .08]	.307	.09 [-.26, .44]	.609	.11 [-.06, .27]	.222
**H9**	Education (planned)[w]	Deindividuation[Item2]	-.23 [-.41, -.05][R]	.014	.00 [-.18, .17][N]	.995	-.24 [-.55, .06][N]	.12	-.62 [-.79, -.45][R]	***
**H9**	Education (orthogonal)[w]	Deindividuation[Item2]	.04 [-.12, .20]	.607	-.08 [-.22, .05]	.224	-.20 [-.52, .12]	.215	.26 [.10, .41]	***
**H9**	Education (planned)[w/o]	Deindividuation[Item2]	-.29 [-.47, -.11][R]	.001	.00 [-.17, .18][N]	.967	-.07 [-.35, .21][N]	.607	-.62 [-.79, -.44][R]	***
**H9**	Education (orthogonal)[w/o]	Deindividuation[Item2]	.06 [-.09, .22]	.429	-.09 [-.23, .04]	.184	-.22 [-.52, .08]	.143	.26 [.10, .41]	***
**H9**	Education (planned)[w]	Deindividuation[Item3]	-.28 [-.49, -.06][R]	.011	.04 [-.17, .25][N]	.732	-.55 [-.94, -.15][R]	.007	-.91 [-1.10, -.72][R]	***
**H9**	Education (orthogonal)[w]	Deindividuation[Item3]	-.09 [-.28, .11]	.381	.08 [-.08, .24]	.345	-.19 [-.60, .22]	.365	.15 [-.02, .32]	.086

Table S7 - Primary Analysis. *Continued*

ID	Predictor	Outcome	USA		France		Switzerland		India	
			B [95% CI]	*p*	*B* [95% CI]	*p*	*B* [95% CI]	*p*	*B* [95% CI]	*p*
**H9**	Education (planned)$^{w/o}$	Deindividuation^{Item3}	-.35 [-.56, -.14]^{R}	.001	.03 [-.18, .24]^{N}	.785	-.42 [-.79, -.05]^{R}	.025	-.91 [-1.10, -.72]^{R}	***
**H9**	Education (orthogonal)$^{w/o}$	Deindividuation^{Item3}	-.06 [-.25, .13]	.538	.06 [-.09, .22]	.426	-.16 [-.54, .22]	.408	.15 [-.02, .32]	.087
**H10**	Education (planned)$^{w/o}$	Inner circle	-.37 [-.49, -.24]^{C}	***	-.11 [-.26, .03]^{N}	.133	-.31 [-.50, -.13]^{C}	.001	.15 [.00, .30]^{R}	.049
**H10**	Education (orthogonal)$^{w/o}$	Inner circle	.05 [-.06, .16]	.350	-.04 [-.15, .07]	.474	.04 [-.14, .21]	.682	-.01 [-.15, .13]	.905
**H11**	Education (planned)$^{w/o}$	Social support	.16 [.03, .30]^{R}	.020	.16 [-.01, .32]^{Rm}	.059	.15 [-.05, .36]^{N}	.147	.35 [.15, .55]^{R}	.001
**H11**	Education (orthogonal)$^{w/o}$	Social support	-.02 [-.14, .10]	.713	.03 [-.09, .15]	.666	-.13 [-.33, .07]	.191	.02 [-.17, .21]	.850
**H12**	Income × Chaos^{w}	Communal orient.	-.02 [-.08, .03]^{N}	.386	-.06 [-.11, -.01]^{Cp}	.012	.04 [-.03, .12]^{N}	.227	-.17 [-.21, -.12]^{Cp}	***
**H13**	Subjective SES × Chaos^{w}	Obsession money	-.01 [-.06, .04]^{N}	.605	.01 [-.04, .06]^{N}	.627	-.05 [-.13, .03]^{N}	.233	-.12 [-.17, -.07]^{R}	***
**H14**	Education target group$^{w/o}$	Thermometer	.26 [.23, .28]^{C}	***	.09 [.07, .12]^{C}	***	.17 [.13, .20]^{C}	***	.31 [.28, .33]^{C}	***
**H15**	Education (planned)$^{w/o}$	Education bias	.49 [.43, .55]^{Cc}	***	.42 [.35, .49]^{Cc}	***	.50 [.42, .57]^{C}	***	.22 [.16, .28]^{Cc}	***
**H15**	Education (orthogonal)$^{w/o}$	Education bias	-.08 [-.14, -.03]	.003	-.09 [-.14, -.04]	***	-.07 [-.14, .01]	.087	-.08 [-.14, -.02]	.008
**H16**	Type of target group$^{w/o}$	Bias equivalence^{A}	-.19 [-.22, -.16]^{N}	***	missing	n/a	missing	n/a	-.45 [-.49, -.42]^{N}	***
**H17**	Education (planned)$^{w/o}$	Thematic/holistic	-.12 [-.18, -.06]^{C}	***	-.21 [-.28, -.14]^{C}	***	-.38 [-.46, -.30]^{C}	***	-.11 [-.17, -.05]^{C}	***
**H17**	Education (orthogonal)$^{w/o}$	Thematic/holistic	-.01 [-.06, .05]	.838	.02 [-.03, .07]	.543	.07 [-.01, .14]	.101	-.01 [-.07, .05]	.734
**H18**	Education (planned)$^{w/o}$	Anticipate change	-.02 [-.08, .04]^{N}	.488	-.36 [-.43, -.29]^{C}	***	-.60 [-.68, -.52]^{Cc}	***	-.31 [-.37, -.24]^{C}	***
**H18**	Education (orthogonal)$^{w/o}$	Anticipate change	-.05 [-.11, .00]	.072	.03 [-.02, .09]	.177	.10 [.03, .18]	.008	-.01 [-.07, .05]	.749
**H19**	Subjective SES^{w}	Contextual explan.	.06 [.03, .09]^{R}	***	.01 [-.02, .04]^{N}	.669	-.12 [-.16, -.07]^{C}	***	.22 [.19, .25]^{R}	***
**H19**	Subjective SES$^{w/o}$	Contextual explan.	.07 [.04, .09]^{R}	***	-.01 [-.03, .02]^{N}	.589	-.09 [-.12, -.05]^{C}	***	.21 [.18, .23]^{R}	***
**H20**	Indirect via sense of control	Contextual explan.	-.02 [-.03, -.01]$^{n/a}$	***	-.02 [-.03, -.02]$^{n/a}$	***	-.09 [-.10, -.07]^{C}	***	.04 [.03, .04]$^{n/a}$	***
**H21**	Income^{w}	Compassion (other)	-.02 [-.04, .00]^{Cm}	.079	.01 [-.01, .03]^{N}	.155	-.01 [-.04, .02]^{N}	.413	.01 [.00, .03]^{N}	.109
**H21**	Income$^{w/o}$	Compassion (other)	-.03 [-.05, -.01]^{C}	.009	.00 [-.02, .02]^{N}	.959	-.02 [-.05, .00]^{N}	.102	.01 [.00, .03]^{Rm}	.090
**H22**	Income^{w}	Love (other)	.04 [.02, .05]^{R}	***	.01 [-.01, .04]^{N}	.158	.00 [-.03, .03]^{N}	.960	.00 [-.01, .02]^{N}	.907
**H22**	Income$^{w/o}$	Love (other)	.03 [.01, .04]^{R}	.001	.03 [.01, .05]^{R}	.004	-.01 [-.04, .02]^{N}	.548	.00 [-.01, .02]^{N}	.622
**H23**	Income^{w}	Contentment (self)	.07 [.05, .09]^{C}	***	.06 [.03, .08]^{C}	***	.07 [.02, .12]^{C}	.008	-.02 [-.04, -.00]^{R}	.031
**H23**	Income$^{w/o}$	Contentment (self)	.09 [.07, .12]^{C}	***	.07 [.05, .10]^{C}	***	.11 [.06, .15]^{C}	***	-.02 [-.04, -.01]^{R}	.011
**H24**	Income^{w}	Pride (self)	.02 [.00, .04]^{C}	.033	.03 [.00, .05]^{C}	.023	.04 [.01, .08]^{C¶}	.011	.04 [.03, .05]^{C}	***
**H24**	Income$^{w/o}$	Pride (self)	.02 [.00, .04]^{C}	.028	.00 [-.02, .02]^{N}	.807	.02 [-.02, .05]^{N¶}	.382	.04 [.03, .05]^{C}	***
**H25**	Income × Sharing$^{w/o}$	Self-conscious T_1-T_2	-.09 [-.19, .02]^{N}	.107	-.05 [-.15, .06]^{N}	.371	-.03 [-.20, .13]^{N}	.688	-.02 [-.12, .09]^{N}	.729
**H25'**	Income × Sharing × Type$^{w/o}$	Emotion T_1-T_2	.06 [-.04, .15]^{N}	.233	.03 [-.04, .11]^{N}	.374	.09 [-.04, .23]^{N}	.183	.04 [-.06, .13]^{N}	.482

Table S7 - Primary Analysis. *Continued*

ID	Predictor	Outcome	USA *B* [95% CI]	USA *p*	France *B* [95% CI]	France *p*	Switzerland *B* [95% CI]	Switzerland *p*	India *B* [95% CI]	India *p*
**H25**	Educ^{planned} × Sharing$^{w/o}$	Self-conscious T_1-T_2	.04 [-.20, .28]^{N}	.763	.07 [-.19, .33]^{N}	.591	.12 [-.21, .46]^{N}	.472	-.01 [-.24, .21]^{N}	.901
**H25**	Educ$^{orthog.}$ × Sharing$^{w/o}$	Self-conscious T_1-T_2	-.07 [-.28, .14]	.517	-.02 [-.22, .18]	.843	.04 [-.29, .38]	.805	-.22 [-.45, -.002]	.047
**H25′**	Educ^{plan} × Sharing × Type$^{w/o}$	Emotion T_1-T_2	.14 [-.10, .38]^{N}	.247	.17 [-.09, .42]^{N}	.210	.08 [-.25, .41]^{N}	.623	.13 [-.10, .35]^{N}	.271
**H25′**	Educ^{orth} × Sharing × Type$^{w/o}$	Emotion T_1-T_2	.08 [-.13, .30]	.434	-.07 [-.27, .12]	.454	-.29 [-.62, .04]	.082	.02 [-.21, .25]	.869
**H26**	Subjective SES^{w}	Context. influence	.09 [.06, .12]^{R}	***	.04 [.01, .07]^{R}	.002	.01 [-.03, .05]^{N}	.726	.10 [.07, .12]^{R}	***
**H27**	Income × S/O benefits^{w}	Unethical behavior	-.03 [-.13, .07]^{N}	.552	.08 [-.03, .18]^{N}	.154	.01 [-.13, .14]^{N}	.906	-.05 [-.17, .08]^{N}	.478
**H27**	Educ^{planned} × S/O benefits^{w}	Unethical behavior	-.03 [-.27, .22]^{N}	.830	.22 [-.05, .49]^{N}	.115	-.05 [-.38, .28]^{N}	.767	.03 [-.22, .27]^{N}	.816
**H27**	Educ$^{orthog.}$ × S/O benefits^{w}	Unethical behavior	-.07 [-.28, .13]	.484	.00 [-.20, .20]	.988	-.02 [-.33, .30]	.912	-.14 [-.36, .08]	.221
**H27**	Income × S/O benefits$^{w/o}$	Unethical behavior	-.04 [-.15, .06]^{N}	.394	.07 [-.03, .18]^{N}	.160	.01 [-.14, .15]^{N}	.943	-.04 [-.17, .08]^{N}	.521
**H27**	Educ^{planned} × S/O benefits$^{w/o}$	Unethical behavior	-.04 [-.30, .22]^{N}	.775	.21 [-.06, .48]^{N}	.125	-.15 [-.48, .18]^{N}	.367	.04 [-.21, .28]^{N}	.776
**H27**	Educ$^{orthog.}$ × S/O benefits$^{w/o}$	Unethical behavior	-.10 [-.32, .12]	.356	-.02 [-.22, .18]	.866	.00 [-.33, .33]	.997	-.13 [-.35, .09]	.243
**H28**	Educ^{planned} × P/P context$^{w/o}$	Prosocial behavior	-.03 [-.16, .09]^{N}	.582	.05 [-.09, .18]^{N}	.483	.00 [-.17, .18]^{N}	.959	.04 [-.08, .15]^{N}	.524
**H28**	Educ$^{orthog.}$ × P/P context$^{w/o}$	Prosocial behavior	-.02 [-.13, .09]	.760	-.01 [-.11, .09]	.907	-.02 [-.19, .16]	.863	-.04 [-.15, .07]	.479
**H28**	Income × P/P context$^{w/o}$	Prosocial behavior	.00 [-.05, .06]^{N}	.853	.00 [-.03, .04]^{N}	.807	.04 [-.02, .09]^{N}	.209	.00 [-.07, .06]^{N}.	.901
**H29**	Subjective SES^{w}	Unethical behavior	-.03 [-.06, -.01]^{R}	.010	-.03 [-.06, -.01]^{R}	.009	-.03 [-.07, .00]^{Rm}	.074	-.12 [-.15, -.10]^{R}	***
**H30**	Childhood Subj. SES^{w}	Delayed reward	-.08 [-.14, -.02]^{R}	.005	-.05 [-.10, .01]^{N}	.120	.02 [-.06, .10]^{N}	.648	.18 [.11, .25]^{C}	***
**H30**	Childhood Subj. SES$^{w/o}$	Delayed reward	-.03 [-.08, .02]^{N}	.242	-.04 [-.09, .01]^{N}	.130	.02 [-.06, .09]^{N}	.641	.20 [.15, .25]^{C}	***
**H31**	Childhood Subj. SES^{w}	Risk aversion	-.06 [-.12, -.01]^{C}	.029	-.07 [-.13, -.01]^{C}	.019	-.02 [-.10, .05]^{N}	.545	-.07 [-.14, .00]^{C}	.041
**H31**	Childhood Subj. SES$^{w/o}$	Risk aversion	-.14 [-.19, -.09]^{C}	***	-.14 [-.20, -.09]^{C}	***	-.06 [-.13, .02]^{N}	.137	-.09 [-.14, -.04]^{C}	***
**H32**	Child. subj. × Mortality^{w}	Delayed reward	-.01 [-.11, .09]^{C}	.839	-.01 [-.11, .09]^{C}	.835	.04 [-.11, .19]^{N}	.629	.05 [-.05, .15]^{N}	.306
**H32**	Child. subj. × Mortality$^{w/o}$	Delayed reward	-.02 [-.12, .09]^{C}	.759	-.01 [-.11, .09]^{C}	.842	.04 [-.11, .19]^{N}	.629	.05 [-.05, .15]^{N}	.305
**H33**	Child. subj. × Mortality^{w}	Risk aversion	-.02 [-.12, .08]^{N}	.649	-.01 [-.11, .09]^{C}	.854	.10 [-.04, .25]^{N}	.166	.09 [-.01, .19]^{N}	.064
**H33**	Child. subj. × Mortality$^{w/o}$	Risk aversion	-.01 [-.12, .09]^{C}	.776	-.01 [-.12, .09]^{C}	.773	.10 [-.05, .25]^{N}	.190	.09 [-.01, .19]^{N}	.063
**H32**	Child. subj. × Mortality^{w,no}	Delayed reward	-.11 [-.23, .01]^{N}	.076	-.06 [-.18, .07]^{N}	.338	-.02 [-.18, .14]^{N}	.807	.09 [-.03, .21]^{N}	.159
**H32**	Child. subj. × Mortality$^{w/o,no}$	Delayed reward	-.12 [-.25, .00]^{N}	.058	-.06 [-.19, .07]^{N}	.344	-.02 [-.18, .14]^{N}	.807	.09 [-.03, .21]^{N}	.151
**H33**	Child. subj × Mortality^{w,no}	Risk aversion	-.04 [-.17, .08]^{N}	.489	-.00 [-.13, .12]^{C}	.957	.07 [-.08, .22]^{N}	.367	.07 [-.05, .19]^{N}	.275
**H33**	Child. subj × Mortality$^{w/o,no}$	Risk aversion	-.04 [-.12, .09]^{N}	.557	-.01 [-.14, .12]^{C}	.891	.06 [-.10, .22]^{N}	.466	.07 [-.06, .19]^{N}	.286
**H34**	Income × Problems$^{w/o}$	Performance	.09 [-.02, .20]^{N}	.103	.14 [-.02, .29]^{Cm}	.084	.14 [-.05, .32]^{N}	.147	-.01 [-.10, .08]^{N}	.857
**H34**	Income × Problems$^{w/o,no}$	Performance	.11 [-.02, .24]^{Cm}	.097	.25 [.09, .40]^{C}	.002	.14 [-.04, .32]^{N}	.116	-.00 [-.11, .11]^{N}	.993

Table S7. *Continued*

ID	Predictor	Outcome	USA *B* [95% CI]	USA *p*	France *B* [95% CI]	France *p*	Switzerland *B* [95% CI]	Switzerland *p*	India *B* [95% CI]	India *p*
**H35**	Perceived Resources[w]	Utilitarian decision	.51 [.41, .62][C]	***	missing	n/a	missing	n/a	.32 [.26, .37][C]	***
**H35**	Perceived Resources[w/o]	Utilitarian decision	.56 [.47, .65][C]	***	missing	n/a	missing	n/a	.33 [.27, .38][C]	***

Note. All predictors and outcomes were standardized, which means that coefficients can roughly be interpreted as correlations, with three exceptions: (i) when social class is operationalized using education – in that case, the coefficient captures the change from the lowest to the highest education group (planned) and from the middle education group to the two others (orthogonal); (ii) when an interaction is tested – in this case, the coding for dichotomous variables is aligned with the direction of the hypothesized effect (for **H12-13**, “chaos” was coded as +0.5 and “stability” as -0.5; for **H25-25′**, “nearly equal sharing” was coded as +0.5 and “low sharing” as -0.5; for **H27**, “self-beneficial” was coded as +0.5 and “other-beneficial” as -0.5; for **H32-33**, “dangerous world” was coded as +0.5 and “control” as -0.5; for **H34**, “hard problem” was coded as +0.5 and “easy problem” as -0.5); and (iii) when the outcome is binary – in this case, logistic rather than linear regression was used, and log-odds are reported (i.e., for **H9** and **H35**). Because income was right-skewed, we log-transformed the variable and used robust standard errors in analyses where it was used. To conserve space, the zero-unit digits of the *B*s are omitted.

Legend. ‘missing’ indicates a shortcoming in the questionnaire where a focal variable was not assessed, and ‘n/a’ means ‘not applicable’ (as the test was missing). ‘S/O’ means “Self/Other benefits” and ‘P/P’ means “Private/Public.” The prime symbol (′) indicates that the test was not performed in the original study, but we considered it a more appropriate test of the hypothesis. The pilcrow (¶) indicates tests where the outcome variable has a Cronbach's alpha of less than 0.60, meaning that the results should be interpreted with caution.

[A] For **H16**, the focal test is an equivalence test, meaning that *B* represents the standardized difference between education and ethnic/national bias, and the CI does not represent a 95% but a 90% CI.

Below are the meanings of the superscripts associated with the predictors:
[w] The analysis was performed with the same covariates as the original study,
[w/o] The analysis was performed without covariates, as in the original study
[no] The analysis was performed without rushers (i.e., participants who spent less than half the median time on the induction).

Below are the meanings of the superscripts associated with the inferential statistics:
[C] The outcome of the analysis is consistent with the original hypothesis.
[R] The outcome of the analysis is reversed compared to the original hypothesis.

[N] The outcome of the analysis is not consistent with the original hypothesis.

[Cm] The effect observed is consistent with the original hypothesis, but it is only marginally significant (eventually treated as 'N').

[Rm] The effect observed is reversed compared to the original hypothesis, but it is only marginally significant (eventually treated as 'N').

[Cc] Both contrast terms are significant, but the overall pattern remains consistent with the original hypothesis (eventually treated as 'C').

[Nc] Both contrast terms are significant and the overall pattern is inconsistent with the original hypothesis (eventually treated as 'N').

[Cp] The interaction effect is consistent with the original hypothesis, but the pattern of simple slopes is not the same (eventually treated as 'N').

[n/a] The indirect effect is reported for transparency, but is not interpreted, as the total effect is either inconsistent with the hypothesis, or reversed.

$^{***}p < .001$

Table S8 - Primary Analysis. *Conclusions on Whether Each Focal Estimate is Positive, Negative, Equivalent to Zero, or Nonsignificant*

ID	Hypothesis	USA	France	Switzerland	India
**H1**	Social class $^{+}\rightarrow$ Sense of control	positive	positive	positive	negative
**H2**	Social class $^{+}\rightarrow$ Agency	positive	positive	positive	positive
**H3**	Social class $^{+}\rightarrow$ Persistence in goal striving	positive	positive	positive	positive
**H4**	Social class $^{+}\rightarrow$ Self-esteem	positive	positive	positive	zero: .00 [-.02, .02]
**H2/4**	Social class $^{+}\rightarrow$ Omnibus self-orientation measure	positive	positive	positive	positive
**H5**	Social class $^{+}\rightarrow$ Narcissism	positive	positive	zero: .01 [-.02, .04]	positive
**H6**	Social class $^{+}\rightarrow$ Entitlement	positive	positive	negative	positive
**H7**	Social class × System-justification $^{+}\rightarrow$ Entitlement	positive	zero: .02 [.00, .04]	n.s.: .02 [-.01, .05]	negative
**H8**	Social class $^{-}\rightarrow$ Interdependent self-construal	n.s.: .04 [-.01, .10]	n.s.: .04 [-.02, .09]	n.s.: .07 [.00, .13]	negative
**H9**	Social class $^{+}\rightarrow$ Negative reactions to deindividuation	negative	n.s.: .00 [-.21, .22]	negative	negative
**H10**	Social class $^{-}\rightarrow$ Individuals in one's inner circle	negative	n.s.: -.11 [-.23, .01]	negative	positive
**H11**	Social class $^{-}\rightarrow$ Social support received	positive	n.s.: .16 [.02, .29]	n.s.: .15 [-.02, .32]	positive
**H12**	Social class × Stability/Chaos $^{-}\rightarrow$ Communal orientation	n.s.: -.02 [-.07, .02]	negative	n.s.: .04 [-.02, .10]	negative
**H13**	Social class × Stability/Chaos $^{+}\rightarrow$ Obsession with money	n.s.: -.01 [-.06, .03]	n.s.: .01 [-.03, .06]	n.s.: -.05 [-.11, .02]	negative
**H14**	Education bias	positive	positive	positive	positive
**H15**	Social class $^{+}\rightarrow$ Education bias	positive	positive	positive	positive
**H16**	Education bias = Ethnic/national bias	negative	n/a	n/a	negative
**H17**	Social class $^{-}\rightarrow$ Thematic/holistic thinking style	negative	negative	negative	negative
**H18**	Social class $^{-}\rightarrow$ Anticipation of change	n.s.: -.02 [-.07, .03]	negative	negative	negative
**H19**	Social class $^{-}\rightarrow$ Contextual explanations	positive	zero: -.01 [-.03, .01]	negative	positive
**H20**	Social class $^{+}\rightarrow$ Sense of control $^{-}\rightarrow$ contextual explanation	negative	n/a	negative	positive
**H21**	Social class $^{-}\rightarrow$ Compassion (other-oriented emotion)	negative	zero: .00 [-.02, .02]	zero: -.02 [-.05, .00]	zero: .01 [.00, .02]
**H22**	Social class $^{-}\rightarrow$ Love (other-oriented emotion)	positive	positive	zero: -.01 [-.04, .02]	zero: .00 [-.01, .02]
**H23**	Social class $^{+}\rightarrow$ Contentment (self-oriented emotion)	positive	positive	positive	negative
**H24**	Social class $^{+}\rightarrow$ Pride (self-oriented emotion)	positive	zero: .00 [-.02, .01]	zero: .02 [-.01, .05]	positive
**H25**	Social class × Sharing × Time $^{-}\rightarrow$ Self-conscious emotions	n.s.: .04 [-.17, .24]	n.s.: .07 [-.15, .29]	n.s.: .12 [-.16, .41]	n.s.: -.01 [-.20, .17]
**H25′**	Social class × Sharing × Time × Type $^{-}\rightarrow$ Negative emotions	n.s.: .14 [-.06, .34]	n.s.: .17 [-.05, .38]	n.s.: .08 [-.20, .36]	n.s.: .13 [-.06, .31]
**H26**	Social class $^{-}\rightarrow$ Influence of contextual information	positive	positive	zero: .01 [-.03, .04]	positive
**H27**	Social class × Self/Other benefits $^{+}\rightarrow$ Unethical behavior	n.s.: -.04 [-.13, .04]	n.s.: .07 [-.01, .16]	n.s.: .01 [-.12, .13]	n.s.: -.04 [-.15, .06]
**H28**	Social class × Private/Public $^{-}\rightarrow$ Prosocial behavior	n.s.: -.03 [-.14, .07]	n.s.: .05 [-.06, .16]	n.s.: .00 [-.14, .15]	n.s.: .04 [-.06, .13]

Table S8 - Primary Analysis. *Continued*

ID	Hypothesis	USA	France	Switzerland	India
**H29**	Social class $^{+}\rightarrow$ Unethical behaviors	negative	negative	n.s.: -.03 [-.06, .00]	negative
**H30**	Social class $^{+}\rightarrow$ Preference of delayed reward	n.s.: -.03 [-.07, .01]	n.s.: -.04 [-.08, .00]	n.s.: .02 [-.05, .08]	positive
**H31**	Social class $^{-}\rightarrow$ Risk aversion	negative	negative	n.s.: -.06 [-.12, .01]	negative
**H32**	Social class × Mortality/control $^{\emptyset}\rightarrow$ Delayed reward	n.s.: -.12 [-.23, -.02]	n.s.: -.06 [-.17, .05]	n.s.: -.02 [-.15, .11]	n.s.: .09 [-.01, .19]
**H33**	Social class × Mortality (vs. control) $^{\emptyset}\rightarrow$ Risk aversion	n.s.: -.04 [-.14, .07]	n.s.: -.01 [-.12, .10]	n.s.: .06 [-.07, .19]	n.s.: .07 [-.04, .17]
**H34**	Social class × Hard/Easy problems $^{+}\rightarrow$ Performance	n.s.: .11 [.00, .22]	positive	n.s.: .14 [-.01, .29]	n.s.: .00 [-.09, .10]
**H35**	Social class $^{+}\rightarrow$ Utilitarian moral decision-making	positive	n/a	n/a	positive

Note. This table identifies whether each focal estimate from **Table 2** in the main manuscript is significantly positive (‘positive’), significantly negative (‘negative’), nonsignificant and equivalent to zero (‘zero’), or nonsignificant but not equivalent to zero (‘n.s.’). For the latter two categories, the focal estimate and the 90% CI are provided. An estimate is deemed equivalent to zero if the upper equivalence bound (B = +0.5) and/or the equivalence bound (B = -0.5) is included in the 90% CI. ‘n/a’ means ‘not applicable.’ This designation is used when the variable was missing from the questionnaire (for **H16** and **H35**) or when the total effect, upon which the test was based, was nonsignificant (for **H20**).

Detailed Report of All Replication Analyses Undertaken

Preregistration can be found in the OSF page of the project

General Notes

- All analyses were independently carried out twice by two researchers to guarantee the accuracy of the results.
- All continuous focal variables (i.e., outcomes and focal predictors), were standardized. The coding scheme used for the focal categorical variables is detailed in each respective report.
- Income followed a right-skewed distribution, creating heteroscedasticity-related issues. Thus, we log-transformed the variable and, as preregistered, we used robust standard errors in the analyses where income was used either as a focal predictor or a covariate.
- For the analyses in which gender was used as a covariate, the 'other' option was replaced with a missing value due to the insufficient number of nonbinary participants.
- Ethnicity was available only in the US sample ("1 = White"; 0 = "Nonwhite"). In the French and Swiss samples, ethnicity was replaced by nationality (1 = "citizens"; 0 = "noncitizens"), while in the Indian sample, it was replaced by religious group (1 = "Hindu"; 0 = "others").
- As preregistered, we conducted preliminary analyses to test whether the order of presentation of the social class indicator (i.e. whether it was at the beginning or end of the questionnaire) interacted with the focal predictor. This was not applicable to education and income that were used to build quotas and always presented at the beginning of the questionnaire. We observed six cases where this interaction was significant; however, this never affected the conclusions of the analyses. Consequently, the order of presentation was not included in the final models.

The Self (Nine Hypotheses)

S1/2. H1. JPSP, 2009. Social class $^{+}\rightarrow$ Sense of control

Mostly consistent with the hypothesis (US = 2/2; FR = 2/2; CH = 2/2; IN = 0/2)

As preregistered, sense of control (mean score) was regressed on subjective SES with and without the covariates used in the original study (i.e., contrast-coded education, log equivalized income, and political orientation). As can be seen in **Table S7**, the effect of subjective SES was: (1) significantly positive in the US, French, and Swiss samples, and significantly negative (i.e., reversed) in the Indian sample, when including covariates; (2) significantly positive in the US, French, and Swiss samples, and significantly negative (i.e., reversed) in the Indian sample, when excluding covariates. In summary, the empirical evidence is mostly consistent with the hypothesis across samples and specifications.

S3. H2. JPSP, 2018. Social class $^{+}\rightarrow$ Agency

Mostly consistent with the hypothesis (US = 2/2; FR = 1/2; CH = 2/2; IN = 1/2)

As preregistered, agency (mean score) was regressed on subjective SES (first model) or contrast-coded education (second model) with the covariates used in the original study (i.e., gender and age). As can be seen in **Table S7**, the effect of social class was: (1) significantly positive in the US, French, Swiss, and Indian samples when using subjective SES as the predictor; (2) significantly positive in the US and Swiss samples, nonsignificant in the French sample, and significantly negative (i.e., reversed) in the Indian sample (both contrast terms

were significant, and the pattern was not linear, as the middle education group fell below the other two groups) when using contrast-coded education as the predictor. In summary, the empirical evidence is mostly consistent with the hypothesis across samples and specifications.

S3. H3. JPSP, 2018. Social class $^{+}\rightarrow$ Persistence in goal striving

Mostly consistent with the hypothesis (US = 2/2; FR = 2/2; CH = 2/2; IN = 1/2)

As preregistered, persistence in goal striving (mean score) was regressed on subjective SES (first model) or contrast-coded education (second model) with the covariates used in the original study (i.e., gender and age). As can be seen in **Table S7**, the effect of social class was: (1) significantly positive in the US, French, Swiss, and Indian samples when using subjective SES as the predictor; (2) significantly positive in the US, French, and Swiss samples, and inconsistent in the Indian sample (both contrast terms were significant, and the pattern was not linear, as the middle education group fell below the other two groups), when using contrast-coded education as the predictor. In summary, the empirical evidence is mostly consistent with the hypothesis across samples and specifications.

S3. H4. JPSP, 2018. Social class $^{+}\rightarrow$ Self-esteem

Mostly consistent with the hypothesis (US = 2/2; FR = 2/2; CH = 2/2; IN = 1/2)

As preregistered, for the Indian sample, we proceeded with the inferential analysis despite the low Cronbach's alpha (α = .34), as removing items did not improve the score. As preregistered, self-esteem (mean score) was regressed on subjective SES (first model) or contrast-coded education (second model) with the covariates used in the original study (i.e., gender and age). As can be seen in **Table S7**, the effect of social class was: (1) significantly positive in the US, French, and Swiss samples, and nonsignificant in the Indian sample, when using subjective SES as the predictor; (2) significantly positive in the US, French, Swiss, and Indian samples when using contrast-coded education as the predictor. In summary, the empirical evidence is mostly consistent with the hypothesis across samples and specifications.

S3. H2-4. JPSP, 2018. Social class $^{+}\rightarrow$ Omnibus self-orientation measure

Mostly consistent with the hypothesis (US = 2/2; FR = 2/2; CH = 2/2; IN = 1/2)

Agency, persistence in goal striving, and self-esteem were standardized and averaged to compute a self-orientation composite index. It was regressed on subjective SES (first model) or contrast-coded education (second model) with the covariates used in the original study (i.e., gender and age). As can be seen in **Table S7**, the effect of social class was: (1) significantly positive in the US, French, Swiss, and Indian samples when using subjective SES as the predictor; (2) significantly positive in the US, French, and Swiss samples, and inconsistent in the Indian sample (both contrast terms were significant, and the pattern was not linear, as the middle education group fell below the other two groups), when using contrast-coded education as the predictor. In summary, the empirical evidence is mostly consistent with the hypothesis across samples and specifications.

S4. H5. PLoS One, 2019. Social class $^{+}\rightarrow$ Narcissism

Mostly consistent with the hypothesis (US = 1/1; FR = 1/1; CH = 0/1; IN = 1/1)

As preregistered, narcissism (mean score) was regressed on subjective SES, with the

covariates used in the original study (i.e., gender and age). As can be seen in **Table S7**, the effect of subjective SES was significantly positive in the US, French, and Indian samples, but nonsignificant in the Swiss sample. In summary, the empirical evidence is mostly consistent with the hypothesis across samples.

S5/6. H6. PSPB, 2014. Social class $^{+}\rightarrow$ Entitlement

Mostly consistent with the hypothesis (US = 1/1; FR =1/1; CH = 0/1; IN = 1/1)

As preregistered, entitlement (mean score) was regressed on subjective SES, with the covariates used in the original study (i.e., gender and ethnicity). As can be seen in **Table S7**, the effect of subjective SES was significantly positive in the US, French, and Indian samples, and significantly negative (i.e., reversed) in the Swiss sample. In summary, the empirical evidence is mostly consistent with the hypothesis across samples.

S7. H7. JPSP, 2020. Social class × High (vs. low) system-justification $^{+}\rightarrow$ Entitlement

Rarely consistent with the hypothesis (US = 1/1; FR = 0/1; CH = 0/1; IN = 0/1)

As preregistered, entitlement (mean score) was regressed on occupation, system-justification beliefs, and their interaction. As can be seen in **Table S7**, the interaction effect was significantly positive in the US sample, nonsignificant in the French and Swiss samples, and significantly negative (i.e., reversed) in the Indian sample. The decomposition of the significant interactions revealed that the pattern of the simple slopes was consistent with the attenuated interaction hypothesis in the US (i.e., when system-justification beliefs are high, the higher the social class, the higher the sense of entitlement; when the system-justification beliefs are low, this relation is attenuated), and inconsistent in the Indian sample (for all simple slopes, see **Table S-S7**). In summary, the empirical evidence is rarely consistent with the hypothesis across samples.

Table S-S7. *Simple Effects of Social Class as a Function of System-Justification Beliefs.*

	US	FR	CH	IN
	B [95% CI]	*B* [95% CI]	*B* [95% CI]	*B* [95% CI]
Interaction	.07*** [.05, .09]	.02† [.00, .05]	.02 [-.01, .06]	-.02* [-.05, .00]
Low SJ (-1 *SD*)	-.06*** [-.09, -.03]	-.05*** [-.09, -.02]	-.12*** [-.17, -.07]	.09*** [.06, .13]
High SJ (+1 *SD*)	.08*** [.04, .11]	-.01 [-.05, .02]	-.08** [-.13, -.02]	.04** [.13, .08]

Note. 'SJ' means 'system justification.'

$^{***}p < .001$, $^{**}p < .01$, $^{*}p < .05$, †$p < .10$

S8a. H8. PNAS, 2010. Social class $^{-}\rightarrow$ Interdependent self-construal

Rarely consistent with the hypothesis (US = 0/1; FR = 0/1; CH = 0/1; IN = 1/1)

As preregistered, for the Swiss sample, we proceeded with the inferential analysis despite the low Cronbach's alpha (α = .58), as removing items did not improve the score. As preregistered, the difference between the mean ratings given to interdependent minus the mean ratings given to independent statements was regressed on contrast-coded education. As can be seen in **Table S7**, the effect of education was significantly negative in the Indian sample and nonsignificant in the US, French, and Swiss samples. In summary, the empirical evidence is rarely consistent with the hypothesis across samples.

S9. H9. JPSP, 2007. Social class $^{+}\rightarrow$ Negative reactions to reduced individuation

Never consistent with the hypothesis (US = 0/6; FR = 0/6; CH = 0/6; IN = 0/6)

As preregistered, each of the three responses to the close-ended questions (1 = "bad" and 0 = "good") was regressed (in three separate logistic models) on contrast-coded education, with and without the covariate used in the original study (i.e., ethnicity). As can be seen in **Table S7**, when including the covariate, the effect of education was (i) nonsignificant in the French sample for all items, (ii) significantly negative (i.e., reversed) in the US and Indian samples for all items and, (iii) nonsignificant for one item and significantly negative (i.e., reversed) for two items in the Swiss sample. Moreover, when excluding the covariate, the effect of education was (i) nonsignificant in the French sample for all items, (ii) significantly negative (i.e., reversed) in the US and Indian samples for all items and, (iii) nonsignificant for two items and significantly negative (i.e., reversed) for one item in the Swiss sample. In summary, the empirical evidence is never consistent with the hypothesis across samples and specifications.[1]

Relationships (Seven Hypotheses)

S8b. H10. PNAS, 2010. Social class $^{-}\rightarrow$ Individuals in one's inner circle

Mixed evidence (US = 1/1; FR = 0/1; CH = 1/1; IN = 0/1)

As preregistered, the ratio of network members in one's inner circle to the total number of network members in one's inner, middle, and outer circles was regressed on contrast-coded education. As can be seen in **Table S7**, the effect of education was significantly negative in the US and Swiss samples, nonsignificant in the French sample, and significantly positive (i.e., reversed) in the Indian Sample. In summary, the empirical evidence is mixed across samples.

S8b. H11. PNAS, 2010. Social class $^{-}\rightarrow$ Social support received

Never consistent with the hypothesis (US = 0/1; FR = 0/1; CH = 0/1; IN = 0/1)

As preregistered, the ratio of network members showing social support relative to the total number of network members showing support and annoyances was regressed on contrast-coded education. As can be seen in **Table S7**, the effect of education was nonsignificant in the French and Swiss samples and significantly positive (i.e., reversed) in the US and Indian samples. In summary, the empirical evidence is never consistent with the hypothesis across samples.

S10. H12. JPSP, 2012. Social class × Chaos (vs. stability) $^{-}\rightarrow$ Communal orientation

Never consistent with the hypothesis (US = 0/1; FR = 0/1; CH = 0/1; IN = 0/1)

As preregistered, communal orientation (mean score) was regressed on log equivalized income, expectation of one's future economic well-being (-0.5 = "stable"; +0.5 = "chaotic"), and their interaction, with the covariates used in the original study (i.e., ethnicity and its interaction with expectation of chaos). As can be seen in **Table S7**, the interaction effect was significantly negative in the French and Indian samples and nonsignificant in the US and

[1] To calculate the mean effect across items reported in **Table 2**, we used a two-level multilevel logistic model where item-level observations were nested within participants. We regressed responses to the items on contrast-coded education while including ethnicity from the original study as a covariate.

Swiss samples. The decomposition of the significant interactions revealed that the pattern was inconsistent with the attenuated interaction hypothesis in the French and Indian samples (i.e, among participants expecting future chaos, the higher the social class, the lower the communal orientation; for participants expecting stability, this relation is attenuated; for all simple slopes, see **Table S-S10**). In summary, the empirical evidence is never consistent with the hypothesis across samples.

Table S-S10. *Simple Effects of Social Class as a Function of Expectation of Chaos*

	US	FR	CH	IN
	B [95% CI]	*B* [95% CI]	*B* [95% CI]	*B* [95% CI]
Interaction	-.02 [-.08, .03]	-.06* [-.11, -.01]	.04 [-.03, .12]	-.17*** [-.21, -.12]
Stable	.07** [.03, .11]	.09*** [.05, .12]	-.08*** [-.12, -.04]	.17*** [.14, .21]
Chaotic	.04* [.01, .08]	.02 [-.01, .06]	-.04 [-.10, .02]	.00 [-.02,- .04]

***$p < .001$, **$p < .01$, *$p < .05$, †$p < .10$

S11. H13. JPSP, 2012. Social class × Chaos (vs. stability) +→ Obsession with money

Never consistent with the hypothesis (US = 0/1; FR = 0/1; CH = 0/1; IN = 0/1)

As preregistered, obsession for money (mean score) was regressed on subjective SES, expectation of one's future economic well-being (-0.5 = "stable" and +0.5 = "chaotic"), and their interaction, with the covariates of the original studies (ethnicity and its interaction with expectation of chaos). As can be seen in **Table S7**, the interaction was nonsignificant in the US, French, and Swiss samples and was significantly negative (i.e., reversed) in the Indian sample. The decomposition of the significant interaction revealed that the pattern of the simple slopes was inconsistent with the attenuated interaction hypothesis in the Indian sample (i.e., among participants expecting future chaos, the higher the social class, the higher the obsession for money; for participants expecting stability, this relation is attenuated; for all simple slopes, see **Table S-S11**). In summary, the empirical evidence is never consistent with the hypothesis across samples.

Table S-S11. *Simple Effects of Social Class as a function of Chaos (vs. stability).*

	US	FR	CH	IN
	B [95% CI]	*B* [95% CI]	*B* [95% CI]	*B* [95% CI]
Interaction	-.01 [-.06, .04]	.01 [-.04, .06]	-.05 [-.13, .03]	-.12*** [-.17, -.07]
Stability	.29*** [.26, .33]	.19*** [.15, .23]	.04 [-.05, .04]	.23*** [.20, .27]
Chaos	.28*** [.24, .32]	.20*** [.17, .24]	-.05† [-.11, .01]	.11*** [.08, .15]

***$p < .001$, **$p < .01$, *$p < .05$, †$p < .10$

S12. H14. JESP, 2018. Education bias (highly educated people rated higher)

Always consistent with the hypothesis (US = 1/1; FR =1/1; CH = 1/1; IN = 1/1)

As preregistered, we performed a paired sample *t*-test to compare the thermometer ratings of the higher and lower-educated groups. As can be seen in **Table S7**, the effect is significantly positive in the US, French, Swiss, and Indian samples. In summary, the empirical evidence is always consistent with the hypothesis across samples.

S12. H15. JESP, 2018. Social class $^{+}$→ Education bias

Always consistent with the hypothesis (US = 1/1; FR =1/1; CH = 1/1; IN = 1/1)

As preregistered, the difference in thermometer ratings between the higher-educated group and the lower-educated group was regressed on contrast-coded education. As can be seen in **Table S7**, the effect is significantly positive in the US, French, Swiss, and Indian samples. In summary, the empirical evidence is always consistent with the hypothesis across samples.

S12. H16. JESP, 2018. Education bias = Ethnic/national bias

Never consistent with the hypothesis (US = 0/1; FR = missing; CH = missing; IN = 0/1)

This analysis deviated from the preregistration due to a shortcoming during the translation phase. This issue was not identified during the review process, resulting in the misconfiguration of the survey. Specifically, the ethnic/racial groups presented to participants did not include the national ingroup for the French and Swiss samples. Consequently, we did not perform the analyses for these samples. However, the error was corrected during data collection, enabling us to carry out the analyses for the U.S. and Indian samples.

We computed the ethnic/national bias by first averaging the four ethnic/national outgroup ratings, and then calculating the difference between the thermometer rating of the ethnic/national ingroup minus this average outgroup rating. As preregistered, we performed an equivalence test to compare the education bias and the ethnic/national bias, while setting our lower/upper equivalence bounds at d = -0.20/+0.20 (i.e., smallest negative effect size of interest). As can be seen in **Table S7**, the 90% confidence intervals fell within these lower and upper equivalence bounds (rejecting the null hypothesis of nonequivalence), and the education bias was significantly smaller than the ethnic/national bias (rejecting the null hypothesis of no difference) in the US and Indian samples. In summary, the empirical evidence is never consistent with the hypothesis across the two samples.

Cognition (Four Hypotheses)

S8c. H17. PNAS, 2010. Social class $^{-}$→ Thematic/holistic thinking style

Always consistent with the hypothesis (US = 1/1; FR =1/1; CH = 1/1; IN = 1/1)

As preregistered, the average proportion of responses related to thematic/holistic categorization (coded 1) versus taxonomic/analytical thinking (coded 0) was regressed on contrast-coded education. As can be seen in **Table S7**, the effect of education was significantly negative in the US, French, Swiss, and Indian samples. In summary, the empirical evidence is always consistent with the hypothesis across samples.

S8d. H18. PNAS, 2010. Social class $^{-}$→ Anticipation of change

Mostly consistent with the hypothesis (US = 0/1; FR =1/1; CH = 1/1; IN = 1/1)

As preregistered, the mean percentage of predicted change in life trajectory was regressed on contrast-coded education. As can be seen in **Table S7**, the effect of education was significantly negative in the French, Swiss, and Indian samples, and nonsignificant in the US sample. In summary, the empirical evidence is mostly consistent with the hypothesis across samples.

S13. H19. JPSP, 2009. Social class $^{-}$→ Contextual explanations

Rarely consistent with the hypothesis (US = 0/2 ; FR = 0/2; CH = 2/2; IN = 0/2)

As preregistered, contextual explanations (mean score) was regressed on subjective SES, with and without the covariates used in the original study (i.e., contrast-coded education, log equivalized income, and political orientation). As can be seen in **Table S7**, the effect of subjective SES was: (1) significantly negative in the Swiss sample, nonsignificant in the French sample and significantly positive (i.e., reversed) in the US and Indian samples, when including covariates; (2) significantly negative in the Swiss sample, nonsignificant in the French sample and significantly positive (i.e., reversed) in the US and Indian samples, when excluding covariates. In summary, the empirical evidence is rarely consistent with the hypothesis across samples and specifications.

S13. H20. JPSP, 2009. Social class $^{+}$→ Sense of control $^{-}$→ Contextual explanations
Rarely consistent with the hypothesis (US = n/a ; FR =n/a; CH = 1/1; IN = n/a)
As preregistered, we performed mediation analyses using percentile bootstrap confidence intervals with contextual explanations (mean score) as the outcome, subjective SES as the predictor, and sense of control (mean score) as the mediator, with the covariates used in the original study (i.e., contrast-coded education, log equivalized income, and political orientation). All indirect effects are reported in **Table S7** for transparency. However, given that our preregistered hypothesis and analysis focus on the mediation of the negative effect of social class on contextual explanations by sense of control, we only interpret the indirect effect in cases where the total effect of social class on contextual explanations is consistent with the hypothesis (i.e., the Swiss sample). As can be seen in **Table S7**, the indirect effect is significant and negative in the Swiss sample. In summary, the empirical evidence is rarely consistent with the hypothesis across samples.

Emotions (Six Hypotheses)

S14. H21. Emotion, 2018. Social class $^{-}$→ Compassion (Other-oriented positive emotions)
Rarely consistent with the hypothesis (US = 1/2 ; FR = 0/2; CH = 0/2; IN = 0/2)
As preregistered, compassion (mean score) was regressed on log equivalized income while controlling for the other three emotions, with and without the covariates used in the original study (i.e., gender, age, ethnicity, religiosity and political orientation). As can be seen in **Table S7**, the effect of income was: (1) nonsignificant in the US, French, Swiss, and Indian samples when including covariates; (2) significantly negative in the US sample, and nonsignificant in the French, Swiss, and Indian samples, when excluding covariates. In summary, the empirical evidence is rarely consistent with the hypothesis across samples and specifications.

S14. H22. Emotion, 2018. Social class $^{-}$→ Love (Other-oriented positive emotions)
Never consistent with the hypothesis (US = 0/2 ; FR = 0/2; CH = 0/2; IN = 0/2)
As preregistered, love (mean score) was regressed on log equivalized income while controlling for the other three emotions, with and without the covariates used in the original study (i.e., gender, age, ethnicity, religiosity and political orientation). As can be seen in **Table S7**, the effect of income was: (1) nonsignificant in the French, Swiss, and Indian samples, and significantly positive (i.e., reversed) in the US sample, when including covariates; (2) nonsignificant in the Swiss and Indian samples, and significantly positive (i.e.,

reversed) in the US and French samples, when excluding covariates. In summary, the empirical evidence is never consistent with the hypothesis across samples and specifications.

S14. H23. Emotion, 2018. Social class $^{+}$→ Contentment (Self-oriented positive emotions)
Mostly consistent with the hypothesis (US = 2/2 ; FR =2/2; CH = 2/2; IN = 0/2)
As preregistered, contentment (mean score) was regressed on log equivalized income while controlling for the other three emotions, with and without the covariates used in the original study (i.e., gender, age, ethnicity, religiosity and political orientation). As can be seen in **Table S7**, the effect of income was: (1) significantly positive in the US, French, and Swiss samples, and significantly negative (i.e., reversed) in the Indian sample, when including covariates; (2) significantly positive in the US, French, and Swiss samples, and significantly negative (i.e., reversed) in the Indian sample, when excluding covariates. In summary, the empirical evidence is mostly consistent with the hypothesis across samples and specifications.

S14. H24. Emotion, 2018. Social class $^{+}$→ Pride (Self-oriented positive emotions)
Mostly consistent with the hypothesis (US = 2/2 ; FR =1/2; CH = 1/2; IN = 2/2)
As preregistered, for the Swiss sample, we proceeded with the inferential analysis despite the low Cronbach's alpha (α = .56), as removing items did not improve the score. As preregistered, pride (mean score) was regressed on log equivalized income while controlling for the other three emotions, with and without the covariates used in the original study (i.e., gender, age, ethnicity, religiosity and political orientation). As can be seen in **Table S7**, the effect of income was: (1) significantly positive in the US, French, Swiss, and Indian samples when including covariates; (2) significantly positive in the US and Indian samples, and nonsignificant in the French and Swiss samples, when excluding covariates. In summary, the empirical evidence is mostly consistent with the hypothesis across samples and specifications.

E1. H25. Frontiers, 2014. Social class × Sharing × Time $^{-}$→ Self-conscious emotions
Never consistent with the hypothesis (US = 0/2 ; FR = 0/2; CH = 0/2; IN = 0/2)
As preregistered, the difference between the baseline self-conscious negative emotions minus the post-manipulation self-conscious negative emotions was regressed on the sharing condition (-0.5 = "low sharing"; +0.5 = "nearly equal sharing"), contrast-coded education (first model) or log equivalized income (second model), and their interaction. As can be seen in **Table S7**, the interaction effect was (1) nonsignificant in the US, French, Swiss, and Indian samples when using income as the predictor; (2) nonsignificant in the US, French, Swiss, and Indian samples when using education as the predictor. In summary, the empirical evidence is never consistent with the hypothesis across samples and specifications.

E1. H25'. Frontiers. 2014. Social class × Sharing × Time × Type emotion $^{-}$→ Neg. emotions
Never consistent with the hypothesis (US = 0/2 ; FR = 0/2; CH = 0/2; IN = 0/2)
The authors of the original article observed that the social class × sharing × time interaction was not significant for other types of non-self-conscious negative emotions. While they did not conduct a formal test of the higher-order interaction involving the type of emotions, they concluded that "effects on emotion ratings following the Dictator game appear to be localized to negative self-conscious affect" (p. 6). For our part, we preregistered a formal test of the

social class × sharing × time × type of emotions interaction, but it should be noted that this test was not taken into account to compute the replication rates.

As preregistered, we tested the interaction between the type of emotion (negative self-conscious emotion vs. non-self-conscious negative emotion), time (baseline vs. post-manipulation), the sharing condition (-0.5 = "low sharing" and +0.5 = "nearly equal sharing"), and the contrast-coded education (first model) or log equivalized income (second model). As can be seen in **Table S7**, the higher-order interaction effect was (1) nonsignificant in the US, French, Swiss, and Indian samples when using income as the predictor; (2) nonsignificant in the US, French, Swiss, and Indian samples when using education as the predictor. In summary, the empirical evidence is never consistent with the hypothesis across samples and specifications.

S15. H26. JPSP, 2009. Social class $^{-}\rightarrow$ Influence of contextual emotional information
Never consistent with the hypothesis (US = 0/1 ; FR = 0/1; CH = 0/1; IN = 0/1)
As preregistered, we created an index of contextual influence on emotion ratings by calculating the standard deviation of the ratings for all the emotions corresponding to the emotion expressed by the focal person (i.e., ignoring the filler emotion ratings). The index of contextual influence on emotion ratings was regressed on subjective SES, with the covariates used in the original study (i.e., ethnicity, log equivalized income, and contrast-coded education). As can be seen in **Table S7**, the effect of subjective SES was nonsignificant in the Swiss sample, and significantly positive (i.e., reversed) in the US, French, and Indian samples. In summary, the empirical evidence is never consistent with the hypothesis across samples.

Behavior (Three Hypotheses)

E2. H27. JPSP, 2015. Social class × Self- (vs. other-) benefits $^{-}\rightarrow$ Unethical behaviors
Never consistent with the hypothesis (US = 0/4 ; FR = 0/4; CH = 0/4; IN = 0/4)
As preregistered, the propensity to engage in unethical behavior (mean score) was regressed on the self-/other-benefit condition (-0.5 = "self-beneficial"; -0.5 = "other-beneficial"), log equivalised income, education (contrast-coded), the income × condition interaction, and the education × condition interaction, with and without the covariates used in the original study (i.e., gender, age). As can be seen in **Table S7**, the higher-order interaction effect was (1) nonsignificant in the US, French, Swiss, and Indian samples when using income as the predictor, both when including or excluding the covariates; (2) nonsignificant in the US, French, Swiss, and Indian samples when using education as the predictor, both when including or excluding the covariates. In summary, the empirical evidence is never consistent with the hypothesis across samples and specifications.

E3. H28. SPPS, 2016. Social class × Private (vs. public) context $^{-}\rightarrow$ Prosocial behavior
Never consistent with the hypothesis (US = 0/2 ; FR = 0/2; CH = 0/2; IN = 0/2)
In a preliminary analysis, we tested whether the order of presentation of the within-participants condition interacted with the focal variable in predicting prosocial behavior for each country and each indicator. These interactions were never significant, and therefore, we did not consider this term in the final analyses.

As preregistered, the difference between the number of ticket donated in the private context condition minus the number of ticket donated in the public context condition was regressed on contrast-coded education (first model) and log equivalised income (second model). As can be seen in **Table S7**, the effect was (1) nonsignificant in the US, French, Swiss, and Indian samples when using education as the predictor; (2) nonsignificant in the US, French, Swiss, and Indian samples when using income as the predictor. In summary, the empirical evidence is never consistent with the hypothesis across samples and specifications.

S16. H29. PNAS, 2012. Social class $^{+}$→ Unethical behaviors

Never consistent with the hypothesis (US = 0/1 ; FR = 0/1; CH = 0/1; IN = 0/1)

As preregistered, the percentage chance one would hide the truth was regressed on subjective SES, with the covariates used in the original study (i.e., gender, ethnicity, age, religiosity and political orientation). As can be seen in **Table S7**, the effect was nonsignificant in the Swiss sample, and significantly negative (i.e., reversed) in the US, French, and Indian samples. In summary, the empirical evidence is never consistent with the hypothesis across samples.

Decision-Making (Six Hypotheses)

E4. H30. JESP, 2018. Social class $^{+}$→ Preference of delayed reward

Rarely consistent with the hypothesis (US = 0/2 ; FR = 0/2; CH = 0/2; IN = 2/2)

As preregistered, the preference for delayed rewards (mean score) was regressed on childhood subjective SES (i.e., childhood MacArthur Scale), the mortality induction (-0.5 = “control”; +0.5 = “dangerous world”), and their interaction, with and without a covariate (i.e., current subjective SES). As can be seen in **Table S7**, the effect of childhood SES was: (1) significantly positive in the Indian sample, nonsignificant in the French and Swiss samples, and significantly negative (i.e., reversed) in the US sample, when including the covariate; (2) significantly positive in the Indian sample, and nonsignificant in the US, French, Swiss samples, when excluding the covariate. In summary, the empirical evidence is rarely consistent with the hypothesis across samples and specifications.

E4. H31. JESP, 2018. Social class $^{-}$→ Risk aversion

Mostly consistent with the hypothesis (US = 2/2 ; FR = 2/2; CH = 0/2; IN = 2/2)

As preregistered, risk aversion (mean score) was regressed on childhood subjective SES (i.e., childhood MacArthur Scale), the mortality induction (-0.5 = “control”; +0.5 = “dangerous world”), and their interaction, with and without a covariate (i.e., current subjective SES). As can be seen in **Table S7**, the effect of childhood SES was: (1) significantly negative in the US, French, and Indian samples, and nonsignificant in the Swiss samples, when including the covariate; (2) significantly negative in the US, French, and Indian samples, and nonsignificant in the Swiss samples, when excluding the covariate. In summary, the empirical evidence is mostly consistent with the hypothesis across samples and specifications.

E4. H32. JESP, 2018. Social class × Mortality (vs. control) $^{\emptyset}$→ Delayed reward

Rarely consistent with the hypothesis (US = 2/4 ; FR = 2/4; CH = 0/4; IN = 0/4)

As preregistered, we conducted all analyses both including and excluding participants identified as rushing, defined as those who read the induction in less than half of the median

time (med_{US} = 67.29, med_{FR} = 52.77, med_{CH} = 140.15, med_{IN} = 6.54 sec.[2]). Note that, deviating from our preregistration, we proceeded with the analysis excluding participants, even though this resulted in the power falling below .95 to detect a small-sized effect (n_{US} = 972 [*power* = .88], n_{FR} = 945 [*power* = .87], n_{CH} = 613 [*power* = .70], n_{IN} = 1,031 [*power* = .90]).[3]

As preregistered, the preference for delayed options (mean score) was regressed on childhood subjective SES (i.e., Childhood MacArthur Scale), the mortality induction (-0.5 = "control"; +0.5 = "dangerous world"), and their interaction, with and without a covariate (i.e., current subjective SES). As can be seen in **Table S7**, the interaction effect was (1) nonsignificant in the US, French, Swiss, and Indian samples when keeping the rushers, both when including or excluding the covariates; (2) nonsignificant in the US, French, Swiss, and Indian samples when removing the rushers, both when including or excluding the covariates

Also as preregistered, we performed equivalence tests to determine if the interaction between childhood SES and mortality induction was equivalent to zero, while setting the lower/upper equivalence bound at f = -0.05/0.05 (i.e., smallest negative effect size of interest). The analyses revealed that the interaction effect was: (1) equivalent to zero in the US and French samples, and not equivalent to zero in the Swiss and Indian samples, when keeping the rushers, both when including or excluding the covariates; (2) not equivalent to zero in the US, French, Swiss, and Indian when removing the rushers, both when including or excluding the covariates. In summary, the empirical evidence is rarely consistent with the hypothesis across samples and specifications.

E4. H33. JESP, 2018. Social class × Mortality (vs. control) $^{\varnothing}\rightarrow$ Risk aversion

Rarely consistent with the hypothesis (US = 1/4 ; FR = 4/4; CH = 0/4; IN = 0/4)

As preregistered, we conducted all analyses both including and excluding participants identified as rushing, defined as those who read the induction in less than half of the median time (med_{US} = 67.29, med_{FR} = 52.77, med_{CH} = 140.15, med_{IN} = 6.54 sec.). Note that, deviating from our preregistration, we proceeded with the analysis excluding participants, even though this resulted in the power falling below .95 to detect a small-sized effect (n_{US} = 972 [*power* = .88], n_{FR} = 945 [*power* = .87], n_{CH} = 615 [*power* = .70], n_{IN} = 1,031 [*power* = .90]).[3]

As preregistered, risk aversion (mean score) was regressed on childhood subjective SES (i.e., Childhood MacArthur Scale), the mortality induction (-0.5 = "control"; +0.5 = "dangerous world"), and their interaction, with and without a covariate (i.e., current subjective SES). As can be seen in **Table S7**, the interaction effect was (1) nonsignificant in the US, French, Swiss, and Indian samples when keeping the rushers, both when including or excluding the covariates; (2) nonsignificant in the US, French, Swiss, and Indian samples when removing the rushers, both when including or excluding the covariates.

Also as preregistered, we performed equivalence tests to determine if the interaction between childhood SES and mortality induction was equivalent to zero, while setting the our lower/upper equivalence bound at f = -0.05/0.05 (i.e., smallest negative effect size of interest). The analyses revealed that the interaction effect was: (1) equivalent to zero in the

[2] We recommend exercising caution when interpreting the results of this analysis in India, due to the limited time spent reading the induction.

[3] We believe that, although statistical power is lower than expected, excluding rushers reduces noise and improve reliability.

French sample, but not equivalent to zero in the US, Swiss, and Indian samples when keeping the rushers and including the covariates; (2) equivalent to zero in the US and French samples, but not equivalent to zero in the Swiss and Indian samples when keeping the rushers and excluding the covariates; (3) equivalent to zero in the French sample, but not equivalent to zero in the US, Swiss, and Indian samples when removing the rushers and including the covariates; (4) equivalent to zero in the French sample, but not equivalent to zero in the US, Swiss, and Indian samples when removing the rushers and excluding the covariates. In summary, the empirical evidence is rarely consistent with the hypothesis across samples and specifications.

E5. H34. Science, 2013. Social class × Hard (vs. easy) financial problems $^{+}\rightarrow$ Performance
Rarely consistent with the hypothesis (US = 0/2 ; FR =1/2; CH = 0/2; IN = 0/2)
As preregistered, we took advantage of the soft launch of the studies by Qualtrics in the US, France, and India as an opportunity to conduct a series of pilot studies. These studies pre-tested six Raven's Standard Progressive Matrices, classified in the manual as being of moderate difficulty (Raven et al., 2000). In **Table S-E5a**, we present the proportion of correct answers and the confidence interval for each country. As preregistered, we selected matrices that were correctly solved by around 50% of participants: (1) Matrices C9, C8, and C10 for the US, France, and Switzerland, (2) C6, C8, and C10 for India.

Table S-E5a. *Proportion of Correct Responses by Country for Each Pre-Tested Matrix*

	US			France			Switzerland			India		
Matrix	*n*	%	95% CI	*n*	%	95% CI	*n*	%	95% CI	*n*	%	95% CI
C5	94	.79	[.70, .87]	192	.81	[.75, .86]	94	.79	[.70, .87]	118	.91	[.91, .85]
C6	93	.63	[.53, .73]	193	.63	[.56, .70]	93	.63	[.53, .73]	118	.71	[.71, .63]
C7	92	.74	[.64, .83]	193	.80	[.75,.86]	92	.74	[.65, .83]	118	.77	[.77, .69]
C8	94	.43	[.32, .53]	191	.46	[.38, .53]	94	.43	[.32, .53]	118	.51	[.51, .42]
C9	93	.63	[.53, .73]	193	.61	[.54,.68]	93	.63	[.53, .73]	118	.78	[.78, 0.7]
C10	93	.35	[.26, .45]	193	.44	[.37, .51]	93	.35	[.26, .45]	118	.48	[.48, .39]

Note. '%' designates the proportion of correct answers.

As preregistered, we conducted all analyses both including and excluding participants identified as rushing, defined as those who read the induction in less than half of the median time (med_{US} = 60.88; med_{FR} = 59.99; med_{CH} = 100.88; med_{IN} = 13.85[4]). Note that, deviating from our preregistration, we proceeded with the analysis excluding participants, even though this resulted in the power falling below .95 to detect a small-sized effect (n_{US} = 990 [*power* = .88], n_{FR} = 1,091 [*power* = .91], n_{CH} = 685 [*power* = .74], n_{IN} = 1,093 [*power* = .91]).[3]

As preregistered, performance (mean score) was regressed on log equivalized income income, the type of financial problems (-0.5 = "easy"; +0.5 = "hard"), and their interaction. As can be seen in **Table S7**, the interaction effect was (1) nonsignificant in the US, French, Swiss, and Indian samples when keeping the rushers; (2) significantly positive in the French sample and nonsignificant in the US, Swiss, and Indian samples when removing the rushers. The decomposition of the significant interaction revealed that the pattern of the simple slopes

[4] We recommend exercising caution when interpreting the results of this analysis in India, due to the limited time spent reading the induction.

was consistent with the attenuated interaction hypothesis in the French sample (i.e., when participants have to think about hard financial problems, the higher the social class, the higher the cognitive performance; when they have to think about easy financial problems, this relation is attenuated; for all simple slopes see **Table S2-E5b**). In summary, the empirical evidence is rarely consistent with the hypothesis across samples.

Table S-E5b. *Simple Effects of Social Class as a function of Hard (vs.Easy) Problems*

	US	FR	CH	IN
	B [95% CI]	*B* [95% CI]	*B* [95% CI]	*B* [95% CI]
Interaction (keeping rushers)	.09 [-.02, .20]	.14†[-.02, .29]	.14 [-.05, .32]	.01 [-.10, .08]
Easy	.10** [.03, .18]	.07* [.01, .15]	.08 [-.05, .22]	.06* [.00, .12]
Hard	.20*** [.12, .27]	.21*** [.08, .35]	.22*** [.10, .34]	.06† [-.00, .12]
Interaction (removing rushers)	.11† [-.02, .24]	.24** [.09, .40]	.14 [-.04, .32]	.00 [-.11, .11]
Easy	.07 [-.02, .17]	.01 [-.09, .11]	.07 [-.06, .19]	.09* [.01, .18]
Hard	.18*** [.09, .27]	.25*** [.14, .37]	.21*** [.08, .34]	.10** [-.02, .17]

***$p < .001$, **$p < .01$, *$p < .05$, †$p < .10$

S17. H35. JPSP, 2013. Social class ⁺→ Utilitarian moral decision-making

Always consistent with the hypothesis (US = 2/2; FR = missing; CH = missing; IN = 2/2)

The present analysis deviated from our pre-registration due to an oversight that occurred during the French and Swiss data collection. Specifically, the block containing the perceived availability of resources scale was inadvertently omitted from the French and Swiss questionnaires. However, this error was rectified during the course of the research, enabling us to proceed with the analyses for the US and Indian samples.

As preregistered, we performed a logistic regression in which the odds of pushing the stranger onto the track (0 = "no, it is not appropriate"; 1 = "yes, it is appropriate") was modeled as a function of perceived resources availability (mean score), with and without the covariates used in the original study (gender, age, ethnicity, religiosity and political orientation). As can be seen in **Table S7**, the effect of perceived resources availability was significantly positive in the US and Indian samples, when including and excluding the covariates. In summary, the empirical evidence is always consistent with the hypothesis across the two samples and specifications.

Calculation of the MDE with 95% Power In Figure 1

To calculate the minimum detectable effect, we used the following formula:

$$d = \frac{2 \times Z_{1-\beta=.95} + Z_{1-\alpha/2}}{\sqrt{n}} \quad (1)$$

…where d is the MDE expressed in Cohen's d, $Z_{1\text{-}\beta=.95}$ is the critical Z value associated 95% power (i.e., 1.6449), $Z_{1\text{-}\alpha/2}$ is the critical Z value associated a two-tailed test with $\alpha = .05$ (i.e., 1.96), and n is the analytical sample size (see Sommet et al., 2023).

Then, to convert Cohen's d into partial eta squared, we used the following formula:

$$\eta_p^2 = \frac{d^2}{d^2 + 4} \quad (2)$$

For **H20**, we converted standardized indirect effect into pseudo-partial eta using this formula

Given that $$r = \sqrt{\eta_p^2} \quad (3a)$$

And that $$|r| \approx \sqrt{|\beta_a \times \beta_b|} \quad (3b)$$

Then, $$\eta_p^2 \approx \sqrt{|\beta_a \times \beta_b|}^{2\times2} \quad (3c)$$

…where r is a correlation coefficient, β_a is the standardized estimate for path a, and β_b is the standardized estimate for path b.

For **H9** and **H35**, we converted the log-odds ratio into Cohen's d using this formula:

$$d = \frac{ln(OR) \times \sqrt{3}}{\pi} \quad (4)$$

…where $ln(OR)$ is the log-odds ratio (see Borenstein and Hedges's [2019] Eq. 1.76)

Table S9 - Primary Analysis. Minimum Detectable Effect Size (MDE, 95% Power) and Partial Eta Squared for Each Hypothesis and Country

ID	Hypothesis	MDE with 90% power (in η_p^2)				Partial eta squared (η_p^2)			
		USA	FR	CHE	India	USA	France	CHE	India
**H1**	Social class $^{+}\rightarrow$ Sense of control	.002	.002	.004	.002	.005	.012	.114	.004
**H2**	Social class $^{+}\rightarrow$ Agency	.002	.002	.004	.002	.101	.023	.024	.052
**H3**	Social class $^{+}\rightarrow$ Persistence in goal striving	.002	.002	.004	.002	.034	.011	.018	.005
**H4**	Social class $^{+}\rightarrow$ Self-esteem	.002	.002	.004	.002	.065	.015	.053	<.001
**H2/4**	Social class $^{+}\rightarrow$ Omnibus self-orientation measure	.002	.002	.004	.002	.107	.026	.048	.020
**H5**	Social class $^{+}\rightarrow$ Narcissism	.002	.002	.004	.002	.078	.028	<.001	.021
**H6**	Social class $^{+}\rightarrow$ Entitlement	.002	.002	.005	.002	.060	.012	.007	.029
**H7**	Social class × System-justification $^{+}\rightarrow$ Entitlement	.002	.002	.004	.003	.006	<.001	.001	.001
**H8**	Social class $^{-}\rightarrow$ Interdependent self-construal	.002	.002	.004	.002	<.001	<.001	.001	.013
**H9**	Social class $^{+}\rightarrow$ Negative reactions to deindividuation	.002	.002	.005	.002	.017	<.001	.028	.036
**H10**	Social class $^{-}\rightarrow$ Individuals in one's inner circle	.009	.010	.020	.013	.023	.002	.018	.004
**H11**	Social class $^{-}\rightarrow$ Social support received	.010	.012	.025	.023	.004	.003	.004	.021
**H12**	Social class × Stability/Chaos $^{-}\rightarrow$ Communal orientation	.002	.002	.005	.002	<.001	.001	.001	.008
**H13**	Social class × Stability/Chaos $^{+}\rightarrow$ Obsession with money	.002	.002	.005	.002	<.001	<.001	.001	.004
**H14**	Education bias	.002	.002	.004	.002	.062	.009	.028	.087
**H15**	Social class $^{+}\rightarrow$ Education bias	.002	.002	.004	.002	.041	.024	.046	.008
**H16**	Education bias = Ethnic/national bias	.002	n/a	n/a	.002	.009	n/a	n/a	.048
**H17**	Social class $^{-}\rightarrow$ Thematic/holistic thinking style	.002	.002	.004	.002	.003	.006	.028	.002
**H18**	Social class $^{-}\rightarrow$ Anticipation of change	.002	.002	.004	.002	<.001	.018	.067	.016
**H19**	Social class $^{-}\rightarrow$ Contextual explanations	.002	.002	.004	.002	.004	<.001	.008	.044
**H20**	Social class $^{+}\rightarrow$ Sense of control $^{-}\rightarrow$ contextual explanation	.002	.002	.005	.002	<.001	<.001	.007	.001
**H21**	Social class $^{-}\rightarrow$ Compassion (other-oriented emotion)	.002	.002	.004	.002	.002	<.001	.001	<.001
**H22**	Social class $^{-}\rightarrow$ Love (other-oriented emotion)	.002	.002	.004	.002	.002	.002	<.001	<.001
**H23**	Social class $^{+}\rightarrow$ Contentment (self-oriented emotion)	.002	.002	.004	.002	.013	.007	.013	.001
**H24**	Social class $^{+}\rightarrow$ Pride (self-oriented emotion)	.002	.002	.004	.002	.001	<.001	<.001	.006
**H25**	Social class × Sharing × Time $^{-}\rightarrow$ Self-conscious emotions	.008	.008	.018	.008	<.001	<.001	.001	<.001
**H25′**	Social class × Sharing × Time × Type $^{-}\rightarrow$ Negat. emotions	.008	.008	.018	.008	.001	.001	<.001	.001
**H26**	Social class $^{-}\rightarrow$ Influence of contextual information	.002	.002	.005	.002	.007	.002	<.001	.009
**H27**	Social class × Self/Other benefits $^{+}\rightarrow$ Unethical behavior	.009	.008	.017	.008	.001	.001	<.001	<.001

Table S9 - Primary Analysis. *Continued*

ID	Hypothesis	MDE with 90% power (in η_p^2)				Partial eta squared (η_p^2)			
		USA	FR	CHE	India	USA	France	CHE	India
**H28**	Social class × Private/Public $^{-}$→ Prosocial behavior	.008	.008	.018	.008	<.001	<.001	<.001	<.001
**H29**	Social class $^{+}$→ Unethical behaviors	.002	.002	.005	.002	.001	.001	.001	.016
**H30**	Social class $^{+}$→ Preference of delayed reward	.009	.009	.019	.008	.001	.002	<.001	.041
**H31**	Social class $^{-}$→ Risk aversion	.009	.009	.019	.008	.020	.021	.003	.008
**H32**	Social class × Mortality/control $^{\emptyset}$→ Delayed reward	.013	.014	.021	.012	.004	.001	<.001	.002
**H33**	Social class × Mortality (vs. control) $^{\emptyset}$→ Risk aversion	.013	.014	.021	.012	<.001	<.001	.001	.001
**H34**	Social class × Hard/Easy problems $^{+}$→ Performance	.013	.012	.019	.012	.003	.009	.004	<.001
**H35**	Social class $^{+}$→ Utilitarian moral decision-making	.005	n/a	n/a	.002	.023	n/a	n/a	.008

Note. This figure presents the values of the minimum detectable effect (MDE) and partial eta squared displayed in the upper and right panels of Figure 1 in the main manuscript. 'n/a' means 'not applicable.' This designation is used when the variable was missing from the questionnaire (for **H16** and **H35**).

Table S10 - Secondary Analysis. Pooled Within-Country Correlation Coefficient Between All Social Class Indicators

	(1)	(2)	(3)	(4)	(5)	(6)	(7)	(8)	(9)
**Education** (linear contrast) (1)	1.00								
**Income** (log-transformed, rescaled) (2)	.29***	1.00							
**Occupation** (autonomy) (3)	.36***	.32***	1.00						
**Subjective SES** (MacArthur scale) (4)	.22***	.26***	.30***	1.00					
**Childhood subjective SES** (5)	.10***	.09***	.10***	.52***	1.00				
**Childhood SES** (Griskevicius) (6)	.14***	.12***	.14***	.33***	.51***	1.00			
**Social class self-categorization** (7)	.33***	.31***	.34***	.53***	.34***	.34***	1.00		
**Low financial scarcity**[R] (8)	.13***	.19***	.19***	.32***	.17***	.17***	.32***	1.00	
**Sense of power** (Dubois) (9)	.12***	.14***	.18***	.32***	.22***	.46***	.28***	.18***	1.00

Note. Social class indicators were demeaned (i.e., the country-specific mean was subtracted from each observation) as in the fixed-effects model used in the secondary and tertiary analyses (see Hamaker & Muthén, 2020); this enabled us to estimate the pooled within-country correlation coefficient. Education is the planned contrast (-1 = "lowest group," 0 = "middle," 1 = "highest"). Income was log-transformed and rescaled within countries to account for differences in monetary units (i.e., 1 unit = 1 $SD(log\text{-}income)_{within}$). Financial scarcity was reverse-coded, with a higher score indicating higher social class.

$^{***}p < .001$

Table S11 - Secondary Analysis. *P*-values for Each Relevant Hypothesis Test in the Pooled Sample Across the Nine Social Class Indicators

		Education	Income	Occupation	Subjective SES	Childhold subj. SES	Childhood SES	Self-categorisation	Financial scarcity[revers.]	Sense of power
**H1**	Sense of control	< .001	< .001	< .001	< .001	< .001	< .001	< .001	< .001	.015
**H2**	Agency	< .001	< .001	< .001	< .001	< .001	< .001	< .001	< .001	< .001
**H3**	Persistence in goal striving	< .001	< .001	< .001	< .001	< .001	< .001	< .001	< .001	< .001
**H4**	Self-esteem	< .001	< .001	< .001	< .001	.704	.441	< .001	< .001	< .001
**H5**	Narcissism	.771	< .001	< .001	< .001	< .001	< .001	< .001	< .001	< .001
**H6**	Entitlement	.454	< .001	.001	< .001	< .001	< .001	< .001	< .001	< .001
**H7**	Entitlement (mod.: System-justification)	.003	.001	< .001	< .001	< .001	< .001	< .001	< .001	< .001
**H8**	Interdependent self-construal	.374	.257	.394	< .001	< .001	< .001	< .001	.001	< .001
**H9**	Neg. reactions to reduced individuation	< .001	< .001	< .001	< .001	.084	.082	< .001	< .001	.002
**H10**	Individuals in one's inner circle	< .001	.003	< .001	< .001	.029	< .001	< .001	< .001	< .001
**H11**	Social support received	< .001	.004	.394	.478	.331	.475	.094	.017	.037
**H12**	Communal orientation (mod.: Stability/Chaos)	< .001	< .001	< .001	.013	.717	.053	< .001	< .001	.002
**H13**	Obsession for money (mod.: Stability/Chaos)	.608	.935	.593	.001	< .001	< .001	.390	.557	.030
**H15**	Education bias	< .001	< .001	< .001	< .001	< .001	< .001	< .001	< .001	< .001
**H17**	Thematic thinking style	< .001	< .001	< .001	< .001	< .001	< .001	< .001	.003	< .001
**H18**	Anticipation of change	< .001	.489	< .001	< .001	< .001	< .001	< .001	< .001	< .001
**H19**	Contextual explanations	.223	.117	.021	< .001	< .001	< .001	< .001	.003	< .001
**H21**	Compassion (other-oriented emotion)	< .001	< .001	< .001	< .001	.012	< .001	.012	.512	< .001
**H22**	Love (other-oriented emotion)	< .001	< .001	< .001	< .001	.013	< .001	< .001	< .001	< .001
**H23**	Contentment (self-oriented emotion)	< .001	< .001	< .001	< .001	< .001	< .001	< .001	< .001	< .001
**H24**	Pride (self-oriented emotion)	< .001	< .001	< .001	< .001	< .001	< .001	< .001	< .001	< .001
**H25**	Self-conscious emotions (mod.: Low/Equal sharing)	.585	.165	.323	.723	.718	.386	.327	.467	.324
**H26**	Influence of contextual information	.014	.014	< .001	< .001	< .001	< .001	< .001	.002	.002
**H27**	Unethical behavior (mod.: Self/Other benefit)	.681	.933	.481	.153	.843	.220	.797	.896	.896
**H28**	Prosocial behavior (mod.: Private/Public context)	.681	.616	.685	.339	.170	.509	.898	.696	.186
**H29**	Unethical behaviors	.013	< .001	.006	< .001	< .001	< .001	< .001	.002	< .001
**H30**	Preference for delayed reward	< .001	< .001	< .001	< .001	< .001	.001	< .001	< .001	.331
**H31**	Risk aversion	.988	.683	< .001	< .001	< .001	< .001	< .001	< .001	< .001
**H34**	Performance (mod.: Easy/Hard financial problems)	.127	.002	.885	.364	.832	.005	.474	.778	.188
**H35**	Utilitarian moral decision making	.015	.009	.601	< .001	< .001	< .001	< .001	< .001	< .001

Table S12 - Tertiary Analysis. *Coefficient Estimates for the Interaction and Simple Slopes for Each Moderator/Indicator in the Pooled Sample*

**Mod: Social Class Identification**	**Education**	**Income**	**Occupation**	**Subj. SES**	**Child subj.**	**Child SES**	**Self-category**	**Scarcity[r]**	**Power**
**H1′** Sense of control (+)	-.04***	-.03***	-.01	-.08***	-.03***	-.06***	-.06***	-.03***	-.08***
-1SD/+1SD	.14***/.07***	.12***/.07***	n/a	.19***/.04***	.02*/-.05***	-.03*/-.14***	.17***/.04***	.14***/09***	.11***/-.05***
**H2′** Agency (+)	.03***	.02***	.03***	.03***	.04***	.09***	.02***	.03***	.05***
-1SD/+1SD	.00/.05***	.06***/.11***	.09***/.15***	.16***/.22***	.07***/.14***	.13***/.32***	.13***/.18***	.05***/.12***	.41***/.51***
**H3′** Persistence (+)	.00	-.04***	.01	-.02*	.00	.07***	-.03***	-.02	.04***
-1SD/+1SD	n/a	.16***/.09***	n/a	.12***/.08***	n/a	.04***/.18***	.10***/.04***	n/a	.26***/.34***
**H4′** Self-esteem (+)	.00	-.01	.00	-.07***	-.03***	-.01	-.05***	-.02**	-.05***
-1SD/+1SD	n/a	n/a	n/a	.22***/.08***	.03*/-.03**	n/a	.18***/.08***	.16***/.12***	.25***/.15***
**H5′** Narcissism (+)	.04***	.04***	.03***	.05***	.04***	.10***	.04***	.03***	.08***
-1SD/+1SD	-.04***/.03***	-.03***/.05***	-.01/.06***	.04***/.14***	.04***/.13***	.17***/.36***	.02/.10***	.02*/.07***	.32***/.48***
**H6′** Entitlement (+)	.06***	.04***	.05***	.06***	.05***	.10***	.06***	.04***	.09***
-1SD/+1SD	-.06***/.06***	-.03***/.05***	-.05***/.05***	.01/.12***	.02*/.12***	.13***/.33***	-.00/.12***	-.02/.05***	.30***/.48***
**H8′** Interdependence[a] (+)	.02**	.02*	.01	.01	.01	.01	.00	.01	.04***
-1SD/+1SD	-.03**/.01	-.04***/.00	n/a	n/a	n/a	n/a	n/a	n/a	-.15***/-.06***
**H9′** Deindividuation[a] (-)	.03	.02	-.01	-.05	-.02	-.04	-.04	-.02	.02
-1SD/+1SD	n/a	n/a	n/a	n/a	n/a	n/a	n/a	n/a	n/a
**H10′** Inner circle (-)	.02	.03	.01	.02	-.02	-.01	.02	.02	.01
-1SD/+1SD	n/a	n/a	n/a	n/a	n/a	n/a	n/a	n/a	n/a
**H11′** Social support (-)	-.01	.00	-.01	-.04	-.03	-.05*	-.04	-.04	-.04
-1SD/+1SD	n/a	n/a	n/a	n/a	n/a	.07/-.04	n/a	n/a	n/a
**H15′** Education bias (+)	.03***	.02	.03**	-.01	-.01	.01	-.01	.00	.01
-1SD/+1SD	.13***/.19***	n/a	.08***/.13***	n/a	n/a	n/a	n/a	n/a	n/a
**H17′** Thematic/holistic (-)	.00	-.01	.01	-.01	-.01	-.02*	.00	.01	-.02
-1SD/+1SD	n/a	n/a	n/a	n/a	n/a	-.04***/-.08***	n/a	n/a	n/a
**H18′** Anticipate change[a] (+)	.01	.04***	.02**	.09***	.09***	.11***	.08***	.08***	.09***
-1SD/+1SD	n/a	-.05***/.03***	-.06***/-.02	.04***/.22***	.07***/.24***	-.02/.20***	-.04***/.11***	-.06***/.09***	-.01/.18***
**H19′** Contextual explanation[a] (+)	.01	.02*	.00	.07***	.06***	.06***	.02*	.01	.06***
-1SD/+1SD	n/a	-.01/.02	n/a	-.01/.13***	.03/.15***	.09***/.22***	-.00/.03**	n/a	.07***/.18***
**H21′** Compassion (other)[a] (+)	.01	-.03***	.01	-.01	-.01	.07***	-.02*	-.00	.07***
-1SD/+1SD	n/a	.10***/.05***	n/a	n/a	n/a	.04***/.18***	.02/-.02	n/a	.20***/.33***
**H22′** Love (other)[a] (+)	.02	-.02**	.01	-.01	-.01	.07***	-.03**	-.01	.07***
-1SD/+1SD	n/a	.11***/.07***	n/a	n/a	n/a	.05***/.19***	.04**/-.01	n/a	.18***/.32***
**H23′** Contentment (self) (+)	.03**	-.00	.01	-.02*	.01	.09***	-.01	-.00	.06***
-1SD/+1SD	.00/.06***	n/a	n/a	.21***/.17***	n/a	.12***/.30***	n/a	n/a	.32***/.45***
**H24′** Pride (self) (+)	.02	-.01	.01	-.02	-.01	.07***	-.02	-.01	.05***
-1SD/+1SD	n/a	n/a	n/a	n/a	n/a	.08***/.22***	n/a	n/a	.30***/.40***
**H26′** Contextual influence (+)	.00	.01	.01	-.00	-.00	.01	-.00	.01	-.00
-1SD/+1SD	n/a	n/a	n/a	n/a	n/a	n/a	n/a	n/a	n/a
**H29′** Unethical behavior[a] (-)	.01	-.01	.01	-.05***	-.05***	-.04***	-.03***	-.02**	-.03***
-1SD/+1SD	n/a	n/a	n/a	-.03*/-.14***	-.03*/-.13***	.02/-.07***	-.00/-.06***	.00/-.04***	.01/-.05***
**H30′** Delayed reward (+)	-.00	.00	-.02	-.01	.01	-.02	-.05**	-.06***	-.05**
-1SD/+1SD	n/a	n/a	n/a	n/a	n/a	n/a	.15***/.05*	.19***/.07**	.06**/-.03
**H31′** Risk aversion (-)	-.03	-.03	-.03	-.03*	-.04*	-.05***	-.05***	-.06***	-.03*
-1SD/+1SD	n/a	n/a	n/a	-.08***/-.15***	-.05/-.13***	-.05/-.16***	-.04/-.15***	-.04/-.16***	-.08***/-.15***
**H35′** Utilitarian decision (+)	.13***	.13***	.10***	.18***	.16***	.17***	.17***	.12***	.12***
-1SD/+1SD	-.18***/.08***	-.18***/.08**	-.14***/.05*	.01/.36***	.11***/.43***	.14***/.48***	.03/.37***	.01/.25***	.15***/.40***

Table S12 - Tertiary Analysis. *Continued.*

Mod: System-Justifying Beliefs		Education	Income	Occupation	Subj. SES	Child subj.	Child SES	Self-category	Scarcity^{r}	Power
**H1′**	Sense of control (+)	-.02*	-.03***	.00	-.13***	-.10***	-.13***	-.09***	-.06***	-.18***
	-1SD/+1SD	.12***/.08***	.12***/.06***	n/a	.22***/-.04***	.07***/-.13***	.04***/-.22***	.18***/.00	.17***/.05***	.20***/-.15***
**H2′**	Agency (+)	.00	.01	.03***	.06***	.06***	.11***	.04***	.04***	.05***
	-1SD/+1SD	n/a	n/a	.08***/.14***	.12***/.24***	.04***/.16***	.07***/.29***	.10***/.18***	.02/.10***	.41***/.50***
**H3′**	Persistence (+)	-.03***	-.02**	.00	.05***	.05***	.05***	.01	.04***	-.02**
	-1SD/+1SD	.10***/.04***	.15***/.10***	n/a	.03**/.12***	-.06***/.04***	.02/.11***	n/a	.00/.08***	.30***/.26***
**H4′**	Self-esteem (+)	-.01	-.03***	-.01	-.08***	-.04***	-.08***	-.06***	-.03***	-.13***
	-1SD/+1SD	n/a	.16***/.11***	n/a	.20***/.05***	.02*/-.06***	.05***/-.10***	.17***/.05***	.15***/.10***	.31***/.05***
**H5′**	Narcissism (+)	-.01	.00	.01	.11***	.11***	.13***	.06***	.05***	.10***
	-1SD/+1SD	n/a	n/a	n/a	-.03***/.18***	-.02*/.19***	.08***/.34***	-.03**/.10***	-.04***/.05***	.26***/.46***
**H6′**	Entitlement (+)	.02**	.02**	.03***	.12***	.10***	.14***	.10***	.07***	.10***
	-1SD/+1SD	-.01/.03**	-.02/.02**	-.04***/.03*	-.06***/.19***	-.03**/.17***	.05***/.33***	-.06***/.14***	-.08***/.06***	.27***/.48***
**H8′**	Interdependence^{a} (+)	.00	.02	.00	.04***	.03***	.05***	.02*	.02	.09***
	-1SD/+1SD	n/a	n/a	n/a	-.03*/.05***	.01/.07***	.00/.10***	.00/.04***	n/a	-.21***/-.03*
**H9′**	Deindividuation^{a} (-)	-.04	-.04	-.04	-.01	.04	.08*	-.01	-.02	.13***
	-1SD/+1SD	n/a	n/a	n/a	n/a	n/a	.00/.01***	n/a	n/a	-.01***/.01**
**H10′**	Inner circle (-)	.02	.01	-.00	.01	-.02	-.02	-.00	.01	-.02
	-1SD/+1SD	n/a	n/a	n/a	n/a	n/a	n/a	n/a	n/a	n/a
**H11′**	Social support (-)	-.03	-.02	-.01	-.05*	-.06**	-.08***	-.05*	-.03	-.08***
	-1SD/+1SD	n/a	n/a	n/a	.08**/-.01	.05/-.06	.09***/-.07*	.09**/-.00	n/a	.07*/-.10***
**H15′**	Education bias (+)	-.02**	-.00	.01	-.03***	-.02**	-.04***	-.03***	-.00	-.02**
	-1SD/+1SD	.18***/.14***	n/a	n/a	.04***/.00	.04***/-.01	.09***/.01	.13***/.06***	n/a	.07***/.03**
**H17′**	Thematic/holistic (-)	-.00	-.00	-.00	-.02	-.01	-.02*	-.00	.01	-.03***
	-1SD/+1SD	n/a	n/a	n/a	n/a	n/a	-.04**/-.08***	n/a	n/a	-.02/-.07***
**H18′**	Anticipate change^{a} (+)	.07***	.06***	.03***	.11***	.10***	.15***	.09***	.05***	.14***
	-1SD/+1SD	-.16***/-.03**	-.07***/.05***	-.07***/-.01	.04***/.25***	.07***/.27***	-.07***/.23***	-.05***/.13***	-.04***/.05***	-.06***/.21***
**H19′**	Contextual explanation^{a} (+)	-.01	.02**	-.02	.09***	.10***	.13***	.07***	-.00	.13***
	-1SD/+1SD	n/a	-.01/.03**	n/a	-.01/.17***	.01/.20***	.04***/.30***	-.05***/.09***	n/a	.02/.28***
**H21′**	Compassion (other)^{a} (+)	-.04***	-.01	-.00	.09***	.08***	.07***	.04***	.05***	.03***
	-1SD/+1SD	.08***/-.00	n/a	n/a	-.08***/.10***	-.10***/.06***	.02/.16***	-.06***/.03*	-.08***/.02	.23***/.29***
**H22′**	Love (other)^{a} (+)	-.03***	-.02*	.00	.10***	.09***	.07***	.05***	.07***	.03***
	-1SD/+1SD	.09***/.02	.10***/.07***	n/a	-.10***/.10***	-.11***/.07***	.02/.16***	-.06***/.04***	-.07***/.06***	.21***/.27***
**H23′**	Contentment (self) (+)	-.03***	-.02**	-.02**	.02**	.04***	.05***	.00	.01	.00
	-1SD/+1SD	.07***/.00	.11***/.07***	.11***/.07***	.11***/.15***	-.01/.07***	.06***/.16***	n/a	n/a	n/a
**H24′**	Pride (self) (+)	-.04***	-.02*	-.01	.08***	.08***	.06***	.03***	.05***	.01
	-1SD/+1SD	.11***/.04***	.11***/.07***	n/a	-.03*/.13***	-.07***/.08***	.05***/.17***	.00/.06***	-.03**/.08***	n/a
**H26′**	Contextual influence (+)	.03**	.01	-.02*	-.02**	-.00	.01	-.01	-.02*	.02
	-1SD/+1SD	-.04***/.01	n/a	.05***/.01	.09***/.05***	n/a	n/a	n/a	.03**/-.01	n/a
**H29′**	Unethical behavior^{a} (-)	-.02	-.02*	.01	-.04***	-.04***	-.05***	-.03***	-.03***	-.04***
	-1SD/+1SD	n/a	-.02/-.05***	n/a	-.04***/-.13***	-.04***/-.13***	.03*/-.07***	.00/-.07***	.01/-.04***	.03*/-.06***
**H30′**	Delayed reward (+)	-.04*	.01	-.04*	-.03	-.01	-.05**	-.07***	-.06***	-.04*
	-1SD/+1SD	.16***/.08***	n/a	.13***/.05	n/a	n/a	.09***/-.00	.16***/.02	.18***/.06*	.03/-.04
**H31′**	Risk aversion (-)	.02	.02	.01	-.00	-.02	-.07***	-.03	-.01	-.07***
	-1SD/+1SD	n/a	n/a	n/a	n/a	n/a	-.00/-.14***	n/a	n/a	-.01/-.14***
**H35′**	Utilitarian decision (+)	.10***	.10***	.04*	.08***	.12***	.22***	.13***	.04*	.22***
	-1SD/+1SD	-.14***/.06**	-.17***/.03	-.10***/-.01	.11***/.28***	.16***/.41***	.01/.44***	.06/.32***	.04/.12***	-.04/.39***

Table S12 - Tertiary Analysis. *Continued.*

Mod: Local Income Inequality		Education	Income	Occupation	Subj. SES	Child subj.	Child SES	Self-category	Scarcity^{r}	Power
**H1′**	Sense of control (+)	-.01	-.03*	-.01	-.04***	-.02	-.03**	-.02*	-.02*	-.05***
	-1SD/+1SD	n/a	.12***/.07***	n/a	.12***/.05***	n/a	-.07***/-.13***	.11***/.06***	.13***/.08***	.06***/-.05***
**H2′**	Agency (+)	.01	.00	.01	.01	.03	.04***	.02	.02	.01
	-1SD/+1SD	n/a	n/a	n/a	n/a	n/a	.23***/.31***	n/a	n/a	n/a
**H3′**	Persistence (+)	.03*	.01	.01	-.01	.00	.03**	.03*	-.01	.02
	-1SD/+1SD	.05***/.10***	n/a	n/a	n/a	n/a	.10***/.17***	.05***/.11***	n/a	n/a
**H4′**	Self-esteem (+)	.01	-.03*	.01	-.04***	-.01	.00	-.02	-.02	-.02
	-1SD/+1SD	n/a	.18***/.12***	n/a	.19***/.11***	n/a	n/a	n/a	n/a	n/a
**H5′**	Narcissism (+)	.01	.01	.02	.01	.00	.05***	.00	.01	.05***
	-1SD/+1SD	n/a	n/a	n/a	n/a	n/a	.27***/.36***	n/a	n/a	.40***/.49***
**H6′**	Entitlement (+)	.01	.02	.03***	.02	.01	.06***	.03**	.02*	.06***
	-1SD/+1SD	n/a	n/a	-.01/.06***	n/a	n/a	.23***/.35***	.07***/.13***	.02/.07***	.37***/.50***
**H8′**	Interdependence^{a} (+)	.03*	.01	.02	.05***	.04**	.02	.03*	.03*	.04***
	-1SD/+1SD	-.04**/.02	n/a	n/a	-.03/.07***	.01/.08***	n/a	.01/.06***	-.01/.05***	-.11***/-.03
**H9′**	Deindividuation^{a} (-)	.02	.04	-.02	-.09	-.15***	-.08	-.14**	-.05	-.04
	-1SD/+1SD	n/a	n/a	n/a	n/a	.01**/-.01**	n/a	-.00/-.02***	n/a	n/a
**H10′**	Inner circle (-)	-.02	-.01	-.02	-.03	-.03	-.03	-.06	-.06*	-.02
	-1SD/+1SD	n/a	n/a	n/a	n/a	n/a	n/a	n/a	-.01/-.13***	n/a
**H11′**	Social support (-)	.01	-.01	.01	-.01	.02	.02	.02	-.03	-.03
	-1SD/+1SD	n/a	n/a	n/a	n/a	n/a	n/a	n/a	n/a	n/a
**H15′**	Education bias (+)	.03*	.01	.02	.03	.00	.02	.01	-.01	.01
	-1SD/+1SD	.13***/.19***	n/a	n/a	n/a	n/a	n/a	n/a	n/a	n/a
**H17′**	Thematic/holistic (-)	.02	.02	-.00	.01	-.01	-.01	.02	-.00	-.00
	-1SD/+1SD	n/a	n/a	n/a	n/a	n/a	n/a	n/a	n/a	n/a
**H18′**	Anticipate change^{a} (+)	-.01	.01	.02	.06***	.05***	.04***	.04***	.04***	.04***
	-1SD/+1SD	n/a	n/a	n/a	.10***/.23***	.14***/.24***	.08***/.17***	.02/.10***	-.01/.07***	.08***/.16***
**H19′**	Contextual explanation^{a} (+)	-.03*	.01	.04***	.05***	.02	.04***	-.00	.03**	.04***
	-1SD/+1SD	.01/-.04**	n/a	-.02/.06***	.04**/.14***	n/a	.14***/.22***	n/a	-.02/.05***	.11***/.19***
**H21′**	Compassion (other)^{a} (+)	.02*	.01	.02	-.02	-.02	.03**	.01	-.00	.04**
	-1SD/+1SD	.02/.07***	n/a	n/a	n/a	n/a	.11***/.18***	n/a	n/a	.25***/.32***
**H22′**	Love (other)^{a} (+)	.04***	.01	.03	-.01	-.02	.05***	.02	.00	.06***
	-1SD/+1SD	.01/.10***	n/a	n/a	n/a	n/a	.10***/.20***	n/a	n/a	.21***/.34***
**H23′**	Contentment (self) (+)	.02	.01	.03**	-.02	-.01	.05***	.02	.01	.06***
	-1SD/+1SD	n/a	n/a	.09***/.16***	n/a	n/a	.19***/.30***	n/a	n/a	.35***/.46***
**H24′**	Pride (self) (+)	.04***	.01	.02	-.01	-.01	.05***	.01	-.01	.04***
	-1SD/+1SD	.04***/.12***	n/a	n/a	n/a	n/a	.14***/.24***	n/a	n/a	.33***/.41***
**H26′**	Contextual influence (+)	-.02	.01	.01	-.01	-.00	-.03**	-.01	-.00	-.01
	-1SD/+1SD	n/a	n/a	n/a	n/a	n/a	.06***/-.00	n/a	n/a	n/a
**H29′**	Unethical behavior^{a} (-)	.01	-.01	-.00	-.04***	-.05***	-.02	-.03*	-.03**	-.01
	-1SD/+1SD	n/a	n/a	n/a	-.04**/-.13***	-.03*/-.13***	n/a	-.01/-.06***	.01/-.05***	n/a
**H30′**	Delayed reward (+)	-.04	-.00	-.03	-.00	.00	-.02	-.01	-.01	-.02
	-1SD/+1SD	n/a	n/a	n/a	n/a	n/a	n/a	n/a	n/a	n/a
**H31′**	Risk aversion (-)	-.01	.00	-.00	-.02	-.01	-.01	-.03	-.03	-.02
	-1SD/+1SD	n/a	n/a	n/a	n/a	n/a	n/a	n/a	n/a	n/a
**H35′**	Utilitarian decision (+)	.03	.08***	.01	.18***	.09***	.09***	.20***	.05	.08**
	-1SD/+1SD	n/a	-.14***/.03	n/a	.09**/.45***	.26***/.43***	.30***/.47***	.04/.45***	n/a	.28***/.44***

Note. The sign of the base statistical effect of social class is provided in parentheses next to each outcome. Coefficients highlighted in blue indicate *strengthening* interactions (i.e., where the strength of the hypothesized effect of social class, whether positive or negative, intensifies as the moderator increases), whereas coefficients highlighted in orange indicate *weakening* interactions (i.e., where the strength of the hypothesized effect of social class, whether positive or negative, attenuates as the moderator increases). The lines "-1SD/+1SD" indicate the simple slopes for the social class indicator at -1 standard deviation and +1 standard deviation of the moderator, respectively. All predictors and outcomes were standardized, which means that these simple slopes can roughly be interpreted as correlations, with two exceptions: (i) when social class is operationalized using education – in that case, the coefficient captures the change from the lowest to the highest education group; (iii) when the outcome is binary – in this case, logistic rather than linear regression was used, and log-odds are reported (i.e., for **H9′** and **H35′**). Education is the planned contrast (-1 = "lowest group," 0 = "middle," 1 = "highest"). Income was log-transformed and rescaled within countries to account for differences in monetary units (i.e., 1 unit = 1 $SD(log\text{-}income)_{within}$). Financial scarcity was reverse-coded, with a higher score indicating higher social class. To conserve space, the zero-unit digits of the *B*s are omitted.

[a] For these outcomes, the statistical effect of social class observed in the secondary analysis was in the opposite direction to the original hypothesis for the majority of the social class indicators, and the reinforcing and weakening interactions therefore pertain to the observed rather than the hypothesized effects.

$^{***}p < .001$, $^{**}p < .01$, $^{**}p < .05$ (significance levels adjusted for multiple testing for each hypothesis using sequential Bonferroni procedure)

List of Deviations From the Original Registered Report and Preregistration

Theoretical Material

(1) In the final manuscript, we discuss the number of *hypotheses* tested (35) rather than the number of *effects* tested (43). Some hypotheses correspond to multiple effects in the original studies and questionnaire (e.g., hypothesis **H9**, which examines the link between social class and negative reactions to reduced individuation, is tested using three outcomes). However, we chose to focus on the number of hypotheses rather than the number of effects to avoid redundancy and enhance clarity.

(2) In the final manuscript, we use a new numbering system for the hypotheses tested in the tertiary analysis, which consists of modifying the label of the corresponding hypothesis in the primary analysis by adding a prime symbol (e.g., **H1** becomes **H1′**)

(3) In the final manuscript, we refer to the link between social class and time preference as "preference for delayed reward" rather than "delayed gratification." This terminology was deemed more conceptually accurate.

(4) In the final manuscript, we corrected a mistake in **Table 1** by replacing "preference for risky option" with "risk aversion" to accurately describe the expected link between social class and risk taking.

(5) In the final manuscript, we changed the examples for other-oriented emotions by including the emotions measured in the study (i.e., compassion and love instead of compassion and guilt) to enhance coherence and clarity.

Questionnaire

(1) For the French and Swiss questionnaire, due to a shortcoming during the translation process, we failed to adapt the ethnic/national outgroups used in the thermometer ratings, meaning that ethnic/national biases could not be computed (see *Main Manuscript, Methods, Design, Questionnaire, Outcome Variables: Self-Reported Scales or Short Tasks, Relationships*).

(2) For the French and Swiss questionnaires, due to a shortcoming during the construction process, we failed to include the perceived availability of resources scale, meaning that H35 could not be tested in the primary analysis (see *Main Manuscript, Design, Questionnaire, Outcome Variables: Self-Reported Scales or Short Tasks, Decision-making*) .

Sampling

(1) For all samples, we applied two non-preregistered exclusion criteria. First, we removed duplicates based on IP addresses and demographics. Second, we observed problems with the distribution of response times and therefore excluded participants who completed the survey in less than nine minutes—the minimum duration deemed necessary to complete the survey (see *Main Manuscript*, *Sampling Plan*, *Exclusion Criteria*).

(2) For the U.S. and French samples, we retained participants who had passed at least one attention check, rather than retaining participants who had passed both attention checks as originally preregistered (see *Main Manuscript*, *Design*, *Attention Checks*).

(3) For the Swiss sample, we achieved a response rate of 12%, instead of the 20% originally

expected in the registered report, meaning that the *N* was only 75% of the minimum target (see *Main Manuscript*, *Sampling Plan*, *Data Collection*, *Swiss Sample*).

Analysis

(1) For all relevant analyses, unemployed participants were assigned a score of zero on the occupation scale, rather than excluded from the analysis as preregistered. We did this to avoid excluding a whole category of person and before performing any inferential tests (see *Main Manuscript*, *Design*, *Predictor Variables*, *Occupation*).

(2) For income, when responses to the open-ended question about annual income fell outside the brackets selected in the previous closed-ended question (i.e., presenting deciles), we used the midpoint of the income brackets. These responses seemed too ambiguous to be deemed reliable. We made this decision before performing any inferential tests (see *Main Manuscript*, *Design*, *Predictor Variables*, *Income*).

(3) For all relevant analyses, income was log-transformed, even though no transformations were anticipated in the pre-registration. The reason was that income was highly skewed and robust estimation of SEs was insufficient to address the problem (see *Main Manuscript*, *Methods*, *Analysis Plan*, *Data Transformation*).

(4) For all relevant analyses, education was contrast-coded. As preregistered, the planned contrast compared the lowest educational group to the highest educational group, but we used the codes -0.5 and +0.5 instead of -1 and +1 to ease the interpretation of the estimates (i.e., they represent the difference between the bottom and top groups; see *Main Manuscript*, *Design*, *Predictor Variables, Education*).

(5) For the secondary analysis, that focuses on predictive strength of social class, we decided to not repeat analyses testing hypotheses that did not predict a social class effect (**H14**, **H16**), predicted null moderations (**H32**, **H33**) or indirect effects (**H20**; see *Main Manuscript*, *Analysis Plan, Confirmatory Analysis and Expected Outcomes for the Secondary Analysis (Indicators))*.

(6) For the secondary and tertiary analyses, income was rescaled within countries to account for differences in monetary units, rather than broken into quintiles as preregistered. The reason was that rescaling provided much more precise estimates, while using quintiles created discrepancies between the primary analysis (using log-income) and the others (see *Main Manuscript*, *Analysis Plan, Confirmatory Analysis and Expected Outcomes for the Secondary Analysis (Indicators))*.

(7) For the tertiary analysis, we applied the same sequential Bonferroni procedure correction for multiple tests used in the secondary analysis for tests pertaining to a similar outcome but using different measures of social class, rather than applying this procedure only in the secondary analysis as described in the original registered report (see *Main Manuscript*, *Analysis Plan, Correction for Multiple Tests*).

(8) For the tertiary analysis, we assessed the moderator as strengthening/weakening the *observed* effect of social class when the majority of indicators showed an effect contrary to the original hypothesis in the secondary analysis, rather than consistently focusing on the originally predicted effect (see *Main Manuscript*, **Figure 3**, *Notes*, *Superscript a*).

(9) For **H27**, "self-beneficial" was coded as +0.5 and "other-beneficial" was coded as -0.5 contrary to the preregistration which had the coding the other way around (see *Main*

Manuscript, Design, Questionnaire, Outcome Variables: Experiments or Longer Task, Social Behavior: Self - (vs. Other-) Benefits and Unethical Behaviors (H27)).

(10) For **E4** and **E5**, we excluded participants identified as rushers from the analysis involving the manipulated variable, rather than including them as preregistered. This decision prioritized response quality over blindly maximizing sample size (see *Main Manuscript*, **Table 2**, *Note*, *Superscript D*).

References

AFC (2020). Statistiques sur l'impôt fédéral direct : Chiffres-clés statistiques des personnes physiques [Direct federal tax statistics: Key figures for personal income tax statistics]. https://www.estv.admin.ch/estv/fr/accueil/afc/statistiques-fiscales/statistiques-fiscales-general/statistiques-impot-federal-direct.html#1356731360

Borenstein, M., & Hedges, L. V. (2019). Effect sizes for meta-analysis. In H. Cooper, L. V. Hedges, & J. Valentine (Eds.), *The handbook of research synthesis and meta-analysis* (3rd ed.) (pp. 207-244). Rusell Sage Foundation.

FSO (2022a). Federal Statistical Office - Look for statistics - Population. https://www.bfs.admin.ch/bfs/en/home/statistics/population.assetdetail.23145958.html

FSO (2022b). Educational attainment by sex and age group https://www.bfs.admin.ch/bfs/en/home/statistics/economic-social-situation-population/gender-equality/education/educational-attainment.assetdetail.22024474.html

FSO (2023). Permanent resident population by age, sex and category of citizenship, 2010-2022. https://www.bfs.admin.ch/bfs/en/home/statistics/population/effectif-change/age.assetdetail.26565162.html

Hamaker, E. L., & Muthén, B. (2020). The fixed versus random effects debate and how it relates to centering in multilevel modeling. *Psychological Methods*, *25*(3), 365–379. https://doi.org/10.1037/met0000239

Hoffmeyer-Zlotnik, J. H. P. & Warner, U. (2011). *Measuring occupation and labour status in cross-national comparative surveys*. GESIS.

INSEE. (2020a). Population by sex and age on 1st January 2020, France: Demographic balance sheet 2019. https://www.insee.fr/en/statistiques/2382597?sommaire=2382613

INSEE. (2020b). Structure et distribution des revenus, inégalité des niveaux de vie en 2020 Dispositif Fichier localisé social et fiscal [Income structure and distribution, inequality of living standards in 2020]. https://www.insee.fr/fr/statistiques/6692220

INSEE. (2021). Revenu disponible des ménages [Disposable income of households]. In *Revenus et patrimoine des ménages – Insee Références – Édition 2021*. INSEE. https://www.insee.fr/fr/statistiques/5371205?sommaire=5371304

INSEE. (2022). Niveau d'éducation de la population [2.3 Education level of the population]. In F*rance, portrait social – Insee Références – Édition 2022*. INSEE. https://www.insee.fr/fr/statistiques/6535231?sommaire=6535307

INSEE. (2023). Population par sexe et groupe d'âges: Données annuelles 2023 [Population by sex and age group: Annual data 2023]. https://www.insee.fr/fr/statistiques/2381476

Mohanty, S. K., Govil, D., Chauhan, R. K., Kim, R., & Subramanian, S. V. (2016). Estimates of poverty and inequality in the districts of India, 2011–2012. *Journal of Development Policy and Practice*, *1*(2), 142-202. https://doi.org/10.1177/2455133316642338

Raven, J., Raven, J. C. & Court, J. H. (2000). *Manual for Raven's Progressive Matrices and Vocabulary Scales*. Harcourt Assessment.

SHP Group (2023), Living in Switzerland Waves 1-23 + Covid 19 data (6.0.0) [Dataset]. FORS data service. https://www.swissubase.ch/en/catalogue/studies/6097/19347/datasets/932/2778/overview

Sommet, N., Weissman, D. L., Cheutin, N., & Elliot, A. J. (2023). How many participants do I need to test an interaction? Conducting an appropriate power analysis and achieving sufficient power to detect an interaction. *Advances in Methods and Practices in Psychological Science*, 6(3). https://doi.org/10.1177/25152459231178728

U.S. Census Bureau (2022a). S0101 | Age and Sex: American Community Survey 1-Year Estimates. https://data.census.gov/table/ACSST5Y2020.S0101?q=sex&t=Age+and+Sex&y=2022

U.S. Census Bureau (2022b). S1501 | Educational Attainment: American Community Survey 1-Year Estimates. https://data.census.gov/table?q=educational+attainment&y=2022

U.S. Census Bureau. (2022c). Income in the United States: 2021. In *Income in the United States: 2021. Current Population Reports*. U.S. Government Publishing Office. https://www.census.gov/content/dam/Census/library/publications/2022/demo/p60-276.pdf

U.S. Census Bureau. (2022d). B19083 | Gini Index of Income Inequality: : American Community Survey 5-Year Estimates. https://data.census.gov/table/ACSDT5Y2020.B19083.

UN (2022a). Data portal population division: Sex ratio of the total population - India. https://population.un.org/dataportal/data/indicators/72/locations/356/start/1990/end/2022/table/pivotbylocation

UN (2022b). DemographicYearbook (73rd Issue) [see "7. Population by age, sex and urban/rural residence: latest available year, 2013 - 2022"]. United Nations. https://unstats.un.org/unsd/demographic-social/products/dyb/dybsets/2022.pdf

Verschuere, B., Lin, C. C., Huismann, S., Kleinberg, B., Willemse, M., Mei, E. C. J., ... & Meijer, E. (2023). The use-the-best heuristic facilitates deception detection. *Nature Human Behaviour*, *7*(5), 718-728. https://doi.org/10.1038/s41562-023-01556-2

World Bank (2022a). Educational attainment, at least completed post-secondary, population 25+, total (%) (cumulative) - India https://data.worldbank.org/indicator/SE.SEC.CUAT.PO.ZS?locations=IN

World Bank (2022b). Educational attainment, at least completed post-secondary, population 25+, total (%) (cumulative) - India https://data.worldbank.org/indicator/SE.SEC.CUAT.PO.ZS?locations=IN
