# Supplementary material for: Advancing the psychology of social class with large-scale replications in four countries
Source: Nat Hum Behav. 2025 Jul 15;9(11):2382–403. doi: 10.1038/s41562-025-02234-1 (PMC12634430; doi:10.1038/s41562-025-02234-1)
Supplement: Supplementary file 2 — Reporting Summary [file 41562_2025_2234_MOESM2_ESM.pdf]

nature portfolio

Corresponding author(s): Anatolia Batruch, Nicolas Sommet, Frédérique Autin

Last updated by author(s): Mar 27, 2025

Reporting Summary

Nature Portfolio wishes to improve the reproducibility of the work that we publish. This form provides structure for consistency and transparency in reporting. For further information on Nature Portfolio policies, see our Editorial Policies and the Editorial Policy Checklist.

Please do not complete any field with "not applicable" or n/a. Refer to the help text for what text to use if an item is not relevant to your study.
For final submission: please carefully check your responses for accuracy; you will not be able to make changes later.

Statistics

For all statistical analyses, confirm that the following items are present in the figure legend, table legend, main text, or Methods section.

n/a	Confirmed	
☐	☒	The exact sample size (*n*) for each experimental group/condition, given as a discrete number and unit of measurement
☐	☒	A statement on whether measurements were taken from distinct samples or whether the same sample was measured repeatedly
☐	☒	The statistical test(s) used AND whether they are one- or two-sided<br>*Only common tests should be described solely by name; describe more complex techniques in the Methods section.*
☐	☒	A description of all covariates tested
☐	☒	A description of any assumptions or corrections, such as tests of normality and adjustment for multiple comparisons
☐	☒	A full description of the statistical parameters including central tendency (e.g. means) or other basic estimates (e.g. regression coefficient) AND variation (e.g. standard deviation) or associated estimates of uncertainty (e.g. confidence intervals)
☐	☒	For null hypothesis testing, the test statistic (e.g. *F*, *t*, *r*) with confidence intervals, effect sizes, degrees of freedom and *P* value noted<br>*Give P values as exact values whenever suitable.*
☒	☐	For Bayesian analysis, information on the choice of priors and Markov chain Monte Carlo settings
☐	☒	For hierarchical and complex designs, identification of the appropriate level for tests and full reporting of outcomes
☐	☒	Estimates of effect sizes (e.g. Cohen's *d*, Pearson's *r*), indicating how they were calculated

Our web collection on statistics for biologists contains articles on many of the points above.

Software and code

Policy information about availability of computer code

Data collection	Data collection was conducted through the Qualtrics platform (January 2024). The Qualtrics questionnaires (in all languages) and the Qualtrics raw data are available on the OSF page of the project.
Data analysis	Data were analyzed using either Stata version 18.5 or R version 4.4.1. All Stata and R scripts to reproduce the findings are available on the OSF page of the project. https://osf.io/3tjzs/

For manuscripts utilizing custom algorithms or software that are central to the research but not yet described in published literature, software must be made available to editors and reviewers. We strongly encourage code deposition in a community repository (e.g. GitHub). See the Nature Portfolio guidelines for submitting code & software for further information.

Data

Policy information about availability of data

All manuscripts must include a data availability statement. This statement should provide the following information, where applicable:
- Accession codes, unique identifiers, or web links for publicly available datasets
- A description of any restrictions on data availability
- For clinical datasets or third party data, please ensure that the statement adheres to our policy

The data is available on the OSF page of the project (https://osf.io/3tjzs/). The pooled dataset and a comprehensive codebook can be found in “/Pooled Dataset

(With Codebook)," whereas the four country-specific datasets, along with the full materials (including all questionnaires in the local languages), are available in "/ Individual Datasets (with Materials)."

Research involving human participants, their data, or biological material

Policy information about studies with human participants or human data. See also policy information about sex, gender (identity/presentation), and sexual orientation and race, ethnicity and racism.

Reporting on sex and gender

Gender was collected through self-reported measures, which included an "Other" category with a "Specify" textbox. The gender variable is available in all datasets across the four countries. As shown in Table S3 (Sample Characteristics), the percentage of women in the U.S., French, and Indian samples—each quota-based with gender as one of the quotas—was 53.9%, 51.6%, and 52.1%, respectively. In the Swiss sample—a random probability sample—the percentage of women was 52.5%. The gender variable was included as a covariate in the primary analysis when it was part of the set of control variables specified by the original authors.

Reporting on race, ethnicity, or other socially relevant groupings

Ethnicity/race was measured using the U.S. Census Bureau's categories (e.g., White, African American), with Latinx added as an option. In the French and Swiss samples, ethnicity was replaced with a question about nationality to differentiate citizens from non-citizen residents. In the Indian sample, participants answered a question about religious affiliation (e.g., Hinduism, Islam). In all countries, participants self-identified with the relevant category(ies). These variables were included as covariates in the primary analysis only when they were part of the set of control variables specified by the original authors.

Population characteristics

Sample characteristics for gender, age, income, and education, along with comparisons to national population characteristics, are presented in Table S3 in Supplementary Information.

Recruitment

Our total sample comprised four samples: (i) three quota-based samples (U.S., French, and Indian) recruited by Qualtrics (a market research company) and (ii) one random representative sample (Swiss) recruited by our team. The three quota-based samples were matched to the underlying population in terms of key demographic features. The main advantage of quota sampling is that it ensures each stratum of the population is equally represented in the sample, but its main limitation is that the sample is non-probability-based (i.e., participants self-select into the panel and are hence not representative of the underlying population).

In Switzerland, we used random sampling: (1) the Swiss Federal Statistical Office drew a random sample (stratified by canton) of ≈ 50,000 Swiss addresses; (2) a public institution (the DAL) printed, folded, and assembled 50,000 letters of invitation to participate in our study; and (3) the University of Lausanne sent these 50,000 letters. The main advantage of random sampling is that each individual in the population has the same probability of being invited to participate in the study, but its main limitation is that individuals from specific subgroups (e.g., people with lower income) may have a lower response rate (nonresponse bias). In the context of our research, we believe that the advantage of random sampling compensates for the limitation of quota sampling and vice versa.

Participants recruited by panel providers (U.S., French, and Indian samples) received various types of compensation (e.g., gift cards), whereas participants directly recruited by a local team (Swiss sample) participated in a lottery to receive gift cards.

Ethics oversight

The project was approved by the Research Ethics Board of the University of Lausanne (C_SSP_032020_00004).

Note that full information on the approval of the study protocol must also be provided in the manuscript.

Field-specific reporting

Please select the one below that is the best fit for your research. If you are not sure, read the appropriate sections before making your selection.

☐ Life sciences ☒ Behavioural & social sciences ☐ Ecological, evolutionary & environmental sciences

For a reference copy of the document with all sections, see nature.com/documents/nr-reporting-summary-flat.pdf

Behavioural & social sciences study design

All studies must disclose on these points even when the disclosure is negative.

Study description

The data is quantitative. In an online questionnaire we replicated 17 correlational and five experimental studies in four countries.

Research sample

Our samples included participants from the U.S., France, India, and Switzerland, chosen for both practical and cultural reasons. Among other factors, the U.S. facilitated replication with the same population used in most original studies; France provided a cultural contrast to the U.S., with a long tradition of class consciousness; Switzerland allowed for low-cost random sampling; and India was the only LMIC where we could recruit a large quota-based sample. As indicated in the 'Population Characteristics' and 'Recruitment' text boxes, the samples were representative of the national populations, and relevant demographic information can be found in Table S3 in Supplementary Information.

Sampling strategy

As indicated in the 'Recruitment' textbox, to achieve a sample distribution that matches the underlying populations in the U.S. and France, we used quota sampling with five quotas: (1) income, (2) education, (3) gender, (4) age, and (5) region. In India, for feasibility reasons, we could only use three quotas: income, gender, and age. To achieve national representativeness in Switzerland, we used random sampling. We aimed to reach a power of .95+ for each individual hypothesis. For each country, 9,000 × 2/3 = 6,000 participants per self-reported scale or short task was estimated to yield a power of .99999999712 to detect one small individual effect (f2 = .01). For each country, 9,000 × 1/6 = 1,500 participants per experiment or long task was estimated to yield a power of .97311815015 to detect one small effect (f2 = .01).

Data collection	Data collection was conducted through the Qualtrics platform. Participants completed the study online, either on a computer or a mobile device without the researcher present. They were not informed about the study's hypothesis, and in the case of the experimental condition, they were unaware of their assigned condition until the end of the experiment and debriefing. Because the random allocation was performed by the Qualtrics platform, the researchers were blind to participants' random attribution to experimental conditions.
Timing	In France, data collection started April 28th 2022 and ended August 13 2023. In Switzerland, data collection started May 19th 2022 and ended March 23 2023. In the U.S., data collection started May 21st 2022 and ended October 14 2023. In India, data collection started on February 1st 2023, and ended on December 26st 2023.
Data exclusions	For the U.S., France, and India, Qualtrics removed and replaced participants who provided low-quality responses, as specified in our contract. Following the pre-registered exclusion criteria, data were excluded in the following cases: participants who failed one or two attention checks, those who completed the questionnaire in less than half the median time, and those who consistently provided the same responses on Likert scales. Additionally, we applied two non-preregistered criteria. First, we identified irregularities in the distribution of response times and requested that Qualtrics exclude participants who completed the survey in less than nine minutes—the minimum duration deemed necessary for valid responses. Second, during the data cleaning phase, we identified and excluded 91 duplicate entries based on IP addresses and demographic information. For the Swiss sample, we applied the same criteria as in the other countries. The number of participants excluded is reported in Table S2: Missing data for focal variables (1,225); Completed survey in less than 9 minutes (5); Failed both attention checks (664); Duplicates entries based on IPv4 and demographics (20).
Non-participation	For the U.S., France, and India, Qualtrics does not disclose the number of participants who were reached or those who declined participation, so this information is unavailable. For Switzerland, the participation rate was 12%. No information was available about the characteristics of non-respondents or the reasons for declining participation.
Randomization	The full online questionnaire in Qualtrics contained 17 correlational and five experimental studies. However, through Qualtrics randomization, participants were randomly assigned to complete 2/3 of the short tasks or scales (among the 17 correlation studies). The order of the scales were presented randomly. To avoid cross-contamination, participants were randomly allocated to only one of the five experiments. When completing the experiment, participants were also randomly assigned to either the control or experimental condition.

Reporting for specific materials, systems and methods

We require information from authors about some types of materials, experimental systems and methods used in many studies. Here, indicate whether each material, system or method listed is relevant to your study. If you are not sure if a list item applies to your research, read the appropriate section before selecting a response.

Materials & experimental systems

n/a	Involved in the study
☒	☐ Antibodies
☒	☐ Eukaryotic cell lines
☒	☐ Palaeontology and archaeology
☒	☐ Animals and other organisms
☒	☐ Clinical data
☒	☐ Dual use research of concern
☒	☐ Plants

Methods

n/a	Involved in the study
☒	☐ ChIP-seq
☒	☐ Flow cytometry
☒	☐ MRI-based neuroimaging

Plants

Seed stocks	*Report on the source of all seed stocks or other plant material used. If applicable, state the seed stock centre and catalogue number. If plant specimens were collected from the field, describe the collection location, date and sampling procedures.*
Novel plant genotypes	*Describe the methods by which all novel plant genotypes were produced. This includes those generated by transgenic approaches, gene editing, chemical/radiation-based mutagenesis and hybridization. For transgenic lines, describe the transformation method, the number of independent lines analyzed and the generation upon which experiments were performed. For gene-edited lines, describe the editor used, the endogenous sequence targeted for editing, the targeting guide RNA sequence (if applicable) and how the editor was applied.*
Authentication	*Describe any authentication procedures for each seed stock used or novel genotype generated. Describe any experiments used to assess the effect of a mutation and, where applicable, how potential secondary effects (e.g. second site T-DNA insertions, mosiacism, off-target gene editing) were examined.*

This checklist template is licensed under a Creative Commons Attribution 4.0 International License, which permits use, sharing, adaptation, distribution and reproduction in any medium or format, as long as you give appropriate credit to the original author(s) and the source, provide a link to the Creative Commons license, and indicate if changes were made. The images or other third party material in this article are included in the article's Creative Commons license, unless indicated otherwise in a credit line to the material. If material is not included in the article's Creative Commons license and your intended use is not permitted by statutory regulation or exceeds the permitted use, you will need to obtain permission directly from the copyright holder. To view a copy of this license, visit http://creativecommons.org/licenses/by/4.0/
